# Efficacy and safety of chinese herbal medicine for treating mild or moderate COVID-19: A systematic review and meta-analysis of randomized controlled trials and observational studies

**DOI:** 10.3389/fphar.2022.988237

**Published:** 2022-09-07

**Authors:** Hongfei Zhu, Mengting Li, Chen Tian, Honghao Lai, Yuqing Zhang, Jiaheng Shi, Nannan Shi, Hui Zhao, Kehu Yang, Hongcai Shang, Xin Sun, Jie Liu, Long Ge, Luqi Huang

**Affiliations:** ^1^ Department of Social Medicine and Health Management, School of Public Health, Lanzhou University, Lanzhou, China; ^2^ Evidence Based Social Science Research Centre, School of Public Health, Lanzhou University, Lanzhou, China; ^3^ Department of Health Research Methods, Evidence, and Impact, McMaster University, Hamilton, ON, Canada; ^4^ CEBIM (Center for Evidence Based Integrative Medicine)-Clarity Collaboration, Guang’ Anmen Hospital, China Academy of Chinese Medical Sciences, Beijing, China; ^5^ Institute of Acupuncture and Moxibustion, China Academy of Chinese Medical Sciences, Beijing, China; ^6^ Nottingham Ningbo GRADE Center, The University of Nottingham Ningbo, Ningbo, China; ^7^ China Center for Evidence Based Traditional Chinese Medicine, China Academy of Chinese Medical Sciences, Beijing, China; ^8^ Department of Emergency, Guang’ Anmen Hospital, China Academy of Chinese Medical Sciences, Beijing, China; ^9^ Evidence-Based Medicine Center, School of Basic Medical Sciences, Lanzhou University, Lanzhou, China; ^10^ WHO Collaborating Center for Guideline Implementation and Knowledge Translation, Lanzhou, China; ^11^ Key Laboratory of Evidence Based Medicine and Knowledge Translation of Gansu Province, Lanzhou, China; ^12^ Key Laboratory of Chinese Internal Medicine of Ministry of Education, Beijing, China; ^13^ Dongzhimen Hospital, Beijing University of Chinese Medicine, Beijing, China; ^14^ Chinese Evidence-Based Medicine Center, West China Hospital, Sichuan University, Chengdu, China; ^15^ Department of Oncology, Guang’ Anmen Hospital, China Academy of Chinese Medical Sciences, Beijing, China; ^16^ National Resource Center for Chinese Materia Medica, China Academy of Chinese Medical Sciences, Beijing, China

**Keywords:** Chinese herbal medicine, COVID-19, Traditional Chinese Medicine, systematic review, meta-analysis

## Abstract

**Background:** The coronavirus disease 2019 (COVID-19) is still a pandemic globally, about 80% of patients infected with COVID-19 were mild and moderate. Chinese herbal medicine (CHM) has played a positive role in the treatment of COVID-19, with a certain number of primary studies focused on CHM in managing COVID-19 published. This study aims to systematically review the currently published randomized controlled trials (RCTs) and observational studies (OBs), and summarize the effectiveness and safety of CHM in the treatment of mild/moderate COVID-19 patients.

**Methods:** We searched 9 databases up to 19 March 2022. Pairs of reviewers independently screened literature, extracted data and assessed risk of bias. For overall effect, we calculated the absolute risk difference (ARD) of weighted averages of different estimates, and certainty of evidence was assessed using the Grading of Recommendations, Assessment, Development, and Evaluations (GRADE) system.

**Results:** We included 35 RCTs and 24 OBs enrolling 16,580 mild/moderate patients. The certainty of evidence was very low to low. Compared with usual supportive treatments, most effect estimates of CHM treatments were consistent in direction. CHMs presented significant benefits in reducing rate of conversion to severe cases (ARD = 99 less per 1000 patients in RCTs and 131 less per 1000 patients in OBs, baseline risk: 16.52%) and mortality (ARD = 3 less per 1000 patients in RCTs and OBs, baseline risk: 0.40%); shortening time to symptom resolution (3.35 days in RCTs and 2.94 days in OBs), length of hospital stay (2.36 days in RCTs and 2.12 days in OBs) and time to viral clearance (2.64 days in RCTs and 4.46 days in OBs); increasing rate of nucleic acid conversion (ARD = 73 more per 1000 patients in OBs, baseline risk: 16.30%). No serious adverse reactions were found and the differences between CHM and usual supportive care were insignificant.

**Conclusion:** Encouraging evidence showed that CHMs were beneficial in treating mild or moderate patients. CHMs have been proved to possess a safety profile that is comparable to that of usual supportive treatment alone. More rigorously designed clinical trials and mechanism studies are still warranted to further confirm the present findings.

## 1 Introduction

Since December 2019, the number of Coronavirus disease 2019 (COVID-19) infections has increased rapidly and in March 2020, the World Health Organization (WHO) declared it to be a global pandemic (WHO, 2020). As of 17 April 2022, there have been more than 500 million confirmed cases and more than 6 million deaths globally (WHO, 2022a). About 80% of patients infected with COVID-19 were mild and moderate, so it was critical for their effective management (NHC, 2020). After evaluating 463 randomized controlled trials (RCTs) and 180 drugs on non-severe COVID patients, WHO guideline (Agarwal et al., 2020) only made strong recommendations for nirmatrelvir and ritonavir, and conditional recommendations for molnupiravir, sotrovimab, remdesivir, casirivimab and imdevimab.

Chinese herbal medicine (CHM), an important part of traditional medicine, has been spread to more than 170 countries (WHO, 2013) and played a huge role in the management of COVID-19. According to the *WHO Expert Meeting on Evaluation of Traditional Chinese Medicine in the Treatment of COVID-19* released in March 2022, 12 selected RCTs demonstrated that Chinese patent medicine (CPM) as an additional intervention could shorten the time to viral clearance, resolution of clinical symptoms, and length of hospital stay compared with conventional treatment for mild-to-moderate patients (WHO, 2022b). However, in addition to this, there were many observational studies (OBs) on CPM, as well as RCTs or OBs on CHM. Therefore, it is crucial to timely summarize and evaluate all existed CHM evidence, including RCTs and OBs, to reflect the effectiveness and safety of CHM in the treatment of mild or moderate COVID-19 patients and further improve treatment measures and medical care worldwide.

## 2 Methods

We conducted and reported this systematic review and meta-analysis according to the Preferred Reporting Items for Systematic Reviews and Meta-Analyses (PRISMA) checklist (Page et al., 2021).

### 2.1 Literature search

An extremely sensitive search strategy that only included search terms related to disease (COVID-19) and study design were used to identify all relevant primary studies under the guidance of an experienced librarian, regardless of languages or types of publication. We conducted a systematic search from December 2019 to 19th March 2022 of the following 9 databases: PubMed, Embase, Cochrane Central Register of Controlled Trials (CENTRAL), Web of Science, China national knowledge infrastructure (CNKI), WanFang Database, Chinese BioMedical Literature Database (CBM), China Science and Technology Journal Database (VIP), and the L-OVE COVID-19 Repository. We also tracked the references of relevant publications. The details of search strategies can be found in Supplementary Table S1.

### 2.2 Eligibility criteria

Inclusion criteria: 1) Patients: confirm diagnosed mild or moderate COVID-19 according to the national or international recognized diagnosis standard; studies that included patients with both non-severe and severe or critical COVID-19 were eligible if more than 80% of patients were mild and/or moderate; studies included results of mild or moderate patients could be extracted from subgroup-analysis were also eligible. 2) Intervention: usual supportive treatment (e.g., bed rest, antibacterial treatment, antiviral treatment, immunotherapy, prone position treatment, etc.) any form of Chinese herbal medicine, such as granules, decoction and injections, were all considered to be included. Usual supportive treatment plus the combination of Chinese herbal medicines were also eligible. 3) Control: patients of the control group were given usual supportive treatment. 4) Outcomes: we decided the outcomes of interest according to a living network meta-analysis published in *BMJ (* *Siemieniuk et al., 2020* *)* and a core outcome set of COVID-19 (Jin X. et al., 2020); mainly including: a. clinical efficacy (e.g., mortality, viral clearance, length of hospital stay, rate of mechanical ventilation), b. clinical symptoms recovery (e.g., fever, cough, tiredness), c. adverse events (e.g., nausea and vomit, diarrhea, abnormal liver function). The study that reported at least one outcome listed above was considered eligible, 5) Study types: RCTs and OBs (e.g., cohort study and historical control study).

We excluded studies that enrolled 20% or more severe/critical patients, that did not report the outcome of interest, that were short reports or abstracts of which with key information missing, that did not report the approval information by the ethics committee or information about informed consent of patients, and that the study design was protocol, case report, case report series, cross-sectional study and controlled before-after study.

### 2.3 Study selection

EndNote X8.0 was used to manage the initially searched records. After removing duplicate records, the remaining records were imported into an online reference management software Rayyan (Ouzzani et al., 2016). After receiving training and calibration exercises, four teams of 2 reviewers (ZHF and LMT, SMY and TC, LYF and ZWZ, RSM and JJY) independently screened the title and abstracts of each record, then further reviewed full texts of potentially eligible studies to determine the final eligibility. Any conflict was resolved through discussion or consultation with a third reviewer (GL).

### 2.4 Data extraction

A standardized, pilot tested data extraction form was used to extract information from each eligible trial. Teams of 2 reviewers (LYF and YQY, SMY and TC, CX and LHH), following training and calibration exercises, independently extracted data of interest, including 1) trial characteristics: first author, year of publication, trial registration number, published journal, language, study design and funding source; 2) baseline patient characteristics: geographic location and recruitment timeline of the study, age, gender, proportion of morbidities at baselines; 3) baseline clinical characteristics: type, dose and duration of care, details of CHM components, severity of COVID-19 symptoms; and 4) outcomes of interest: means or medians and measures of variability for continuous outcomes, and the number of patients analyzed and the number of patients who experienced relevant event for dichotomous outcomes. When eligible studies did not report interest data, we would contact authors to obtain data.

### 2.5 Risk of bias assessment

The risk of bias of eligible RCTs was independently assessed using a modified Cochrane risk of bias tool (RoB 2.0) (JAC et al., 2019) based on 6 domains: bias from the randomization process generated, bias due to deviations from the intended intervention, bias due to missing data, bias in measurement of the outcome, bias in selection of the reported results and bias due to other sources (e.g. consistency between the registration information and the final report, completeness of the report). For included OBs, a modified Risk of Bias in Non-randomized Studies-of Interventions (ROBINS-I) (Sterne et al., 2016) was used to assess the risk of bias according to the following 6 domains: bias due to confounding, bias in selection of participants into the study; bias from the interventions, bias due to missing data, bias due to measurement of the outcome, and bias in selection of the reported results.

Each item for included studies was categorized into four groups: low risk of bias, probably low risk of bias, probably high risk of bias, and high risk of bias. When information reported by primary studies was insufficient for reviewers to assess the risk of bias, we would contact authors for more adequate information. Discrepancies were solved by discussion and, when necessary, with adjudication by a third reviewer (GL). Detailed guidance for assessment of risk of bias was presented in Supplementary Table S2.

### 2.6 Evidence synthesis

When there were two or more studies with the same study design, intervention and control treatments reporting on the same outcome measure, Review Manager software (RevMan, version 5.3, Copenhagen: The Nordic Cochrane Centre, The Cochrane Collaboration, 2014) was used to perform meta-analysis. Where quantitative meta-analysis was unfeasible, qualitative systematic analysis was conducted to show the difference. The efficacy trend and distribution of point estimates of different Chinese medicines were visually presented with a forest plot. The direction and distribution of effect estimates of different CHM treatments across studies were compared to show the benefit and harm of CHM compared with usual supportive treatment. For dichotomous data, risk ratio (RR) with corresponding 95% confidence interval (CI) was calculated. For continuous data, we used the mean difference (MD) with 95% CI. When eligible study was unable to include in meta-analysis, if there existed OB that reported the results with adjustment of confounders, we directly used effect measures and effect estimates reported in OB. Due to the differences arising from the study design, we presented the analysis results of RCTs and OBs separately and did not merge the results.

In order to obtain the overall effect of CHM treatments, we also calculated weighted averages of different effect estimates using the inverse variance method, and presented the absolute risk difference (ARD) for weighted averages. We used a random model if there was considerable variation between studies, otherwise, we used a fixed model. To calculate ARD with corresponding 95% CI, wherever possible, we used the baseline risk from the WHO living guideline (Agarwal et al., 2020) for the corresponding outcome, otherwise, we calculated the median incidence rate in the usual supportive care group from studies with the same study design (More details of baseline risk were shown in Supplementary Table S3). Considering statistical power, we performed meta-regression, sensitivity analysis, and publication bias checks using Stata v.16.0 software (Stata Corporation LLC, College Station, United States) when there were10 or more studies included. The inter-study heterogeneity was examined by using standard Cochran’s Q test and I^2^ statistic. When there existed substantial heterogeneity, we conducted meta-regression analysis to explore the source of heterogeneity based on the following 2 factors: whether comorbidities are reported and the proportion of mild and moderate COVID-19 patients. Sensitivity analysis was undertaken by random effect models to observe the robustness of result. For the analysis that included 10 or more study, we evaluated the publication bias through Egger’s linear regression test (Egger et al., 1997) and funnel plot. *p* < 0.05 was considered as statistical significance.

Rader chart was used to show the rate of adverse reactions which were reported commonly in both RCTs and non-randomized trials, and each axis represented an adverse reaction. We would perform subgroup analyses by severity of patients, age, and comorbidities if the number of studies was sufficient.

### 2.7 Certainty of evidence assessment

For the weighted averages of different effect estimate, two reviewers (ZHF and LMT) independently assessed the certainty of evidence using the Grading of Recommendations, Assessment, Development, and Evaluations (GRADE) system (Guyatt et al., 2008). The certainty of evidence was categorized as high, moderate, low or very low. At the beginning of the assessment, RCTs started as high certainty and could be downgraded due to five reasons: risk of bias, imprecision, inconsistency, indirectness, and publication bias; OBs started as low certainty, which could be downgraded due to five reasons same as RCTs, and upgraded due to three reasons: large magnitude of an effect, dose-response gradient, and effect of plausible residual confounding.

## 3 Results

**FIGURE 1:**
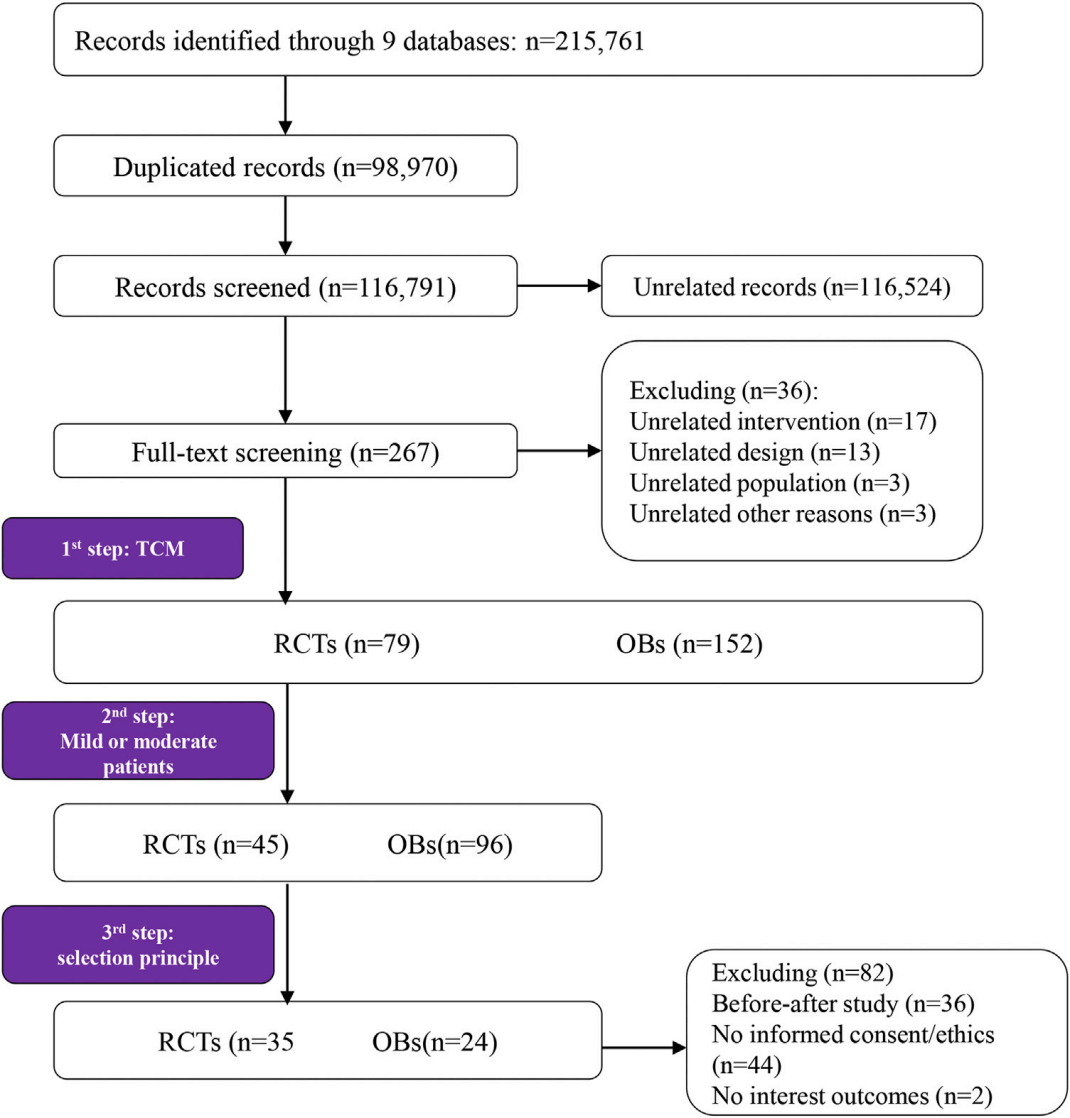
Flow diagram of study selection and identification.

In total, 215,761 records were derived from electronic databases, of which 267 were potentially eligible and further underwent full-text screening. First, we obtained 231 primary studies involving drugs and non-drug treatments for the prevention, treatment, and rehabilitation stages. In the second step, 45 RCTs and 96 OBs focused on patients with mild or/and moderate COVID-19 patients were identified. In the third step, we included 35 RCTs and 24 OBs according to the eligibility criteria. The literature screening process was shown in Figure 1.

### 3.1 Characteristics of included studies

We included thirty-five RCTs enrolled 4,166 mild or moderate patients (male: 50.99%), with a mean age of 49.08 years. There were 22 studies published in Chinese (Ai X. Y. et al., 2020; Jin W. et al., 2020; Zhang C. T. et al., 2020; AI et al., 2020b; Yu P. et al., 2020; Wang Y. L. et al., 2020; Ding et al., 2020; Duan et al., 2020; Zhang Y. L. et al., 2020; Fu et al., 2020; Lin et al., 2020; Sun et al., 2020; Hu F. et al., 2021; Wang L. Q. et al., 2021; Wang Y. et al., 2021; He et al., 2021; Li et al., 2021; Ping et al., 2021; Qiu et al., 2021; Sun S et al., 2021; Wu et al., 2021; Yan et al., 2021) (2 registered prospectively (Jin W. et al., 2020; Sun Y et al., 2021)) and 13 published in English which were all registered (Xiao M. Z. et al., 2020; Wang J. B. et al., 2020; Xiong et al., 2020; Liu J. et al., 2021; An et al., 2021; Hu K. et al., 2021; Zhang X. Y. et al., 2021; Ma et al., 2021; Ni et al., 2021; Xu et al., 2021; Zeng et al., 2021; Zhao et al., 2021; Zhang et al., 2022). Twenty-four OBs(Chen et al., 2020a; Yang M. B. et al., 2020; Yu H. Y. et al., 2020; Chen et al., 2020b; Xiao Q. et al., 2020; Zhang C. Y. et al., 2020; Ai Z. Z. et al., 2020; Zhang N. et al., 2020; Lan et al., 2020; Li, 2020; Liu et al., 2020; Xin et al., 2020; Yao et al., 2020; Zeng et al., 2020; Chen J. et al., 2021; Zhang L. H. et al., 2021; Chen R. B. et al., 2021; Liu L. et al., 2021; Wang Q. L. et al., 2021; Shi et al., 2021; Sun S et al., 2021; Feng et al., 2022) (Tian et al., 2020; Zhang X. et al., 2021) enrolled 12,414 mild or moderate patients (male: 48.11%), with a mean age of 54.53 years. Fourteen OBs were published in Chinese (1 registered prospectively (Chen R. B. et al., 2021)) and 10 were published in English (4 registered prospectively (Tian et al., 2020; Xin et al., 2020; Zhang X. et al., 2021; Shi et al., 2021)).

All patients were recruited from China, of which 59.32% were in Hubei (Chen et al., 2020a; Xiao M. Z. et al., 2020; Yang M. B. et al., 2020; Yu H. Y. et al., 2020; Zhang C. T. et al., 2020; Chen et al., 2020b; Xiao Q. et al., 2020; Yu P. et al., 2020; Zhang C. Y. et al., 2020; Ai Z. Z. et al., 2020; Ding et al., 2020; Duan et al., 2020; Zhang Y. L. et al., 2020; Lan et al., 2020; Li, 2020; Tian et al., 2020; Xin et al., 2020; Xiong et al., 2020; Yao et al., 2020; Chen J. et al., 2021; Hu F. et al., 2021; Liu J. et al., 2021; An et al., 2021; Wang L. Q. et al., 2021; Zhang L. H. et al., 2021; Hu K. et al., 2021; Liu L. et al., 2021; Wang Y. et al., 2021; He et al., 2021; Ni et al., 2021; Shi et al., 2021; Sun S et al., 2021; Sun Y et al., 2021; Xu et al., 2021; Zhao et al., 2021). Except for 1 OB recruited from July to September 2020 (Wang Q. L. et al., 2021), the other studies were recruited at the beginning of the outbreak (December 2019 to May 2020). The most common comorbidities were diabetes, hypertension, cardiovascular disease or coronary heart disease, and respiratory conditions in eligible studies. In addition, there was one study on patients with COVID-19 and hepatitis B. Supplementary Table S4 presents the detailed study characteristics.

Fifty-nine studies involved 38 drugs, including Qingfei Paidu decoction (QFPD; 1 RCT and 7 OBs), Lianhua Qingwen granules/capsules (LHQW; 3 RCTs and 3 OBs), Jinyinhua oral liquid (JYH; 2 RCTs), Jinhua Qinggan granules (JHQG, 2 RCTs and 1 OB), Huashi Baidu granule (2 RCTs and 1 OB), Reduning injection (RDN, 2 RCTs), Shufeng Jiedu capsules (SFJD, 3 OBs), FeiyanYihao Chinese medicine granules (FY 1, 1 RCT and 1 OB), Lianhua Qingke granules (LHQK, 2 RCTs), Toujie Quwen granules (TJQW, 2 RCTs). In addition, there were CHMs only reported in one study: Buzhong YiQi decoction (BZYQ), Compound Yinchai Granules (FFCY) and Qingqiao Jiedu Granules (QQJD), Gegen Qinlian pill (GGQL), Ganlu Xiaodu decoction (GLXD), Hanshiyi Formula (HSY), Jiawei Dayuan decoction (JWDY), Keguan-1 decoction, Liushen pill (LS), Maxingshigan-Weijing decoction (MWD), Ma Xing Shigan decoction (MXSG), Maxin Xuanfei Jiedu decoction (MXXF), Qingfei Touxie Fuzheng decoction (QFTXFZ), Qingre Kangdu oral liquid (QRKD) and Lanxiang Jiedu oral liquid (LXJD), Qushi Paidu Fuzheng Recipe (QSPDFZ), Reyanning injection (RYN), Sanao Yulong mixture (SAYL), Self-formulated Compatible Decoction, Shuanghuanglian oral liquid (SHL), Tanreqing Capsule (TRQ), Xuebijing injection (XBJ), Xuanfei Baidu decoction (XFBD), Xuanfei Qingre decoction (XFQR), Xiyanping injection (XYP), Xueshuantong injection (XST), Yindan Jiedu granules (YDJD), and Yinghuang Qingfei capsules (YHQF).

The usual supportive treatment was performed mainly according to the Chinese treatment regimens recommended by the *“Diagnosis and Treatment Protocol for COVID-19”* (3rd to 7th Edition), including bed rest, monitoring life sign, oxygen therapy, prone position therapy, immunotherapy, antiviral, antibacterial, and anticoagulation therapy. The specific components of CHM, the measures of the control group and the intervention group were shown in Supplementary Table S5.

### 3.2 Risk of bias in included studies

**FIGURE 2:**
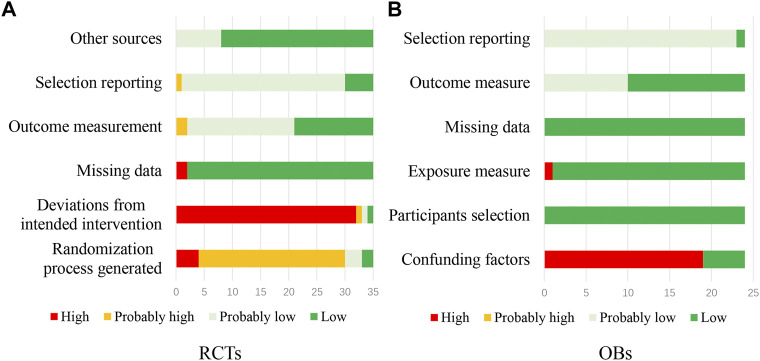
The risk of bias for RCTs **(A)** and OBs **(B)**.

Figure 2 showed the risk of bias for RCTs (a) and OBs (b). Assessment of the risk of bias for single RCTs was presented in Supplementary Table S6. Only 3 RCTs (Wang J. B. et al., 2020; Zhang X. Y. et al., 2021; Sun Y et al., 2021) were assessed at low or probably low risk of bias in all domains. Other studies were assessed as high or probably high risk in at least one domain. The main biases were from random sequence generation and deviations from the intended intervention. Two RCTs (Xiao M. Z. et al., 2020; Xiong et al., 2020) also had risks with the missing data and subjective measurement of outcomes.

Assessment of the risk of bias for single OBs was presented in Supplementary Table S7. Four OBs (Chen et al., 2020a; Chen et al., 2020b; Tian et al., 2020; Zhang L. H. et al., 2021; Feng et al., 2022) were at low or probably low risk of bias in all domains. Other OBs were assessed as high risk of bias mainly because they did not adjust for confounding.

### 3.3 Efficacy of Chinese herbal medicine interventions

#### 3.3.1 Rate of conversion to severe cases

Twenty-three studies (16 RCTs, 7 OBs enrolled 4287mild/moderate patients) reported the rate of conversion to severe cases, involving 23 CHMs. We used forest plot to present the distribution of effects among included 24 studies (see Supplementary Figure S1.1 for RCTs, Supplementary Figure S1.2 for OBs). The point estimates of all studies showed consistent direction that CHMs were beneficial in reducing the rate of conversion of mild/moderate to severe cases and 9 CHMs showed statistical significance. Evidence from RCTs showed that compared with usual supportive care, LHQW (RR = 0.62, 95% CI: 0.40–0.94), JYH (RR = 0.08, 95% CI: 0.01, 0.64), JHQG (RR = 0.67, 95% CI: 0.15–2.98) and HSBD (RCT: RR = 0.31, 95% CI: 0.12–0.84) could significantly reduce the rate of conversion to severe cases. Evidence from OBs showed that QFPD (RR = 0.30, 95% CI: 0.14–0.63), LHQW (RR = 0.45, 95% CI: 0.25–0.80), JYBD (RR = 0.18, 95% CI: 0.04–0.70), QSPDFZ (RR = 0.23, 95% CI: 0.11–0.51), HSY (RR = 0.02, 95% CI: 0.00–0.29) and GLXD (RR = 0.03, 95% CI: 0.00–0.42) could significantly reduce the rate of conversion to severe cases.

The weighted averages of different effect estimates of RCTs and OBs showed that CHMs could significantly reduce the rate of conversion to severe cases by 58% (RR = 0.40, 95%CI: 0.29 to 0.57; ARD = 99 less per 1000 patients, 95%CI: 71 less to 117 less; low certainty) (see Supplementary Figure S1.1) and 80% (RR = 0.21, 95%CI: 0.15 to 0.30; ARD = 131 less per 1000 patients, 95%CI: 116 less to 140 less; very low certainty) (see Supplementary Figure S1.2) respectively. Table 1 showed the GRADE summary of finding table. Egger’s test indicated the possibility of publication bias (RCTs: *p* = 0.001; OBs: *p* = 0.002, Supplementary figure S10.1-2).

**TABLE 1:** GRADE summary of findings table showing certainty of evidence of weighted averages of different effect estimates on health outcomes in mild/moderate COVID-19 patients.

Outcome	Study design	No. of studies	No. of patients	Effect (95%CI)	Certainty of evidence
Rate of conversion to severe cases	RCT	16	2472	**RR 0.40 (0.29 to 0.57) ARD 99 less per 1000 patients (71 less to 117 less)**	Moderate^a^
	OB	7	1815	**RR 0.21 (0.15 to 0.30) ARD 131 less per 1000 patients (116 less to 140 less)**	Very low^a^
Length of hospital stay	RCT	12	1585	**MD-2.36 days (-3.53 to -1.18)**	Low^a^ ^,^ ^b^
	OB	11	1683	**MD-2.12 days (-3.82 to -0.42)**	Very low^a^ ^,^ ^b^
Time to viral clearance	RCT	19	1436	**MD-2.64 days (-3.93 to -1.35)**	Low^a^ ^,^ ^b^
	OB	9	1180	**MD -4.46 days (-5.02 to -3.89)**	Very low^a^
Rate of nucleic acid conversion	RCT	7	957	RR 1.12 (0.99–1.27) ARD 20 more per 1000 patients (2 less to 44 more)	Low^a^ ^,^ ^c^
	OB	6	450	**RR 1.45 (1.07 to 1.95) ARD 73 more per 1000 patients (11 more to 155 more)**	Very low^a^
Rate of mortality	RCT	7	1113	RR 0.23 (0.06–0.89) ARD 3 less per 1000 patients (0.4 less to 3.8 less)	Low^a^ ^,^ ^c^
	OB	2	9002	**RR 0.24 (0.17 to 0.35) ARD 3 less per 1000 patients (2.6 less to 3.3 less)**	Very low^a^

Bold indicates statistical significance.

a Downgraded due to risk of bias.

b Downgraded due to inconsistency.

c Downgraded due to imprecision.

#### 3.3.2 Time to symptom resolution

Twenty-two studies (12 RCTs and 10 OBs enrolled 1867 mild/moderate patients) reported the time to fever resolution, involving 18 CHMs. We used forest plot to present the distribution of effects among included 22 studies (see Supplementary Figure S2.1 for RCTs, Supplementary Figure S3.1 for OBs). The point estimates of most studies showed consistent direction that CHMs were beneficial in shortening the time to fever resolution and 12 CHMs showed statistical significance among them. Evidence from RCTs showed that compared with usual supportive care, LHQW (MD = −1.00 days, 95%CI: −1.25 to −0.75), YHQF (MD = −0.90 days, 95%CI: −1.00 to −0.80), LS (MD = −2.67 days, 95%CI: 4.59 to −0.75), Keguan-1 (MD = −1.75 days, 95%CI: −2.69 to −0.81), MWD (MD = −4.00 days, 95%CI: −6.34 to −1.66), SAYL (MD = −1.00 days, 95%CI: −1.69 to −0.31), MXXF (MD = −1.68 days, 95%CI: −2.38 to −0.98) and JWDY(MD = −2.52 days, 95%CI: −3.30 to −1.74) could significantly shorten the time to fever resolution. Evidence from OBs showed that SFJD (MD = −0.84 days, 95%CI: −1.09 to −0.59), GLXD (MD = −1.77 days, 95%CI: −2.42 to −1.12), TJQW (MD = −3.80 days, 95%CI: −4.47 to −3.13) and Self-formulated Compatible Decoction (MD = −1.08 days, 95%CI: −1.58 to −0.58) could significantly shorten the time to fever resolution.

The weighted averages of different effect estimates of RCTs showed that CHMs could significantly shorten the time to time to total symptom, fever, cough, tiredness and shortness of breath resolution. In addition, the weighted averages of different effect estimates of OBs indicated that CHMs could shorten the time to fever, cough, tiredness, expectoration and sore throat resolution (See Supplementary Figure S2.1–2.5 for RCTs, Supplementary Figure S3.1–3.6 for OBs). Table 2 shows the weighted averages of different effect estimates.

**TABLE 2:** GRADE summary of findings table showing certainty of evidence of weighted averages of different effect estimates on time to symptom resolution (days) in mild/moderate COVID-19 patients.

Symptom	Study design	No. of studies	No. of patients	Effect estimates (MD, 95%CI)	Certainty of evidence
Total	RCT	5	668	**−3.35 (−4.68 to −2.03)**	Moderate^a^
	OB	1	80	**−**2.94 (**−**6.67 to 0.78)	Very low^a^ ^,^ ^c^
Fever	RCT	12	795	**−1.19 (−1.54 to −0.84)**	Moderate^a^
	OB	10	1062	**−1.31 (−1.95 to −0.68)**	Very low^a^ ^,^ ^b^
Cough	RCT	7	574	**−2.55 (−3.59 to −1.52)**	Very low^a^ ^,^ ^b^
	OB	8	1114	**−1.11 (−1.82 to −0.41)**	Very low^a^
Tiredness	RCT	4	308	**−1.62 (−2.99 to −0.25)**	Moderate^a^
	OB	7	725	**−1.06 (−1.66 to −0.46)**	Very low^a^
Shortness of breath	RCT	2	104	**−3.55 (−5.99 to −1.11)**	Moderate^a^
Expectoration	OB	3	100	**−3.19 (−3.95 to −2.43)**	Very low^a^
Sore throat	OB	3	268	**−1.07 (−1.92 to −0.22)**	Very low^a^

Bold indicates statistical significance.

a Downgraded due to risk of bias.

b Downgraded due to inconsistency.

c Downgraded due to imprecision.

#### 3.3.3 Length of hospital stay

Twenty-three studies (12 RCTs and 11 OBs enrolled 3268 mild/moderate patients) reported the length of hospital stay, involving 17 CHMs. We used forest plot to present the distribution of effects among included 23 studies (see Supplementary Figure S4.1 for RCTs, Supplementary Figure S4.2 for OBs). The point estimates of most studies showed consistent direction that CHMs were beneficial in shortening the length of hospital stay and 10 CHMs showed statistical significance among them. Evidence from RCTs showed that compared with usual supportive care, QFPD (MD = −3.10 days, 95%CI: −3.72 to −2.48), JYH(MD = −6.16 days, 95%CI: −8.46 to −3.86), RDN (MD = −3.23 days, 95%CI: −4.92 to −1.55), JWYPF(MD = −8.17 days, 95%CI: −10.76 to −5.58), TJQW (MD = −2.70 days, 95%CI: −4.64 to −0.76), SAYL (MD = −1.00 days, 95%CI: −1.69 to −0.35) and XFQR (MD = −3.21 days, 95%CI: −6.08 to −0.34) could significantly shorten the length of hospital stay. Evidence from OBs showed that QFPD (MD = −2.74 days, 95%CI: −5.41 to −0.07), MXSG (MD = −2.85 days, 95%CI: −3.76 to −1.94), GLXD (MD = −1.10 days, 95%CI: −2.04 to −0.16) and Self-formulated Compatible Decoction (MD = −4.92 days, 95%CI: −5.83 to −4.01) could significantly shorten the length of hospital stay.

The weighted averages of different effect estimates of RCTs and OBs showed that CHMs could significantly shorten the length of hospital stay by −2.36 days (MD = −2.36 days, 95%CI: −3.53 to −1.18; very low certainty) (see Supplementary Figure S4.1) and 2.12 day (MD = −2.12 days, 95%CI: −3.82 to −0.42; very low certainty) (see Supplementary Figure S4.2). For RCTs, Egger’s test indicated the possibility of publication bias (RCTs: *p* = 0.039; OBs: *p* = 0.02, Supplementary Figure S10.3–4). The results of meta-regression demonstrated that comorbidities (*p* = 0.360), proportion of mild (*p* = 0.472) and moderate (*p* = 0.547) COVID-19 patients might not be the potential sources of heterogeneity (see Supplementary Figure S11.1). Sensitivity analysis revealed no outlier studies that might significantly alter the primary results (see Supplementary Figure S12.1-2).

#### 3.3.4 Time to viral clearance

Twenty-two studies (13 RCTs, 9 OBs enrolled 2616 mild/moderate patients) reported the time to viral clearance, involving 18 CHMs. We used forest plot to present the distribution of effects among included 22 studies (see Supplementary Figure S5.1 for RCTs, Supplementary Figure S5.2 for OBs). The point estimates of most studies showed consistent direction that CHMs were beneficial in shortening the time to viral clearance and 14 CHMs showed statistical significance among them. Evidence from RCTs showed that compared with usual supportive care, LHQW (MD = −1.34 days, 95%CI: −1.96 to −0.72), JYH (MD = −5.74 days, 95%CI: −7.77 to −3.71), RDN (MD = −3.75 days, 95%CI: −4.27 to −3.24), GGQL (MD = −2.17 days, 95%CI: −3.61 to −0.73), JWYPF(MD = −6.83 days, 95%CI: −9.10 to −4.56), XYP (MD = −3.53 days, 95%CI: −5.59 to −1.47), SAYL (MD = −2.00 days, 95%CI: −3.54 to −0.46) and XFQR (MD = −3.15 days, 95%CI: −5.44 to −0.86) could significantly shorten the time to viral clearance. Evidence from OBs showed that QFPD (MD = −4.04 days, 95%CI: −5.15 to −2.94), JHQG (MD = −3.00 days, 95%CI: −4.76 to −1.24), MSXG (MD = −3.90 days, 95%CI: −4.96 to −2.84), TJQW (MD = −5.66 days, 95%CI: −6.39 to −4.93), YDJD (MD = −5.02 days, 95%CI: −5.36 to −4.68) and TRQ (MD = −3.93 days, 95%CI: −7.50 to −0.36) could significantly shorten the time to viral clearance.

The weighted averages of different effect estimates of RCTs and OBs showed that CHMs could significantly shorten the time to viral clearance by 2.64 days (MD = −2.64 days, 95%CI: −3.93 to −1.35; low certainty) (see Supplementary Figure S5.1) and 4.46 days (MD = −4.46 days, 95%CI: −5.02 to −3.89; very low certainty) (see Supplementary Figure S5.2) respectively. For RCTs, no publication bias was detected (*p* = 0.701, see Supplementary Figure S10.5). The results of meta-regression demonstrated that comorbidities (*p* = 0.265), proportion of mild (*p* = 0.472) and moderate (*p* = 0.79) COVID-19 patients might not be the potential sources of heterogeneity (see Supplementary Figure S11.3). Sensitivity analysis of RCTs revealed no outlier studies that might significantly alter the primary results for RCTs (see Supplementary Figure S12.3).

#### 3.3.5 Rate of nucleic acid conversion

Thirteen studies (7 RCTs, 6 OBs enrolled 1407 mild/moderate patients) reported the rate of nucleic acid conversion, involving 10 CHMs. We used forest plot to present the distribution of effects among included 13 studies (see Supplementary Figure S6.1 for OBs, Supplementary Figure S6.2 for RCTs). The point estimates of most studies showed consistent direction that CHMs were beneficial in increasing the rate of nucleic acid conversion and 5 CHMs showed statistical significance among them. Evidence from OBs showed that compared with usual supportive care, JHQG (RR = 2.05, 95%CI: 1.14–3.68), RYN (RR = 1.58, 95%CI: 1.13, 2.21), QSPDFZ (RR = 3.74, 95%CI: 1.70–8.26) and QFDYG (RCT: RR = 1.20, 95%CI: 1.01–1.43) could significantly increase the rate of nucleic acid conversion. Evidence from RCTs showed that BZYQ (RR = 2.19, 95%CI: 1.33–3.59) could significantly increase the rate of nucleic acid conversion.

The weighted average of different effect estimates of OBs showed that CHMs could significantly increase the rate of nucleic acid conversion by 50% (RR = 1.45, 95%CI: 1.07 to 1.95; ARD = 73 more per 1000 patients, 95%CI: 11 more to 155 more; very low certainty) (see Supplementary Figure S6.1), and RCTs showed CHMs could increase rate of nucleic acid conversion by 12% (RR = 1.12, 95%CI: 0.99 to 1.27; ARD = 20 more per 1000 patients, 95%CI: 2 less to 44 more; low certainty) (see Supplementary Figure S6.2).

#### 3.3.6 Rate of mortality

Nine studies (7 RCTs, 2 OBs enrolled 10,115 mild/moderate patients) reported the rate of mortality, involving 7 CHMs. We used forest plot to present the distribution of effects among included 9 studies (see Supplementary Figure S7.1 for OBs, Supplementary Figure S7.2 for RCTs). The point estimates of all studies showed consistent direction that CHMs were beneficial in reducing the rate of mortality and 1 CHMs showed statistical significance among them. The weighted averages of different effect estimate of OBs and RCTs showed that QFPD could significantly reduce the rate of mortality by 76% (RR = 0.24, 95%CI: 0.17 to 0.35; ARD = 3 less per 1000 patients, 95%CI: 2.6 less to 3.3 less; very low certainty) (see Supplementary Figure S7.1) and 89% (RR = 0.23, 95%CI: 0.06 to 0.89; ARD = 3 less per 1000 patients, 95%CI: 0.4 less to 3.8 less; low certainty) (see Supplementary Figure S7.2).

#### 3.3.7 Rate of symptom resolution

The weighted averages of different effect estimates of RCTs showed that CHMs could significantly increase the rate of cough, tiredness, loss of appetite, shortness of breath resolution and CT improvement (see Supplementary Figures S8.1–S8.10). In addition, the weighted averages of different effect estimates of OBs indicated that CHMs could increase the rate of fever, tiredness, shortness of breath, chest tightness resolution and CT improvement (See Supplementary Figures S9.1–9.9). Table 3 shows the weighted averages of different effect estimates.

**TABLE 3:** GRADE summary of findings table showing certainty of evidence of weighted averages of different effect estimates on rate of symptom resolution in mild/moderate COVID-19 patients.

Symptom	Study design	No. of studies	No. of patients	Effect estimates (RR, 95%CI)	Absolute risk difference	Certainty of evidence
Fever	RCT	12	645	1.07 (0.99–1.16)	65 more per 1000 (9 less to 148 more)	Moderate^a^
	OB	5	288	**1.20 (1.09 to 1.31)**	185 more per 1000 (83 more to 286 more)	Very low^a^
Cough	RCT	14	1113	**1.21 (1.13 to 1.30)**	135 more per 1000 (84 more to 196 more)	Moderate^a^
	OB	5	352	1.21 (0.87–1.70)	135 more per 1000 (120 less to 646 more)	Very low^a^ ^,^ ^c^
Tiredness	RCT	14	756	**1.23 (1.13 to 1.33)**	159 more per 1000 (90 more to 229 more)	Moderate^a^
	OB	4	129	**1.31 (1.08 to 1.58)**	215 more per 1000 (55 more to 402 more)	Very low^a^
Expectoration	RCT	4	156	**1.38 (1.15 to 1.65)**	265 more per 1000 (105 more to 453 more)	Moderate^a^
	OB	3	86	**1.89 (1.39 to 2.57)**	621 more per 1000 (272 more to 1095 more)	Very low^a^ ^,^ ^c^
Loss of appetite	RCT	5	168	**1.28 (1.10 to 1.48)**	222 more per 1000 (79 more to 380 more)	Moderate^a^
	OB	2	28	1.14 (0.43–3.02)	111 more per 1000 (451 less to 1599 more)	Very low^a^ ^,c^
Shortness of breath	RCT	4	178	**1.44 (1.13 to 1.84)**	280 more per 1000 (83 more to 535 more)	Moderate^a^
	OB	2	44	**3.13 (1.44 to 6.81)**	1356 more per 1000 (280 more to 3697 more)	Very low^a^ ^,^ ^c^
Chest tightness	RCT	2	68	**2.75 (1.48 to 5.12)**	507 more per 1000 (139 more to 1194 more)	Low^a^ ^,^ ^c^
	OB	3	127	**1.41 (1.20 to 1.67)**	119 more per 1000 (58 more to 194 more)	Very low^a^
Chest tightness and shortness of breath	RCT	2	120	1.00 (0.83–1.22)	0 more per 1000 (133 less to 172 more)	Low^a^ ^,^ ^c^
Diarrhea	RCT	7	185	0.88 (0.70–1.11)	90 less per 1000 (225 less to 83 more)	Low^a^ ^,^ ^c^
	OB	3	28	0.98 (0.75–1.29)	15 less per 1000 (188 less to 218 more)	Very low^a^ ^,c^
CT improvement	RCT	10	1218	**1.24 (1.16 to 1.34)**	157 more per 1000 (105 more to 222 more)	Moderate^a^
	OB	11	1549	**1.21 (1.14 to 1.28)**	137 more per 1000 (92 more to 183 more)	Very low^a^

Bold indicates statistical significance.

a Downgraded due to risk of bias.

b Downgraded due to inconsistency.

c Downgraded due to imprecision.

### 3.4 Safety of Chinese herbal medicine interventions

**FIGURE 3:**
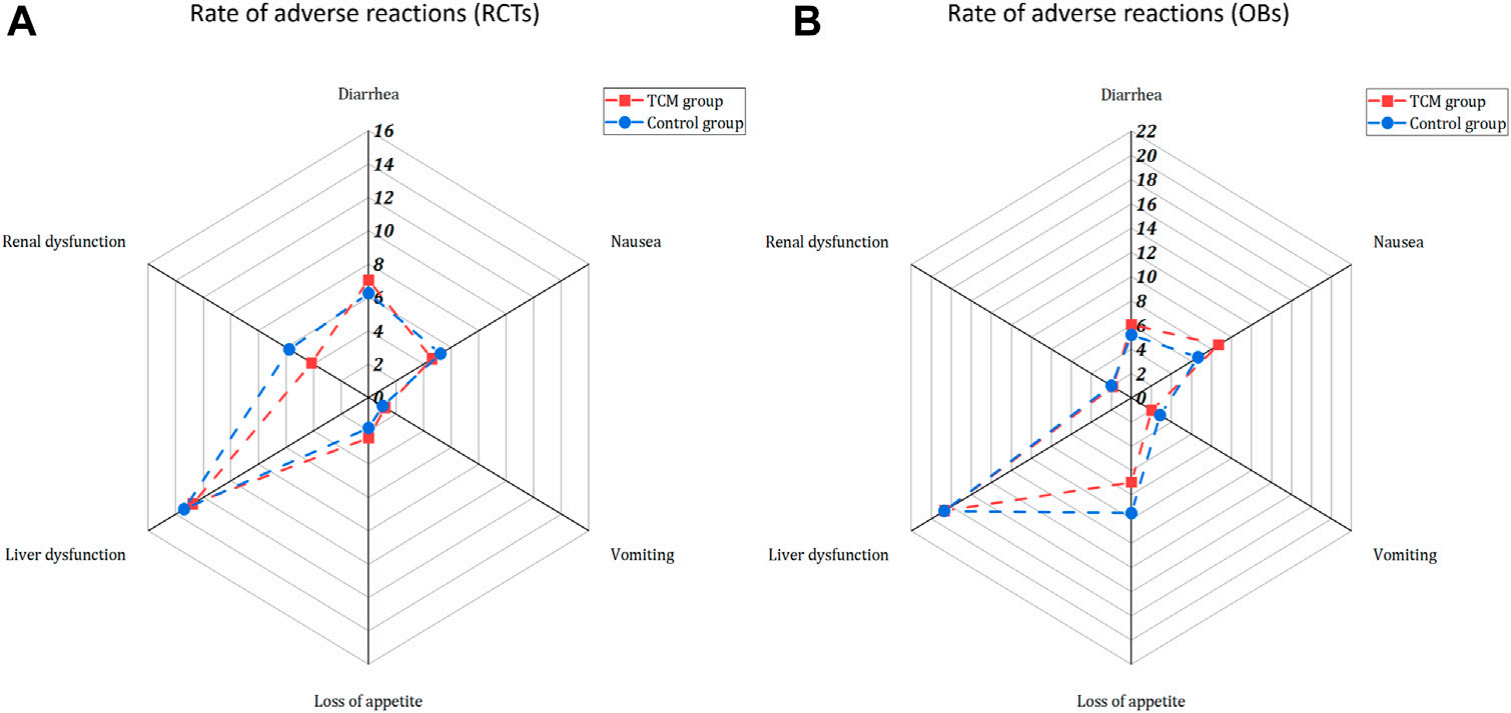
Rate of adverse reactions reported in RCTs **(A)** and OBs **(B)**.

Thirty-seven studies (21 RCTs, 16 OBs) including 13,695 patients reported 61 types of adverse events (See Supplementary Table S8 for incidence and difference between groups of adverse reactions). Compared with the usual supportive treatment, the LHQW group showed significant difference in reducing the incidence of diarrhea (5.63% vs. 13.38%). However, JHQG group showed significant difference in increasing the incidence of diarrhea (32.93 vs. 0.00%). Commonly, patients in the CHM group had a lower incidence rate among the other adverse reactions. Adverse reactions reported in 4 or more studies included diarrhea, nausea, vomiting, loss of appetite, liver dysfunction and renal dysfunction (Figure 3), but no adverse reactions except diarrhea showed a statistical difference.

## 4 Discussion

### 4.1 Main findings

CHM applications in treating infectious diseases through the long history of China, its efficacies were also shown in treating other types of viral infections including those caused by SARS-CoV (severe acute respiratory syndrome coronavirus), MERS-CoV (Middle East respiratory syndrome coronavirus) as well as Ebola virus. During the COVID-19 pandemic, the unique advantages of CHM were completely utilized in treating pandemics and combined with western medicine to make great contributions to the control of the pandemic in China. This review provided a comprehensive overview of the evidence for CHM treatments in mild/moderate patients with COVID-19 as of 19th March 2022, including 59 studies (35 RCTs, 24 OBs) enrolling 16,580 patients, 38 kinds of CHM were involved. The certainty of evidence was very low to low, with all evidence downgrading primarily due to the risk of bias. Therefore, the interpretation of results was mainly based on the analysis of RCTs. The results indicated that, for mild/moderate patients, compared to usual supportive treatment, CHM presented significant effects in various patient-important outcomes, especially in reducing the rate of conversion to severe cases. Preventing exacerbation is key in moderate COVID-19 patients’ treatment. When the non-severe patient base is large, the rate of conversion to severe cases will directly influence the number of severe patients. Meanwhile, CHM also showed advantages in reducing rate of mortality; shortening time to symptom (fever, cough, tiredness, expectoration, shortness of breath and sore throat) resolution, length of hospital stay and time to viral clearance; increasing rate of nucleic acid conversion, rate of symptom (fever, cough, tiredness, expectoration, loss of appetite, shortness of breath and chest tightness) resolution and CT improvement. In terms of adverse reactions, we did not find serious adverse events related to CHMs in both mild and moderate patients, which indicated that CHM may be relatively safe for mild/moderate COVID-19 patients. In addition to diarrhea, no adverse reactions showed significant difference between CHM group and control group.

Because of the incomplete reporting of included studies, we failed to perform subgroup analyses. However, the univariate regression analysis result of a cohort study (Tian et al., 2020) revealed sex (male), age, fever, cough, and fatigue as risk factors for progression to severe disease, and HSY Formula could significantly reduce the rate of conversion to severe cases, which may effectively prevent and treat the COVID-19. Another RCT performed a multivariate logistic regression analysis, which indicated that age and patients’ source (centralized isolation site) were independent risk factors for worsening during treatment, the rate of conversion to severe cases also showed a significant difference between HSBD granule group and control group (Zhao et al., 2021). The performance of CHM in rate of conversion to severe cases is proof of its theory of “preventive treatment of disease” from a scientific perspective, as CHM could prevent the disease from becoming severe in the early stage, which also provided robust evidence for advancing the therapeutic window to the early stage of CHM in the treatment of COVID-19. The results of meta-regression analysis were similar to the original results. Besides, sensitivity analyses proved the robustness of our findings. Publication bias was detected in rate of conversion to severe cases and length of hospital stay, and we downgraded the certainty of evidence accordingly.

Regarding the adverse reactions, both evidence from RCTs and OBs showed a relatively high liver dysfunction rate, which is usually higher in control group than in CHM group without statistical difference. Chai et al. (Chai et al., 2020) pointed out that SARS-CoV-2 has been shown to result in an injury to the liver, and cholangiocyte dysfunction and other causes such as drug and systemic inflammatory response rather than hepatocyte damage may induce liver abnormalities. This may explain, to a certain extent, the higher incidence of liver dysfunction than other adverse reactions. For the incidence of diarrhea, as one of key components of LHQW, *Pogostemon cablin* has been shown to ameliorate diarrhea and improve the host-defense of the gastrointestinal tract (Zhou, 2018), which resulted in the significant advantage of LHQW in reducing the incidence rate of diarrhea (Hu K. et al., 2021). On the contrary, JHQG consist of the components such as *Scutellaria baicalensis* and *Anemarrhena asphodeloides* which were bitter in taste and could easily lead to diarrhea. JHQG was used in high doses in studies reporting adverse reactions of diarrhea, the above reasons may lead to diarrhea. In addition, 70.37% (19 cases/27 cases) of the patients with diarrhea in the CHM group had improved after 1–2 days without any special treatment (Duan et al., 2020).

### 4.2 Potential mechanism

CHM was effective against COVID-19 and could be verified by pharmacological mechanism research. Among the CHM treatments of COVID-19, LHQW, QFPD, JHQG, and XFQR were composed of MXSG and other prescriptions. MXSG could down-regulate the secretion level and protein expression level of Interferon α/β (IFN-α/β) in macrophages, inhibit the proliferation of virus (Shi et al., 2017; Zhang S. Y. et al., 2019), improve pulmonary interstitial edema caused by endotoxin (Han, 2020), and play an effective antiviral role. LHQW could block the binding of SARS-CoV-2 with the angiotensin converting enzyme (Niu M. et al., 2020), repress the action of the COVID-19 virus (Zhu et al., 2003), and lessen the content of the virus in cells (Jia et al., 2020; Li et al., 2020). JHQG, which is mainly composed of kaempferol, stigmasterol, has been proved to have antiviral, anti-inflammatory, and immunomodulatory effects based on a modern pharmacological study (Shen et al., 2020), and primarily through phosphoinositide 3-kinase (PI3K)/protein kinase B (AKT), hypoxia-inducible factor-1 (HIF-1), tumor necrosis factor-α (TNF-α), mitogen-activated protein kinase (MAPK), and NF-kB signaling pathways for COVID-19 (Li et al., 2022). Network pharmacology studies have shown that QFPD had 51 potential targets for the treatment of COVID-19 and 26 core components (Zhou et al., 2020). QFPD could straightforwardly follow up on the 3CLpro, block its duplication and angiotensin converting enzyme II (ACE2) to diminish infection passage into cells (Xia et al., 2021). XFQR could remove inflammatory mediators and improve lung function (Zeng and Jin, 2019), and was effective in shortening the length of hospital stay. JYH had advantages in shortening length of hospital stay and accelerating nucleic acid negative conversion, mainly because *Lonicera japonica Thunb.* contained a large amount of microRNA (miRNA) components, which could effectively prevent or inhibit the virus from invading cells (Zhou et al., 2015). As the two phenolic acids with the highest content in JYH, honeysuckle glycoside and chlorogenic acid had a strong reducibility effect and could free radical scavenging (Du et al., 2019). The three components of RDN (*Prunus armeniaca L. var.ansu Maxim.*, *Lonicera japonica Thunb.* and *Carodira jasminoides Ellis*) were proven antiviral properties that inhibit SARS-CoV-2 proliferation *in vitro* and shorten hospital stays (Romero et al., 2006; Efferth et al., 2008; Zhou et al., 2015; Haq et al., 2020). GGQL had a certain intervention effect on rotavirus adsorption to host cells (Yang et al., 2010); JWYPF had a two-way immunomodulatory effect (Li et al., 2011; Ren, 2018; Ma et al., 2020); SAYL was effective in relieving symptoms such as wheezing and coughing in mild patients, and had a favorable impact on inflammatory cytokines and lung function, these 3 CHMs could accelerate virus clearance (Wu et al., 2021).

Andrographolide, the active ingredient of XYP, possessed a high binding affinity to the main protease of SARS-CoV-2, which could activate T lymphocytes, recognize and kill virus-infected host cells or release antiviral cytokines to inhibit virus replication and accelerate time to virus clearance (Peng et al., 2002; Sheeja and Kuttan, 2007a; b). A network pharmacology study found that YDJD inhibited the phosphorylation of p65 by interacting strongly with kappa B (NF-κB) p65 residues to suppress the inflammatory response. QSPDFZ could inhibit the production of inflammatory factors, prevent the virus from attaching to cells, and mobilize the immune function to fight against COVID-19 (Zhong et al., 2018; Shao et al., 2020). RYN contained chemical components such as caffeic acid and flavonoids, which had anti-inflammatory, antiviral, and blood circulation promotion effects. The active ingredients in BZYQ could enhance the phagocytosis of bacteria and viruses by monocyte-macrophage and reticuloendothelial system, thereby enhancing human immune function (Zhan et al., 2017).

The most important thing for mild or moderate patients was to reduce their conversion to severe disease, and the severity of the disease is related to the inflammatory cytokine storm. CHM had an advantage in reducing the rate of severe cases. From the point of view of pharmacological mechanism, there were 246 targets in LHQW, which could act on interleukin 6 (IL-6), TNF-a and other signaling pathways to reduce the inflammatory response in patients (Peng W. et al., 2020). The main targets of HSBD in the treatment of COVID-19 were mitogen-activated protein kinase (MAPK) 3, MAPK 8, TNF, IL-6 and tumor protein p53 (TP53) (Tao et al., 2020), quercetin, ursolic acid and baicalein in HSBD could reduce IL-6 and angiotensin converting enzyme 2 (ACE2) (Niu W. H. et al., 2020; Niu et al., 2021). As the core prescription of QFPD, MXSG could exert an anti-platelet aggregation effect through ephedrine. Chemicals in QFPD could interfere with toll-like receptor 4 (TLR4), and regulate nuclear factor kappa light chain enhancer of activated B cells (NF-kβ) and MAPK signaling pathways to inhibit the release of inflammatory factors (Yang R. et al., 2020). There were 17 chemical components in JHQG, which mainly act on prostaglandin-endoperoxide synthase 2 (PTGS2), TNF-α, NF-κB, IL-6 and other multiple pathways through the Toll-like receptor signaling pathway, and play an anti-COVID-19 effect in a multi-target manner (Peng W. P. et al., 2020). In terms of the composition of QFTXFZ, *Pogostemon cablin* (*Blanco*)*Benth.* oil, *Ephedra sinica Stapf*, *Glycine max (L.) Merr.* and other ingredients had antibacterial and antiviral effects, which could inhibit virus replication and regulate inflammatory responses (Shao et al., 2020). Honokiol extracted in HSY could inhibit transmembrane glycoprotein cluster of differentiation 44 (CD44) and CD54, reduce IL-1β, IL-6 and TNF-α (Zhang X. W. et al., 2019), *Amomum villosum Lour.* could inhibit the binding of S-protein to human ACE2 and reduce virus replication (Niu M. et al., 2020). JYH contained iridoid components such as swertiamarine, which could inhibit the key PI3K/AKT inflammatory pathway and prevent the occurrence of inflammatory factor storm (Jin et al., 2006).

### 4.3 Strengths and limitations

This study had several advantages. Firstly, the study systematically searched all available evidence to evaluate the efficacy and safety of CHM. Secondly, the back-to-back principle for literature screening, data extraction, and bias risk assessment was strictly followed, which ensured the methodological quality. In addition, this systematic review and meta-analysis were conducted and reported following internationally recognized standards to ensure both methodological and reporting quality, and improve research readability. Thirdly, we included all patient-important outcomes considered WHO *“Therapeutics and COVID-19: living guideline”* (Agarwal et al., 2020) and the *“Core Outcome Set for Clinical Trials on Coronavirus Disease 2019”(* *Jin X. et al., 2020* *)*, which could help this review focus on more critical and important outcomes and prove the efficacy of Chinese herbal medicine from a widely recognized perspective. Fourthly, in order to present the efficacy of CHM taking into account the baseline risks, for the dichotomous outcomes, we also calculated the ARD (Agarwal et al., 2017). Moreover, we performed meta-regression analyses, sensitivity analyses and publication bias checks to explore the sources of heterogeneity and test the robustness of the results.

Meanwhile, this study also has some limitations. Firstly, the eligible studies only included general populations without comorbidities, which still lacked evidence for specific populations, such as people with tumors, obesity, chronic kidney disease, etc. Accordingly, the results may be indirect to inform care of patients with comorbidities in practice. Secondly, unlike western medicine, the clinical diagnosis and treatment of traditional Chinese medicine (TCM) are based on comprehensive information such as the patient’s symptoms, tongue image and pulse image, which is the syndrome differentiation, then treatments are conducted according to the identified TCM syndrome type. However, insufficient literature to support our interpretation of the results from the perspective of syndrome differentiation and treatment may limit the thorough assessment of the efficacy and advantages of CHM in the treatment of COVID-19. Thirdly, due to the complicated reality of the epidemic, the included studies are inadequate in study design and reporting, such as no allocation concealment during randomization, no blinding of researchers and patients and inadequate adjustment of confounders etc., these limitations may reduce the reliability of the results. Fourthly, due to the high risk of bias in the CHM primary studies, most results were assessed as low or very low certainty of evidence, indicating that the true effect might or probably be markedly different from the estimated effect.

### 4.4 Implications

CHM had an excellent performance in adapting to the specific symptoms of different mild or moderate patients, reducing the use of western medicine, and in the global promotion and acceptance. All patient recruitment was completed by October 2020 in eligible studies, when none of the patients had been vaccinated against COVID-19, nevertheless, Chinese patent medicines still showed good efficacy. Evidence-based evidence showed that CHM was beneficial in treating mild or moderate patients, but each CHM had specific symptoms to which it was adapted and should be differentiated in clinical use. Our discussion focused on CPM considering their internationally recognized advantages such as relatively mature production process and quality controllable pharmaceutical raw materials. According to the guidelines of traditional Chinese medicine (CAIM, 2020; Wang and Huang, 2020; NHC, 2022), LHQW, JHQG, SFJD and TRQ were used in mild or moderate patients with fever, chills, muscle aches, chest tightness, shortness of breath, sore throat and less phlegm, dry mouth and bitterness. XBJ was effective for patients with fever, palpitations, irritability, and for infection-induced systemic inflammatory syndrome and multiple organ dysfunction syndromes. RDN was suitable for patients with high fever, headache and body pain, cough, and yellow sputum. QFPD is applicable to patients of any type. Published clinical studies have shown that GGQL was suitable for patients with typical gastrointestinal symptoms, such as diarrhea and abdominal pain (Wang L. Q. et al., 2021); XYP may be more advantageous for mild or moderate patients with fever and respiratory symptoms or pulmonary impact characteristics (Zhang X. Y. et al., 2021); when patients were coughing and Expectoration, LHQK could be considered (Sun et al., 2020); RYN was more effective for the symptoms of dry throat, sore throat, cough, fatigue, fever, and chest tightness (Yang M. B. et al., 2020); XST could be used for patients who were chill, fever or no fever, dry cough, dry throat, fatigue, chest tightness and vomiting (Li et al., 2021).

Treated mild or moderate COVID-19 patients with CHM may reduce the use of western medicine. According to the condition of western medicine treatment reported by eligible studies, we found that the use rates of Lopinavir (Xin et al., 2020), Oseltamivir (Xiao M. Z. et al., 2020; Tian et al., 2020; An et al., 2021), Arbidol (Xiao M. Z. et al., 2020; Tian et al., 2020), Ribavirin (Tian et al., 2020), Anti-infective drugs (Xiao M. Z. et al., 2020; An et al., 2021) such as Macrolide (Xiao M. Z. et al., 2020), Antibiotics, Moxifloxacin, Clarithromycin (Tian et al., 2020) showed significant difference between CHM group and control group, and medication rates in CHM group was lower.

The CHM differentiation and treatment method have gradually spread throughout the world, and CHM also has great accessibility in the world. Chinese medicine has spread to more than 100 countries and has developed into an international industry (WHO, 2013). There are about 100,000 Chinese medicine clinics, 300,000 practitioners, and no less than 1,000 CHM education institutions worldwide. As the “Three Medicines and Three Prescriptions” recommended in the *Diagnosis and Treatment Protocol for Novel Coronavirus Pneumonia(* *NHC, 2022* *)* issued by the National Health Commission (China), HSBD was recorded as an emergency registered drug in the United Arab Emirates; XFBD was approved and marketed by the Natural and non-Prescription Health Products Directorate in Canada; JHQG was the first Chinese patent medicine to complete a clinical trial oriented by drug registration overseas; LHQW has obtained marketing licenses in seven countries from 2012 to 2020, such as Canada. In terms of economic cost savings, CHM also possessed certain advantages. The results of a cost-benefit comparative analysis showed that as of 19 February 2020, 45,027 patients had been diagnosed in Wuhan, compared with western medicine using alone, CHM would save an average of 695.28 million dollars (Wang J. et al., 2020). In addition, a cross-sectional study also stated that CHM treatment was significantly negatively associated with non-pharmacologic treatment costs in total cases, moderate cases, and cases without comorbidities (Dong et al., 2021), which also indicated that CHM was beneficial for cost saving.

## 5 Conclusion

The study results showed that CHM had advantages in reducing rate of conversion to severe cases and mortality, shortening time to symptoms resolution, length of hospital stay and time to viral clearance, and increasing rate of nucleic acid conversion and rate of symptoms resolution for mild/moderate patients. No serious adverse events were observed for patients with the treatment of CHMs. However, due to the small sample size and high risk of bias in the randomization process generated and unadjusted confounders, in the context of the continuous variation of the virus, rigorously designed clinical trials and mechanism studies are still warranted to further confirm the effectiveness and safety of CHM in the treatment of COVID-19.

## Data Availability

The original contributions presented in the study are included in the article/Supplementary Material, further inquiries can be directed to the corresponding authors.
