# Supplementary material for: Efficacy and safety of chinese herbal medicine for treating mild or moderate COVID-19: A systematic review and meta-analysis of randomized controlled trials and observational studies
Source: Front Pharmacol. 2022 Sep 7;13:988237. doi: 10.3389/fphar.2022.988237 (PMC9504662; doi:10.3389/fphar.2022.988237)
Supplement: Supplementary file 1 [file DataSheet1.DOCX]

**Role of Chinese Herbal Medicine for Treating Mild or Moderate COVID-19: A Systematic Review and Meta-analysis of Randomized Controlled Trials and Observational Studies**

Hongfei Zhu^1,2^^✝^, Mengting Li^1,2✝^, Chen Tian^1,2^, Honghao Lai^1,2^, Yuqing Zhang^3,4,5,6^, Jiaheng Shi^7,8^, Nannan Shi^7^, Hui Zhao^7^, Kehu Yang^9,10,11^, Hongcai Shang ^12,13^, Xin Sun^14^, Jie Liu^7,15^, Long Ge^1,2,10,11*^, Luqi Huang^7,16*^

1 Department of Social Medicine and Health Management, School of Public Health, Lanzhou University, Lanzhou, China

2 Evidence Based Social Science Research Centre, School of Public Health, Lanzhou University, Lanzhou, China

3 Department of Health Research Methods, Evidence, and Impact, McMaster University, Hamilton, ON, Canada

4 CEBIM (Center for Evidence Based Integrative Medicine)-Clarity Collaboration, Guang’ anmen Hospital, China Academy of Chinese Medical Sciences, Beijing, China

5 Institute of Acupuncture and Moxibustion, China Academy of Chinese Medical Sciences, Beijing, China

6 Nottingham Ningbo GRADE Center, The University of Nottingham Ningbo, China

7 China Center for Evidence Based Traditional Chinese Medicine, China Academy of Chinese Medical Sciences, Beijing, China

8 Department of Emergency, Guang’ anmen Hospital, China Academy of Chinese Medical Sciences, Beijing, China

9 Evidence-Based Medicine Center, School of Basic Medical Sciences, Lanzhou University, Lanzhou, China

10 WHO Collaborating Center for Guideline Implementation and Knowledge Translation, Lanzhou, China

11 Key Laboratory of Evidence Based Medicine and Knowledge Translation of Gansu Province, Lanzhou, China

12 Key Laboratory of Chinese Internal Medicine of Ministry of Education, Beijing, China.

13 Dongzhimen Hospital, Beijing University of Chinese Medicine, Beijing, China.

14 Chinese Evidence-Based Medicine Center, West China Hospital, Sichuan University, Chengdu, China

15 Department of Oncology, Guang’ anmen Hospital, China Academy of Chinese Medical Sciences, Beijing, China

16 National Resource Center for Chinese Materia Medica, China Academy of Chinese Medical Sciences, Beijing 100700, China.

✝ These authors have contributed equally to this work and share first authorship.

*Co-corresponding authors

Luqi Huang, Professor, China Center for Evidence Based Traditional Chinese Medicine/ National Resource Center for Chinese Materia Medica, China Academy of Chinese Medical Sciences, Beijing, China. E-mail address: huangluqi01@126.com

Long Ge, Researcher, Evidence Based Social Science Research Centre, School of Public Health, Lanzhou University. E-mail address: gelong2009@163.com

**Supplementary Materials**

[**Table S1 Detailed search strategy** 4](#_Toc110709061)

[**Table S2 Detailed guidance for assessment of risk of bias** 8](#_Toc110709062)

[**Table S3 Details of baseline risk** 12](#_Toc110709063)

[**Table S4 Characteristics of included studies** 13](#_Toc110709064)

[**Table S5 Studies reporting treatment of COVID-19 patients** 21](#_Toc110709065)

[**Table S6 Risk of bias of included RCTs** 32](#_Toc110709066)

[**Table S7 Risk of bias of included OBs** 34](#_Toc110709067)

[**Table S8 Incidence and difference between groups of adverse reactions** 35](#_Toc110709068)

[**FigureS1.1 Forest plot of rate of conversion to severe cases (RCTs)** 39](#_Toc110709069)

[**FigureS1.2 Forest plot of rate of conversion to severe cases (OBs)** 40](#_Toc110709070)

[**FigureS2.1 Forest plot of time to fever resolution (RCTs)** 41](#_Toc110709071)

[**FigureS2.2 Forest plot of time to cough resolution (RCTs)** 42](#_Toc110709072)

[**FigureS2.3 Forest plot of time to tiredness resolution (RCTs)** 43](#_Toc110709073)

[**FigureS2.4 Forest plot of time to shortness of breath resolution (RCTs)** 44](#_Toc110709074)

[**FigureS2.5 Forest plot of time to total symptoms resolution (RCTs)** 45](#_Toc110709075)

[**FigureS3.1 Forest plot of time to fever resolution (OBs)** 46](#_Toc110709076)

[**FigureS3.2 Forest plot of time to cough resolution (OBs)** 46](#_Toc110709077)

[**FigureS3.3 Forest plot of time to tiredness resolution (OBs)** 48](#_Toc110709078)

[**FigureS3.4 Forest plot of time to expectoration resolution (OBs)** 49](#_Toc110709079)

[**FigureS3.5 Forest plot of time to sore throat resolution (OBs)** 50](#_Toc110709080)

[**FigureS3.6 Forest plot of time to total symptoms resolution (OBs)** 50](#_Toc110709081)

[**FigureS4.1 Forest plot of length of hospital stay (RCTs)** 51](#_Toc110709082)

[**FigureS4.2 Forest plot of length of hospital stay (OBs)** 51](#_Toc110709083)

[**FigureS5.1 Forest plot of time to viral clearance (RCTs)** 52](#_Toc110709084)

[**FigureS5.2 Forest plot of time to viral clearance (OBs)** 53](#_Toc110709085)

[**FigureS6.1 Forest plot of rate of nucleic acid conversion (OBs)** 55](#_Toc110709086)

[**FigureS6.2 Forest plot of rate of nucleic acid conversion (RCTs)** 55](#_Toc110709087)

[**FigureS7.1 Forest plot of rate of mortality (OBs)** 56](#_Toc110709088)

[**FigureS7.2 Forest plot of rate of mortality (RCTs)** 56](#_Toc110709089)

[**FigureS8.1 Forest plot of rate of fever resolution (RCTs)** 57](#_Toc110709090)

[**FigureS8.2 Forest plot of rate of cough resolution (RCTs)** 58](#_Toc110709091)

[**FigureS8.3 Forest plot of rate of tiredness resolution (RCTs)** 59](#_Toc110709092)

[**FigureS8.4 Forest plot of rate of expectoration resolution (RCTs)** 60](#_Toc110709093)

[**FigureS8.5 Forest plot of rate of loss of appetite resolution (RCTs)** 62](#_Toc110709094)

[**FigureS8.6 Forest plot of rate of shortness of breath resolution (RCTs)** 63](#_Toc110709095)

[**FigureS8.7 Forest plot of rate of chest tightness resolution (RCTs)** 63](#_Toc110709096)

[**FigureS8.8 Forest plot of rate of chest tightness and shortness of breath resolution (RCTs)** 63](#_Toc110709097)

[**FigureS8.9 Forest plot of rate of diarrhea resolution (RCTs)** 65](#_Toc110709098)

[**FigureS8.10 Forest plot of rate of CT improvement resolution (RCTs)** 65](#_Toc110709099)

[**FigureS9.1 Forest plot of rate of fever resolution (OBs)** 66](#_Toc110709100)

[**FigureS9.2 Forest plot of rate of cough resolution (OBs)** 67](#_Toc110709101)

[**FigureS9.3 Forest plot of rate of tiredness resolution (OBs)** 68](#_Toc110709102)

[**FigureS9.4 Forest plot of rate of expectoration resolution (OBs)** 69](#_Toc110709103)

[**FigureS9.5 Forest plot of rate of loss of appetite resolution (OBs)** 69](#_Toc110709104)

[**FigureS9.6 Forest plot of rate of shortness of breath resolution (OBs)** 69](#_Toc110709105)

[**FigureS9.7 Forest plot of rate of chest tightness resolution (OBs)** 70](#_Toc110709106)

[**FigureS9.8 Forest plot of rate of diarrhea resolution (OBs)** 71](#_Toc110709107)

[**FigureS9.9 Forest plot of rate of CT improvement (OBs)** 72](#_Toc110709108)

[**FigureS10 Publication bias** 73](#_Toc110709109)

[**10.1 Rate of conversion to severe cases-RCTs** 73](#_Toc110709110)

[**10.2 Rate of conversion to severe cases-OBs** 73](#_Toc110709111)

[**10.3 Length of hospital stay-RCTs** 75](#_Toc110709112)

[**10.4 Length of hospital stay-OBs** 76](#_Toc110709113)

[**10.5 Time to viral clearance-RCTs** 78](#_Toc110709114)

[**Figure S11** **Meta-regression analysis** 80](#_Toc110709115)

[**11.1 Length of hospital stay-RCTs** 80](#_Toc110709116)

[**11.2 Length of hospital stay-OBs** 82](#_Toc110709117)

[**11.3 Time to viral clearance-RCTs** 83](#_Toc110709118)

[**FigureS12 Sensitivity analysis** 85](#_Toc110709119)

[**12.1 Length of hospital stay-RCTs** 85](#_Toc110709120)

[**12.2 Length of hospital stay-OBs** 86](#_Toc110709121)

[**12.3 Time to viral clearance -RCTs** 87](#_Toc110709122)

**Table S1 Detailed search strategy**

| **Databases [Platform]** | **Results** |
| --- | --- |
| CNKI | 1531 |
| WANFANG | 750 |
| CBM | 10,942 |
| VIP | 3,173 |
| PubMed | 72,746 |
| Cochrane Library | 8,901 |
| Embase | 48,654 |
| Web of Science | 65,572 |
| LOVE | 3,492 |
| **subotal** | **215,761** |
| **Duplicate** | **98,970** |
| **Total** | **116,791** |

**CNKI** March 19, 2022

Searched a sub-set of CNKI named *Open Access Online-First Publication Knowledge Service Platform of Fighting against Novel Coronavirus Pneumonia*, which is a special database collected COVID-19 literatures. Therefor, we only searched study design in this database.

Search Strategy:

| **#** | **Searches** | **Results** |
| --- | --- | --- |
| 1 | SU = (“随机” + “对照” + “队列” + “观察” + “观察” + “试验” + “临床”+ “临床回顾” + “病例对照” + “多中心回顾性研究”) | 542 |
| 2 | TKA = (“随机” + “对照” + “队列” + “观察” + “观察” + “试验” + “临床”+ “临床回顾” + “病例对照” + “多中心回顾性研究”) | 1,531 |
| 3 | 1 OR 2 AND Date:2019-* | 1,531 |

**WANFANG** March 19, 2022

Search Strategy:

| **#** | **Searches** | **Results** |
| --- | --- | --- |
| 1 | 题名或关键词:(COVID-19 or COVID19 or COVID-2019 or SARS-CoV-19 or SARS-CoV-2019 or SARS-CoV-2 or SARS2 or 2019-nCoV or 严重急性呼吸综合征冠状病毒2型 or 冠状病毒感染or 武汉冠状病毒 or 武汉海鲜市场肺炎病毒or 冠状病毒2019 or SARS-CoV-19 or SARS-CoV-2019 or 2019新型冠状病毒 or 2019新型冠状病毒感染 or 2019冠状病毒 or 新型冠状病毒-19 or 冠状病毒) | 80,594 |
| 2 | 题名或关键词:(随机 or 对照 or 队列 or 观察 or 疗效 or 试验 or 临床or 临床回顾 or 病例对照 or 多中心回顾性研究) | 6,557,937 |
| 3 | 1 AND 2 AND Date:2019-* | 750 |

**CBM** March 19, 2022

Search Strategy:

| **#** | **Searches** | **Results** |
| --- | --- | --- |
| 1 | "SARS病毒"[不加权:扩展] | 7055 |
| 2 | "COVID-19"[常用字段:智能] OR "COVID19"[常用字段:智能] OR "COVID-2019"[常用字段:智能] OR "SARS-CoV-19"[常用字段:智能] OR "SARS-CoV-2019"[常用字段:智能] OR "SARS-CoV-2"[常用字段:智能] OR "SARS2"[常用字段:智能] OR "2019-nCoV"[常用字段:智能] OR "严重急性呼吸综合征冠状病毒2型"[常用字段:智能] OR "冠状病毒感染"[常用字段:智能] OR "武汉冠状病毒"[常用字段:智能] OR "武汉海鲜市场肺炎病毒"[常用字段:智能] OR "冠状病毒2019"[常用字段:智能] OR "2019新型冠状病毒"[常用字段:智能] OR "2019新型冠状病毒感染"[常用字段:智能] OR "2019冠状病毒"[常用字段:智能] OR "新型冠状病毒-19"[常用字段:智能] OR "冠状病毒"[常用字段:智能] | 35,405 |
| 3 | 1 OR 2 | 35,714 |
| 4 | "随机对照试验"[不加权:扩展] | 499,796 |
| 5 | "病例对照研究"[不加权:扩展] | 1,611,709 |
| 6 | "随机"[常用字段:智能] OR "对照"[常用字段:智能] OR "队列"[常用字段:智能] OR "观察"[常用字段:智能] OR "疗效"[常用字段:智能] OR "试验"[常用字段:智能] OR "临床"[常用字段:智能] OR "疗效"[常用字段:智能] OR "临床回顾"[常用字段:智能] OR "病例对照"[常用字段:智能] OR "多中心回顾性研究"[常用字段:智能] | 6,595,253 |
| 7 | 4 OR 5 OR 6 | 6,675,946 |
| 8 | 3 AND 7 AND 2019-2022[日期] | 10,942 |

VIP March 19, 2022

| 1 | M=("COVID-19" OR "COVID19" OR "COVID-2019" OR "SARS-CoV-19" OR "SARS-CoV-2019" OR "SARS-CoV-2" OR "SARS2" OR "S2019-nCoV" OR "严重急性呼吸综合征冠状病毒2型" OR "冠状病毒感染" OR "武汉冠状病毒" OR "武汉海鲜市场肺炎病毒" OR "冠状病毒2019" OR "新型冠状病毒" OR "2019新型冠状病毒感染" OR "2019冠状病毒" OR "新型冠状病毒-19" OR "冠状病毒") | 34,418 |
| --- | --- | --- |
| 2 | M=("随机" OR “对照”OR "队列" OR "观察" OR "疗效" OR "试验" OR 临床 OR "临床回顾" OR "多中心回顾性研究") | 3,838,482 |
| 3 | 1 AND 2 AND (years: [2019 TO 2022]) | 3,173 |

**PubMed** 2019/01/01-2021/3/19

Search Strategy:

| **#** | **Searches** | **Results** |
| --- | --- | --- |
| 1 | "COVID-19"[Mesh] | 146,853 |
| 2 | "SARS-CoV-2"[Mesh] | 117,877 |
| 3 | coronavirus[Title/Abstract] OR "corona virus"[Title/Abstract] OR coronavirinae[Title/Abstract] OR coronaviridae[Title/Abstract] OR betacoronavirus[Title/Abstract] OR covid19[Title/Abstract] OR "covid 19"[Title/Abstract] OR nCoV[Title/Abstract] OR "CoV 2"[Title/Abstract] OR CoV2[Title/Abstract] OR sarscov2[Title/Abstract] OR 2019nCoV[Title/Abstract] OR "novel CoV"[Title/Abstract] OR "wuhan virus"[Title/Abstract] OR "COVID-19"[Title/Abstract] OR "COVID-2019"[Title/Abstract] OR "SARS-CoV-19"[Title/Abstract] OR "SARS-CoV-2019"[Title/Abstract] OR "SARS-CoV-2"[Title/Abstract] OR "SARS2"[Title/Abstract] OR "2019-nCoV"[Title/Abstract] | 240,408 |
| 4 | 1-3/OR | 246,984 |
| 5 | "Randomized Controlled Trial" [Publication Type] | 563,075 |
| 6 | "Randomized Controlled Trials as Topic"[Mesh] | 157,351 |
| 7 | "Case-Control Studies"[Mesh] | 1,296,884 |
| 8 | "Cohort Studies"[Mesh] | 2,313,454 |
| 9 | "Controlled Before-After Studies"[Mesh] | 690 |
| 10 | "Cross-Sectional Studies"[Mesh] | 415,829 |
| 11 | "Historically Controlled Study"[Mesh] | 220 |
| 12 | "randomized controlled trial"[Title/Abstract] OR "controlled clinical trial"[Title/Abstract] OR "randomized"[Title/Abstract] OR "placebo"[Title/Abstract] OR "randomly"[Title/Abstract] OR "trial"[Title/Abstract] OR "clinical research"[Title/Abstract] OR "clinical observation"[Title/Abstract] OR "control"[Title/Abstract] OR cohort*[Title/Abstract] OR prospective[Title/Abstract] OR longitudinal[Title/Abstract] OR "follow up"[Title/Abstract] OR "case control"[Title/Abstract] OR "case referent"[Title/Abstract] OR "case stud*"[Title/Abstract] OR "case series"[Title/Abstract] OR "cross sectional"[Title/Abstract] | 6,133,486 |
| 13 | 5-12/OR | 7,346,068 |
| 14 | 4 AND 13 | 74,472 |
| 15 | 14 AND ("2019/01/01"[Date - Create] : "2022/3/19"[Date - Create]) | 72,746 |

**Embase** 2019/01/01-2022/03/19

Search Strategy:

| **#** | **Searches** | **Results** |
| --- | --- | --- |
| 1 | 'coronavirus disease 2019'/exp | 197,781 |
| 2 | coronavirus:ab,ti OR 'corona virus':ab,ti OR coronavirinae:ab,ti OR coronaviridae:ab,ti OR betacoronavirus:ab,ti OR covid19:ab,ti OR 'covid 19':ab,ti OR nCoV:ab,ti OR 'CoV 2':ab,ti OR CoV2:ab,ti OR sarscov2:ab,ti OR 2019nCoV:ab,ti OR 'novel CoV':ab,ti OR 'wuhan virus':ab,ti OR 'COVID-19':ab,ti OR 'COVID-2019':ab,ti OR 'SARS-CoV-19':ab,ti OR 'SARS-CoV-2019':ab,ti OR 'SARS-CoV-2':ab,ti OR 'SARS2':ab,ti OR '2019-nCoV':ab,ti | 250,925 |
| 3 | 1 OR 2 | 270,074 |
| 4 | 'randomized controlled trial'/exp | 703,537 |
| 5 | 'randomized controlled trial (topic)'/exp | 222,499 |
| 6 | 'case control study'/exp | 202,934 |
| 7 | 'cohort analysis'/exp | 818,348 |
| 8 | 'cross-sectional study'/exp | 468,911 |
| 9 | 'randomized controlled trial':ab,ti OR 'controlled clinical trial':ab,ti OR 'randomized':ab,ti OR 'placebo':ab,ti OR 'randomly':ab,ti OR 'trial':ab,ti OR 'clinical research':ab,ti OR 'clinical observation':ab,ti OR 'control':ab,ti OR cohort*:ab,ti OR prospective:ab,ti OR longitudinal:ab,ti OR 'follow up':ab,ti OR 'case control':ab,ti OR 'case referent':ab,ti OR 'case stud*':ab,ti OR 'case series':ab,ti OR 'cross sectional' | 8,565,088 |
| 10 | 4-9/OR | 8,858,076 |
| 11 | 10 AND 3 [medline]/lim NOT ([embase classic]/lim AND [medline]/lim) AND [01-01-2019]/sd NOT [20-03-2022]/sd | 48,654 |

**Web of Science** 2019/01/01-2022/03/19

Search Strategy:

| **#** | **Searches** | **Results** |
| --- | --- | --- |
| 1 | TS=(coronavirus OR "corona virus" OR coronavirinae OR coronaviridae OR betacoronavirus OR covid19 OR "covid 19" OR nCoV OR "CoV 2" OR CoV2 OR sarscov2 OR 2019nCoV OR "novel CoV" OR "wuhan virus" OR "COVID-19" OR "COVID-2019" OR "SARS-CoV-19" OR "SARS-CoV-2019" OR "SARS-CoV-2" OR "SARS2" OR "2019-nCoV") | 282,624 |
| 2 | TS=("randomized controlled trial" OR "controlled clinical trial" OR "randomized" OR "placebo" OR "randomly" OR "trial" OR "clinical research" OR "clinical observation" OR "control" OR cohort* OR prospective OR longitudinal OR "follow up" OR "case control" OR "case referent" OR "case stud*" OR "case series" OR "cross sectional") | 9,033,609 |
| 3 | 1 AND 2 AND 2019/01/01-2022/03/19 | 65,572 |

**Cochrane Library** 2019/01/01-2022/03/19

Search Strategy:

| **#** | **Searches** | **Results** |
| --- | --- | --- |
| 1 | MeSH descriptor: [Coronavirus] this term only | 4 |
| 2 | MeSH descriptor: [Coronavirus Infections] this term only | 678 |
| 3 | MeSH descriptor: [Betacoronavirus] this term only | 127 |
| 4 | (coronavirus):ti,ab,kw OR ("corona virus"):ti,ab,kw OR (coronavirinae):ti,ab,kw OR (coronaviridae):ti,ab,kw OR (betacoronavirus):ti,ab,kw OR (covid19):ti,ab,kw OR ("covid 19"):ti,ab,kw OR (nCoV):ti,ab,kw OR ("CoV 2"):ti,ab,kw OR (CoV2):ti,ab,kw OR (sarscov2):ti,ab,kw OR (2019nCoV):ti,ab,kw OR ("novel CoV"):ti,ab,kw OR ("wuhan virus"):ti,ab,kw OR ("COVID-19"):ti,ab,kw OR ("COVID-2019"):ti,ab,kw OR ("SARS-CoV-19"):ti,ab,kw OR ("SARS-CoV-2019"):ti,ab,kw OR ("SARS-CoV-2"):ti,ab,kw OR ("SARS2"):ti,ab,kw OR ("2019-nCoV"):ti,ab,kw | 9,963 |
| 5 | 1-4/OR | 9,963 |
| 6 | MeSH descriptor: [Randomized Controlled Trial] explode all trees | 119 |
| 7 | MeSH descriptor: [Case-Control Studies] explode all trees | 14,490 |
| 8 | MeSH descriptor: [Cohort Studies] explode all trees | 158,377 |
| 9 | MeSH descriptor: [Controlled Before-After Studies] explode all trees | 86 |
| 10 | MeSH descriptor: [Cross-Sectional Studies] explode all trees | 5,023 |
| 11 | MeSH descriptor: [Historically Controlled Study] explode all trees | 22 |
| 12 | ("randomized controlled trial"):ti,ab,kw OR ("controlled clinical trial"):ti,ab,kw OR ("randomized"):ti,ab,kw OR ("placebo"):ti,ab,kw OR ("randomly"):ti,ab,kw OR ("trial"):ti,ab,kw OR ("clinical research"):ti,ab,kw OR ("clinical observation"):ti,ab,kw OR ("control"):ti,ab,kw OR (cohort*):ti,ab,kw OR (prospective):ti,ab,kw OR (longitudinal):ti,ab,kw OR ("follow up"):ti,ab,kw OR ("case control"):ti,ab,kw OR ("case referent"):ti,ab,kw OR ("case stud*"):ti,ab,kw OR ("case series"):ti,ab,kw OR ( "cross sectional"):ti,ab,kw | 1,464,394 |
| 13 | 6-12/OR | 1,465,230 |
| 14 | 5 AND 13 | 8,959 |
| 15 | 14 limited 2019/01/01-2022/03/19 | 8,901 |

**LOVE** 2019.01.01-2022.03.19

Search Strategy:

| **#** | **Searches** | **Results** |
| --- | --- | --- |
| 1 | "randomized controlled trial" OR "controlled clinical trial" OR "randomized" OR "placebo" OR "randomly" OR "trial" OR "clinical research" OR "clinical observation" OR "control" OR cohort* OR prospective OR longitudinal OR "follow up" OR “case control” OR “case referent” OR “case stud*” OR “case series” OR “cross sectional” | 3,492 |

**Table S2 Detailed guidance for assessment of risk of bias**

**Cochrane tool for assessing risk of bias in randomized clinical trials (RoB 2.0)**

| **Bias from the randomization process** | |
| --- | --- |
| Issues to consider:  Random sequence generation  Allocation concealment | |
| **Definitely low risk of bias** | Trials that assign participants to alternative interventions using a randomly generated sequence and maintain allocation concealment.  Examples of methods for developing a randomly generated allocation sequence include a random number generator, random number table, coin tossing, shuffling cards or envelopes, and throwing dice. If a trial is described as 'randomized' without any additional details related to how the allocation sequence was developed, we will assume that the allocation sequence was appropriately developed.  Examples of methods for maintaining allocation concealment include using central allocation via a computer or phone system, pharmacy-controlled allocation, opaque sealed envelopes, and sequentially numbered drug containers.  *Note that an explicit description of random sequence generation is not necessary for a rating of low risk of bias.* |
| **Probably low risk of bias** | Trials in which healthcare providers were blind to the intervention but which provide no information on allocation concealment and in which there are no major baseline imbalances.  *Note that an explicit description of random sequence generation is not necessary for a rating of probably low risk of bias.* |
| **Probably high risk of bias** | Trials in which healthcare providers were not blind to the intervention and which provide no information on allocation concealment.  Trials in which there are substantial baseline differences between trial arms that suggest a problem with the randomization process but there are no other limitations related to randomization. |
| **Definitely high risk of bias** | Trials in which allocation is by judgment of the clinician, by preference of the participant, by availability of the intervention, based on the results of a laboratory test, or other non-random rules (e.g., birthdate, etc.).  Trials in which investigators enrolling participants could possibly foresee the arm to which each subsequent patient would be randomized, such as allocation using an open allocation schedule (e.g. a list of random numbers), assignment envelopes used without appropriate safeguards (e.g. use of unsealed, non-opaque or not sequentially numbered envelopes), alternation between arms, case record number, or any other explicitly unconcealed procedure, rate as high risk. |
| **Bias due to deviations from the intended intervention** | |
| Issues to consider:  Blinding of healthcare providers/clinicians and participants  Imbalances in cointerventions or behaviors | |
| **Definitely low risk of bias** | Therapy trials in which healthcare providers are blind to the intervention administered and in which there are no significant differences in administered co-interventions.  Therapy trials that are described as double or triple blind.  Prophylaxis trials in which participants are blind to the intervention that they have been randomized.  Prophylaxis trials that are described as double or triple blind. |
| **Probably low risk of bias** |  |
| **Probably high risk of bias** | Therapy trials in which healthcare providers are not blind to the intervention administered.  Therapy trials in which healthcare providers are blind to the intervention administered but there are significant differences in administered co-interventions that suggests that blinding may have been compromised.  Therapy trials in which healthcare providers are described as being blind to the intervention but allocation concealment was inadequate.  Prophylaxis trials in which participants are not blind to the intervention that they have been randomized.  Prophylaxis trials in which participants are blind to the intervention to which they have been randomized but there are significant differences in social distancing and risk-taking behaviors that suggest that blinding may have been compromised.  Prophylaxis trials in which healthcare providers are not blind to the intervention and in which healthcare providers were very involved and counselled patients on social distancing, risk-taking behaviors, or testing for COVID-19. |
| **Definitely high risk of bias** | Therapy trials in which healthcare providers are not blind to the intervention and in which there are significant differences in administered co-interventions.  Prophylaxis trials in which participants are not blind to the intervention and in which there are significant differences in social distancing and risk-taking behaviors. |
| **Bias due to missing data** | |
| Issues to consider:  Missing outcome measures  Loss to follow-up | |
| **Definitely low risk of bias** | Trials in which missing outcome data (including outcome data that has been imputed) < 10%.  For in-patient trials, we will assume low risk of bias due to missing data unless otherwise specified. |
| **Probably low risk of bias** | Trials in which missing outcome data (including outcome data that has been imputed) is between 10% to 15% and missing outcome data is unlikely to be related to the true outcome and there is no imbalance in numbers of or reasons for missing data across intervention groups. |
| **Probably high risk of bias** | Trials in which missing outcome data (including outcome data that has been imputed) is between 10% to 15% and missing outcome data is likely to be related to the true outcome or there are imbalances in numbers of or reasons for missing data across intervention groups. |
| **Definitely high risk of bias** | Trials in which missing outcome data (including outcome data that has been imputed) > 15%. |
| **Bias due to measurement of the outcome** | |
| Issues to consider:  Blinding of outcome adjudicators  Objectivity of outcome  *Note that the judgments may differ across outcomes.* | |
| **Definitely low risk of bias** | Trials in which patients are blind to the intervention and in which outcomes are patient-reported.  Trials in which outcomes are measured by a third-party (investigator or clinician) and in which the third-party is blind to the intervention.  Trials in which the outcomes are objective (e.g., mortality, infection with COVID-19 confirmed by a positive RT-PCR swab, mechanical ventilation, admission to hospital, duration of hospital stay, ICU length of stay, ventilator free days, duration of mechanical ventilation, time to clinical improvement if clinical improvement is measured via objective criteria, viral clearance, time to viral clearance).  Trials that are described as double or triple blind. |
| **Probably low risk of bias** |  |
| **Probably high risk of bias** |  |
| **Definitely high risk of bias** | Trials in which patients are not blind and in which outcomes are patient-reported (e.g., time to symptom resolution).  Trials in which outcome adjudicators are not blind and the outcomes are not objective (e.g., adverse effects leading to discontinuation, transfusion-related acute lung injury, transfusion-associated circulatory overload, allergic reactions, infection with suspected/symptomatic COVID-19, venous thromboembolism, time to symptom resolution including fever, time to clinical improvement if the criteria for clinical improvement are not objective). |
| **Bias in selection of the reported results** | |
| Issues to consider:  Selective reporting of timepoints  Selective reporting of outcome measures  *Note that we are only interested in selective reporting for the outcomes for which we are extracting data.*  *Note that the judgments may differ across outcomes.* | |
| **Definitely low risk of bias** | Results for outcomes that were analyzed and reported according to a pre-specified statistical analysis plan or protocol (including the timepoint for the measurement of the outcome). |
| **Probably low risk of bias** | Results for outcomes that were analyzed and reported but that were not prespecified in a statistical analysis plan or protocol but the timepoint at which results are reported is consistent with the timepoint for other outcomes in the trial report or there is little reason to believe the outcome was selectively reported.  Please note that outcomes that were not prespecified in a protocol or statistical analysis plan and that are reported in the trial preprint or publication should be rated at probably low risk of bias unless there are other important reasons to suspect that results for those outcomes were selectively reported (e.g., results are presented at timepoints that don’t match the timepoints reported for other outcomes). |
| **Probably high risk of bias** | Results for outcomes that were analyzed and reported but that were not prespecified in a statistical analysis plan or protocol but the timepoint at which results are reported is not consistent with the timepoint for other outcomes in the trial report or there are other reasons to believe that the outcome is selectively reported. |
| **Definitely high risk of bias** | Results for outcomes that were analyzed and reported for which there are inconsistencies with the statistical analysis plan or protocol. These inconsistencies may include outcome measures of interest or the timepoints for the measurement of outcomes. |
| **Other sources** | |
| Issues to consider:  Other important sources of bias | |
| **Definitely low risk of bias** | Trials appear to be free of other important sources of bias.  For example:  The trial plan or protocol is prospectively registered on the clinical trial registration platform.  The trials are approved by the ethics committee.  The results are written up following the reporting guidelines. |
| **Definitely high risk of bias** | There is at least one important risk of bias.  For example:  No information on the prospective registration and the ethics committee approve.  Incomplete reporting of results or the important information is missing. |

**Table S3 Details of baseline risk**

| Outcomes | | Baseline risk | Origin |
| --- | --- | --- | --- |
| Rate of conversion to severe cases | | 16.52% | Median of the rate of usual supportive care group |
| Rate of nucleic acid conversion | | 16.30% | WHO living guideline |
| Rate of mortality | | 0.40% | WHO living guideline |
| Rate of symptom resolution | Fever | 92.31% | Median of the rate of usual supportive care group |
|  | Cough | 64.50% |  |
|  | Tiredness | 69.32% |  |
|  | Expectoration | 69.75% |  |
|  | Loss of appetite | 79.17% |  |
|  | Shortness of breath | 63.64% |  |
|  | Chest tightness | 28.97% |  |
|  | Chest tightness and shortness of breath | 78.26% |  |
|  | Diarrhea | 75.00% |  |
|  | CT improvement | 65.38% |  |

**Table S4 Characteristics of included studies**

| **Study** | **Study design** | **TCM** | **Journal, JCR** | **Language, registration No.** | **No. of patients** | **Timeline of patient recruitment** | **Recruitment location** | **Mean age (ys)** | **Men (%)** | **Comorbidities** | **Type** |
| --- | --- | --- | --- | --- | --- | --- | --- | --- | --- | --- | --- |
| **Wang Y,2021** | 1 | QFPD | Chinese traditional patent medicine, PKU | Chinese, NR | 140 | February 2, to March 2, 2020 | Xiangyang, Hubei Province, China | 48.7±13.35 | 50.71 | NR | 100% moderate |
| **Zeng XH, 2020** | 2 | QFPD | Medical Journal of West China | Chinese, NR | 228 | December 2019 to March 2020 | Beijing, China | 46.41±5.92 | 54.15 | NR | 100% moderate |
| **Yu HY, 2020** | 2 | QFPD, LHQW, JYBD | Pharmacology and Clinics of Chinese Materia Medica, PKU | Chinese, NR | 214 | February 17, to March 6, 2020 | Wuhan, Hubei Province, China | 47.65±14.89 | 57.48 | CVDs or CAD (0.93%), Diabetes (3.27%), Hypertension (11.21%) | 100% mild and moderate |
| **Zhang LH, 2021** | 2 | QFPD | Phytomedicine, Q1 | English, NR | 8939 | January to May 2020 | 15 hospitals in Hubei Province, China | 55.90 ± 15.60 | 46.6 | Respiratory condition (1.76%), CVDs or CAD (7.76%), Diabetes (15.2%), Hypertension (33.7%) | 4.4% critical |
| **Wang QL, 2021** | 2 | QFPD | Acta Chinese Medicine | Chinese, NR | 72 | July to September 2020 | Urumqi, Xinjiang Uygur Autonomous Region, China | 40.99 | 48.61 | NR | 100% moderate |
| **Xin SY, 2020** | 2 | QFPD | Biomedicine & Pharmacotherapy, Q1 | English, ChiMCTR2000003003/ChiCTR2000029778 | 63 | January 24 to February 15, 2020 | Xiangyang, Hubei Province, China | 50.68±16.01 | 46.03 | DM (11.11%%), HBP (25.4%), CAD (7.94%), Historical Epidemiology (58.73%) | 3.17% mild, 96.83% moderate |
| **Chen RB, 2020** | 2 | QFPD | Journal of Traditional Chinese Medicine, Q4 | Chinese, ChiCTR2100042177 | 45 | January 23, to April 8, 2020 | 14 hospitals in 7 provinces, autonomous regions and municipalities | 44.39±11.81 | 71.11 | Hepatitis B (100%), Comorbidities (other than Hepatitis B) (40%) | 82.22% mild/moderate, 17.78% severe/critical |
| **Sun YN, 2021** | 2 | QFPD | Journal of Traditional Chinese Medicine, PKU | Chinese, NR | 295 | February 5, to March 10, 2020 | Wuhan, Hubei Province, China | 49.20±13.67 | 39.66 | Hypertension (0.68%), Diabetes(0.68%) | 1.36% mild, 97.63%moderate,1.02% untyped |
| **Yu P, 2020** | 1 | LHQW | Chinese Pharmaceutical Journal, PKU | Chinese, NR | 295 | February 17, to March 6, 2020 | Wuhan, Hubei Province, China | 47.76±9.13 | 57.97 | CVDs or CAD (14.24%), Diabetes (9.49%), Hypertension (13.22%) | 9.15% mild, 90.85% moderate |
| **Hu K, 2020** | 1 | LHQW | Phytomedicine, Q1 | English, ChiCTR 2000029434 | 284 | February 2 to 15, 2020 | 23 hospitals in nine provinces of China | 51.1±15.05 | 52.82 | NR | NR |
| **Liu L, 2021** | 2 | LHQW | Medicine, Q3 | English, NR | 108 | February 1, to March 8, 2020 | Wuhan, Hubei Province, China | 57.76±17.13 | 43.52 | CVDs or CAD (11.1%), Diabetes (10.19%), Hypertension (40.74%) | NR |
| **Xiao MZ, 2020** | 1 | LHQW/HXZQ+LHQW | Pharmacological Research, Q1 | English, ChiCTR2000029601 | 182 | February 5 to 10, 2020 | Wuhan, Hubei Province, China | 54.32±13.13 | 56.59 | Coronary artery disease (3.30%), Hypertension (18.13%), Diabetes (5.49%), Hyperlipidemia (6.04%), | NR |
| **Yao KT, 2020** | 2 | LHQW | Chinese Journal of Experimental Traditional Medical Formulae, PKU | Chinese, NR | 42 | January 11 to 30, 2020 | Wuhan, Hubei Province, China | 59.75±13.89 | 66.67 | NR | 100%moderate |
| **Zhang YL, 2020** | 1 | JYH | China Pharmaceuticals | Chinese, NR | 120 | February 6, to March 7 ,2020 | Wuhan, Hubei Province, China | 52.93±13.95 | 60.83 | Diabetes (20.83%), Hypertension (25.83%) | 100% moderate |
| **Hu F, 2020** | 1 | JYH | Traditional Chinese Medicine, PKU | Chinese, NR | 300 | January to March 2020 | Wuhan, Hubei Province, China | 46.77±12.91 | 50 | NR | 100% moderate |
| **Duan C, 2020** | 1 | JHQG | Traditional Chinese Medicine, PKU | Chinese, NR | 123 | February 1 to 5, 2020 | Wuhan, Hubei Province, China | 51.42±13.76 | 50.41 | NR | 100% mild |
| **An XD, 2021** | 1 | JHQG | Frontiers in Medicine, Q1 | English, ChiCTR2000029601 | 123 | NR | Wuhan, Hubei Province, China | 44.81±12.21 | 46.34 | Respiratory condition (0.81%), CVDs or CAD (0.81%), Diabetes (18.7%), Hypertension (1.63%) | 100% mild and moderate |
| **Liu ZL, 2020** | 2 | JHQG | Journal of Traditional Chinese Medicine, Q4 | English, NR | 80 | January 24 to February 17, 2020 | Beijing, China | 51.19 | 46.25 | Respiratory condition (0%), CVDs or CAD (5%), Diabetes (5%), Hypertension (13.75%) | 81.25% moderate, 18.75severe |
| **Zhao C, 2021** | 1 | HSBD | Frontiers in Medicine, Q1 | English, ChiCTR2000029763 | 408 | February 13 to March 7, 2020 | Wuhan, Hubei Province, China | 50.04±15.57 | 49.6 | NR | 100% mild |
| **Liu J, 2021** | 1 | HSBD | Phytomedicine, Q1 | English, ChiCTR2000030288 | 204 | February 27 to March 27, 2020. | Wuhan, Hubei Province, China | - 55.5±10.08 | 37.4 | Respiratory condition (1%), CVDs or CAD (5.1%), Diabetes (13.8%), Hypertension (60%) | 83.6% mild, 16.4% severe |
| **Shi NN, 2020** | 2 | HSBD | Phytomedicine, Q1 | English, ChiCTR2000029400/ChiMCTR2000002940) | 60 | January 30 to March 23, 2020 | Wuhan, Hubei Province, China | 54.77±12.04 | 66.7 | Respiratory condition (8.3%), CVDs or CAD (36.7%),Diabetes (15%) | non-critical |
| **Ma QH, 2021** | 1 | RDN | Journal of Ethnopharmacology, Q1 | English, ChiCTR2000029589 | 50 | February 6 to March 23, 2020 | Lianyungang and Yichang, China | 50.6±15.8 | 56 | hypertension, diabetes mellitus, and CAD (52.0%) | 98% mild, 2% severe |
| **Xu XL, 2021** | 1 | RDN | Annals of Palliative Medicine, Q3 | English, ChiCTR2000029589 | 157 | February 6 to March 23, 2020 | 12 hospitals in ubei, China | 49.8±15.8 | 55.4 | NR | 3.2% mild, 84.1% moderate, 12.7% severe |
| **Xiao Q, 2020** | 2 | SFJD | Journal of Emergency in Traditional Chinese Medicine | Chinese, NR | 200 | January 24 to 30, 2020 | Wuhan, Hubei Province, China | 61.55±8.16 | 65 | NR | 100% mild and moderate |
| **Chen L, 2020b** | 2 | SFJD | Chinese Journal of Experimental Traditional Medical Formulae, PKU | Chinese, NR | 68 | January 27, to March 5, 2020 | Xiangyang, Hubei Province, China | 64.71±10.65 | 42.65 | CVDs or CAD (8.82%), Diabetes (13.24%), Hypertension (30.88%) | 100% moderate |
| **Chen J, 2020** | 2 | SFJD | EXPERT REVIEW OF RESPIRATORY MEDICINE, Q2 | English, NR | 200 | January, 20 to February, 20 2020 | Wuhan, Hubei Province, China | 60.3±6.63 | 65 | NR | 100% moderate |
| **Sun HM, 2020** | 1 | LHQK | Chinese Journal of Experimental Traditional Medical Formulae, PKU | Chinese, NR | 57 | February 25 to March 7, 2020 | Tangshan, Hengshui and Cangzhou, Hebei Province, China | 43.91±13.1 | 49.12 | NR | 100% mild / moderate |
| **Zhang L, 2022** | 1 | LHQK | Evidence-Based Complementary and Alternative Medicine, Q2 | English, ChiCTR2100042069 | 144 | January 14 to March 31, 2020 | Xingtai, Hebei Province and Harbin, Heilongjiang Province, China | 51.19±14.89 | 32.64 | NR | 100% mild/moderate |
| **Wang LQ, 2020** | 1 | GGQL | Modernization of Traditional Chinese Medicine and Materia Medica-World Science and  Technology, PKU | Chinese, NR | 118 | February, 23 to March,15 2020 | Wuhan, Hubei Province, China | 60.20±16.71 | 53.39 | NR | 90.68% mild/moderate, 9.32% severe |
| **Ni L, 2021** | 1 | SHL | FRONTIERS OF MEDICINE, Q2 | English, ChiCTR2000029605 | 235 | February 8, to March 19, 2020 | Wuhan, Hubei Province, China | 53.33±16.41 | 46 | Respiratory condition (3.8%), CVDs or CAD (4.3%), Diabetes (15.17%), Hypertension (25.1%) | 1.3% mild, 80.4% moderate, 18.3% severe |
| **Zhang XY, 2021** | 1 | XYP | Phytotherapy Research, Q1 | English, NCT04295551 | 130 | January 27, to February 20, 2020 | 5 hospitals in Jiangxi Province | 46.28±13.93 | 46.15 | Diabetes (7.69%), Hypertension (16.15%) | 100% mild/moderate |
| **Sun SQ, 2021** | 1 | LS | Chinese traditional patent medicine, PKU | Chinese, CHiCTR2000030469 | 80 | February 19, to March 11, 2020 | Wuhan, Hubei Province, China | 61.68±11.53 | 42.5 | NR | 93.75% moderate, 6.25% severe |
| **Yan YS, 2021** | 1 | YHQF | Traditional Chinese Medicine, PKU | Chinese, NR | 40 | February 5, to 20, 2020 | Changsha, Hunan Province, China | 36.73±8.88 | 52.5 | NR | 100% moderate |
| **Wang JB, 2020** | 1 | KG-1 | Chinese Journal of Integrative Medicine, Q3 | English, NCT 04251871 | 47 | January 22, to February 25, 2020 | Beijing, China | 49.05±16.04 | 55.32 | Chronic diseases (38.30%) | NR |
| **Zeng CC, 2021** | 1 | MWD | Integrative Medicine Research, Q3 | English, ChiCTR2000030759 | 59 | February to May, 2020 | Wenzhou, Zhejiang Province, China | 51.98±14.07 | 67.8 | NR | NR |
| **He Q, 2021** | 1 | BZYQ | Journal of Emergency in Traditional Chinese Medicine | Chinese, NR | 71 | March, 2020 | Wuhan, Hubei Province, China | NR | NR | NR | 100% mild |
| **Li ZJ, 2021** | 1 | XSTXW | Shanghai Journal of Acupuncture and Moxibustion | Chinese, NR | 60 | January to April 2020 | Shenzhen, Guangdong Province, China | 40.5±2.58 | 50 | NR | 100% moderate |
| **Wu R, 2021** | 1 | SAYL | Clinical Journal of Traditional Chinese Medicine | Chinese, NR | 60 | February 8, to February 21, 2020 | Fuyang, Anhui Province, China | 44.1±26.08 | 50 | NR | 100% mild and moderate |
| **Jin W, 2020** | 1 | FFYC+QQJD+Other | Pharmacology and Clinics of Chinese Materia Medica, PKU | Chinese, ChiCTR2000029558 | 38 | January 25, to February 8, 2020 | 6 hospitals in Sichuan Province, China | 42.39±12.18 | 63.16 | NR | 100% moderate |
| **Wang YL, 2020** | 1 | QRKD; LXJD | Hebei Journal of Traditional Chinese Medicine | Chinese, NR | 22;16 | January 21 to April 12, 2020 | Shijiazhuang, Hebei Province, China | 42.58±16.01; 33.22±15.25 | 50; 56.25 | NR | 57.89% moderate; 42.11% asymptomatic infection |
| **AI XY, 2020** | 1 | FY 1; FY recovery recipe | Guangdong Medical Journal | Chinese, NR | 67 | 23 January to 17 March 2020 | Guangzhou, Guangdong Province, China | 50.92±15.57 | 59.7 | Hypertension (8.96%), hypertension with diabetes (2.99%), hepatitis C (1.49%), hepatitis B (1.49%), fatty liver (1.49%) | 100% moderate |
| **Lin FF, 2020** | 1 | XFQR | Zhejiang Journal of Integrated Traditional Chinese and Western Medicine | Chinese, NR | 82 | January 23 to February 13, 2020 | Wenzhou, Zhejiang Province, China | 44.91±12.19 | 46.34 | NR | 100% moderate |
| **Fu XX, 2020** | 1 | TJQW | Chinese Journal of Experimental Traditional Medical Formulae, PKU | Chinese, NR | 65 | January 20 to February 23, 2020 | Guangzhou, Guangdong Province, China | 43.47±6.75 | 55.38 | Hypertension (7.69%), CAD (7.69%), diabetes (4.62%), chronic hepatitis (1.54%) | 100% mild and moderate |
| **AI XY, 2020** | 1 | TJQW | China Tropical Medicine | Chinese, NR | 98 | January 23 to March 3, 2020 | Guangzhou, Guangdong Province, China | 44.84±15.31 | 41.84 | NR | 14.86%mild,74.49% moderate, 10.65%severe |
| **Ding XJ, 2020** | 1 | QFTXFZ | Herald of Medicine, PKU | Chinese, NR | 100 | NR | Wuhan, Hubei Province, China | 59.62±13.33 | 78 | Underlying disease (39%) | 21% mild,70% moderate,9% severe |
| **Qiu M, 2020** | 1 | MXXF | Journal of Emergency in Traditional Chinese Medicine | Chinese, NR | 50 | February 7 to February 17, 2020 | Chongqing, China | 52.34±16.45 | 54 | NR | 100% moderate |
| **Xiong WZ, 2020** | 1 | XFBD | Integrative Medicine Research, Q3 | English, ChiCTR2000034795 | 42 | January 30 to February 10, 2020 | Wuhan, Hubei Province, China | 59.62±13.33 | NR | NR | 100% mild to severe |
| **Zhang CT, 2020** | 1 | JWDY | Pharmacology and Clinics of Chinese Materia, PKU | Chinese, NR | 45 | January 31, to March 3, 2020 | Wuhan, Hubei Province, China | 54.67±4.07 | 42.22 | Hypertension (55.56%),CAD (15.56%), Diabetes (20.00%) | 100%moderate |
| **Ping XH, 2021** | 1 | JWYPF | The Medical Forum | Chinese, NR | 54 | January to March 2020 | Jiujiang, Jiangxi Province, China | 40.96 | 48.15 | NR | 100% mild and moderate |
| **Ai ZZ, 2020** | 2 | QFDYG | Frontiers in Pharmacology, Q1 | English, NR | 84 | January 27 to March 12, 2020 | Wuhan, Hubei Province, China | 52.89±6.32 | 59.52 | Diabetes(25%),Hypertension (25%), Hyperlipemia (15.48%),Coronary disease (9.52%),Chronic hepatitis B (57.14%) | 80.95%mild,19.05%severe |
| **Zhang CY, 2020** | 2 | XBJ | Chin J Hosp Pharm, PKU | Chinese, NR | 44 | January 21 to February 24, 2020 | Wuhan, Hubei Province, China | 47.5±14.79 | 50 | NR | 100% moderate |
| **Yang MB, 2020** | 2 | RYN | Chinese Journal of Experimental Traditional Medical Formulae, PKU | Chinese, NR | 49 | January 21, to March 2, 2020 | Xian, Shanxi Province; Hubei,Province; Yanan,Shanxi Province China | 48.86±15.28 | 51.02 | NR | 100% moderate |
| **Feng Y, 2021** | 2 | YDJD | Phytomedicine, Q1 | English, NR | 194 | January 29, to July 23, 2020 | Beijing, China | 46±13.64 | 54.64 | NR | 11.34% mild, 80.93% moderate, 4.64% severe, 3.09% cirtical |
| **Zhang N, 2020** | 2 | MXSG | Journal of Frontiers of Medicine | Chinese, NR | 120 | February 1 to March 5, 2020 | Chongqing, China | 51.08±12.77 | 51.67 | hypertension (15.83%), CAD (6.67%), diabetes (13.33%), Cerebral infa1ion (5%) | 100% moderate |
| **Lan J, 2020** | 2 | QSPDFZ | Chinese Journal of Integrated Traditional and Western Medicine in Intensive and Critical Care, PKU | Chinese, NR | 85 | February 1 to 20, 2020 | Shiyan, Hubei Province, China | 42.73±13.79 | 75.29 | Basic illness (17.65%) | 4.71% mild, 95.29% moderate |
| **Chen L, 2020a** | 2 | GLXD | Chinese Journal of Experimental Traditional Medical Formulae, PKU | Chinese, NR | 230 | January 25, to March 18, 2020 | Wuhan, Hubei Province, China | 61.60±14.9 | 44.35 | Hypertension (32.17%), CAD (9.57%), diabetes (14.78%), Hyperlipidemia (0.87%), chronic gastritis (1.74%), gout (1.30%) Cerebral infa1ion (3.92%) | 100% moderate |
| **Tian JX, 2020** | 3 | HSY | Pharmacological Research, Q1 | English, ChiCTR2000029601 | 721 | Before March 10, 2020 | 17 quarantine stations in Wuchang District, Wuhan, Hubei Province, China | 48.39±13.72 | 53.38 | Hypertension (16.9%), CAD (4.6%), Diabetes (7.1%), Bronchial asthma (3.3%), Chronic obstructive pulmonary disease (1.2%), Hyperlipidemia (6.7%), Fatty liver (7.9%) | 100% mild/moderate |
| **Zhang X, 2021** | 3 | TRQ | Journal of Integrative Medicine, Q2 | English, ChiCTR2000033320 | 82 | January 26 to April 15, 2020 | Shanghai, China | 40.04±22.19 | 41.46 | Underlying diseases (28.05%) | 86.59%moderate , 13.41%mild |
| **Li HL, 2020** | 2 | self-made formula | World Latest Medicine Information | Chinese, NR | 191 | February to March 2020 | Wuhan, Hubei Province, China | 46.92±6.77 | 49.74% | NR | 100%moderate |

1: RCT; 2: historical control study; 3: control study

**Table S5 Studies reporting treatment of COVID-19 patients**

| **Study** | **Study design** | **Dose and duration** | **TCM components** | **Conventional treatment** |
| --- | --- | --- | --- | --- |
| **Wang Y,2021** | 1 | QFPD (100 mL, 2 times/day) | *Herba Ephedrae*, 9g  *Radixet Rhizoma Glycyrrhizae Praeparata cum Melle*, 6g  *Semen Armeniacae Amarum*, 9g  *Ramulus Cinnamomi,* 9g  *Rhizoma Alismatis*, 9g  *Polyporus*, 9 g  *Rhizoma Atractylodis Macrocephalae*, 9g  *Poria*, 15g  *Radix Bupleuri*, 16g  *Radix Scutellariae*, 6g  *Rhizoma Pinelliae Praeparatum*, 9g  *Rhizoma Zingiberis Recens*, 9g  *Radix et Rhizoma Asteris*, 9g  *Flos Farfarae*, 9g  *Rhizoma Belamcandae*, 9g  *Radix et Rhizoma Asari*, 6g  *Rhizoma Dioscoreae*, 12g  *Fructus Aurantii Immaturus*, 6g  *Pericarpium Citri Reticulatae*, 6g  *Herba Agastachis*, 9g. | The control group was given routine treatment, including symptomatic treatment, nutritional support, anti-virus, bacteria, infection, etc. Moxifloxacin hydrochloride tablets (0.4 g per tablet) 1 tablet/time, 1 time/d; Arbidol hydrochloride dispersible tablets (0.1 g per tablet) 2 tablets/time, 3 times/d. Continuous treatment for 10 days |
| **Zeng XH, 2020** | 2 | QFPD |  | Lopinavir antiviral therapy, nasal cannula, oxygen oxygen therapy by face mask, supplemented by psychological intervention health education, etc. |
| **Yu HY, 2020** | 2 | QFPD 200 ml/bag, 2 times/day; LHQW 4 capsules/time, 3 times/day (0.35 g/capsule); JYBD 3 bags, 10 g/bag, 3 times/day |  | Arbidol Hydrochloride Tablets 200 mg, 3 times/day (100 mg/tablet) |
| **Zhang LH, 2021** | 2 | QFPD |  | those not receiving QPT |
| **Wang QL, 2021** | 2 | QFPD: 1 dose/day, 2 times a day |  | Western medicine treatment, including effective oxygen therapy, antiviral therapy (Arbidol 200 mg, 3 times a day, the course of treatment should not exceed 10 days), subcutaneous injection of thymosin and other measures. |
| **Xin SY, 2020** | 2 | QFPD (200 mL), comprising two consecutive courses, each course lasting 3 days, without a pause between the courses |  | NHC-China Guideline (6th Edition): Throughout the hospitalization period, including effective oxygen therapy measures, antipyretic measures, rehydration, nutritional sup- port, antiviral treatment, combined with antibiotic treatment in case of bacterial infection, and corticosteroids used only in case of inflamma- tion caused by a cytokine storm. |
| **Chen RB, 2020** | 2 | QFPD: 1 dose/day, 2 times/day, 3 doses/course |  | Referring to the NHC-China guidelines (version 3, 4, 5, 6)were tried for routine treatment, including antiviral, antibacterial drug treatment, and oxygen inhalation and other supportive treatments. |
| **Sun YN, 2021** | 2 | QFPD: 200ml, 2times/a day |  | NHC-China Guideline(version 6.0): |
| **Yu P, 2020** | 1 | LHQW: 6g, 3 times/day | *Forsythia suspensa （Thunb.） Vahl*  *Ephedra sinica Stapf*  *Lonicera japonica Thunb.*  *Prunus armeniaca L.*  *Gypsum Fibrosum*  *Isatis indigotica Fort.*  *Dryopteris crassirhizoma Nakai*  *Houttuynia cordata Thunb.*  *Pogostemon cablin（Blanco）Benth.*  *Rheum palmatum L.*  *Rhodiola crenulata （Hook. f. et Thoms. ）H. Ohba*  *Mentha haplocalyx Briq.*  *Glycyrrhiza uralensis Fisch.* | Arbidol hydrochloride dispersible tablets, 0.2g each time, 3 times/d; Moxifloxacin hydrochloride tablets, 0.4g each time, 1 time/d; Ambroxol hydrochloride tablets, 30mg each time, 3 times/d |
| **Hu K, 2020** | 1 | LHQW |  | NHC-China Guideline (Trial version 7.0) |
| **Liu L, 2021** | 2 | LHQW: 1400mg, per 8 hour |  | 200 mg arbidol per 8 hour, The administration period is about 5 to 21days until coronavirus is detected negative by RT-PCR for 3 times. |
| **Xiao MZ, 2020** | 1 | HXZQ: 2.6 g in each bag, 2 times/day LHQW: 6 g per bag, 3 times/day |  | NHC-China Guideline (version 7.0) |
| **Yao KT, 2020** | 2 | LHQW: 1 bag/time, 3 times/day |  | NHC-China guideline |
| **Zhang YL, 2020** | 1 | JYH: 60mL/time,3 times/day | NR | Lopinavir-ritonavir tablets 2 tablets each time, 2 times a day; intramuscular injection of alpha-interferon, 5 million U/time, adding 2 mL of sterile water for injection, 2 times a day; symptomatic and supportive treatment. |
| **Hu F, 2020** | 1 | JYH: 60mL,3 times/day |  | Lopinavir and ritonavir tablets (300 mg per tablet) orally, 2 tablets each time, 2 times a day; recombinant human interferon α2a for injection (injection, 5 million IU each, add 1 ml of sterile water for injection, subcutaneously) Or intramuscular injection, twice a day; symptomatic and supportive treatment, condition monitoring |
| **Duan C, 2020** | 1 | JHQG: 5g/bag, 2 bags/time, 3 times /day | *Lonicera japonica Thunb*  *Gypsum Fibrosum*  *Ephedra sinica Stapf*  *Prunus armeniaca L.*  *Scutellaria baicalensis Georgi*  *Forsythia suspensa （Thunb.） Vahl*  *Fritillaria thunbergii Miq.*  *Anemarrhena asphodeloides Bge.*  *Arctium lappa L.*  *Artemisia annua L.*  *Mentha haplocalyx Briq.*  *Glycyrrhiza uralensis Fisch.* | NHC-China guidelines (version 5.0) |
| **An XD, 2021** | 1 | JHQG: 1 bag/time, TID |  | NHC-China guidelines (version 7.0) |
| **Liu ZL, 2020** | 2 | JHQG: 1 sachet/day, 2 times/day |  | oxygen inhalation, and symptomatic and supportive treatment |
| **Zhao C, 2021** | 1 | HSBD: 20g/bag, 2 times/day | *Ephedra sinica Stapf*, 6g  *Prunus armeniaca L.var.ansu Maxim.*, 9g  *Gypsum Fibrosum,* 15g  *Glycyrrhiza uralensis Fisch.*, 3g  *Pogostemon cablin（Blanco）Benth.*, 10g  *Magnolia officinalis Rehd.et Wils.*, 10g  *Atractylodes lancea（Thunb.）DC.*, 15g  *Amomum tsao-ko Crevost et Lemaire*, 10g  *Pinellia ternate (Thunb.） Breit.*, 9g  *Poria cocos（Schw.）Wolf*, 15g  *Rheum palmatum L.*, 5g  *Astragalus membranaceus（Fisch.） Bge.var.mongholicus（Bge.）Hsiao*, 10g  *Descurainia sophia（L.）Webb. ex Prantl.*, 10g  *Paeonia lactiflora Pall.*, 10g | bed rest, sufficient food and water intake, frequent monitoring of vital signs, and bedside oxygen therapy if necessary. As there was no evidence at the time of this trial that existing antiviral drugs were effective against COVID-19, Arbidol hydrochloride, as acompassionate therapy, could be given to patients under research physicians’ discretion. |
| **Liu J, 2021** | 1 | HSBD: 10 g, twice daily |  | The NHC-NATCM-China guidelines (version 6.0, published on February 18, 2020) |
| **Shi NN, 2020** | 2 | HSBD: 137 g twice daily; SM: 60 mL once daily; XYP: 100 mg twice daily; XBJ: 100 ml twice daily |  | Lopinavir-Ritonavir (500 mg twice daily, orally), anti- biotics (such as cefoperazone, 2 g twice daily, intravenous injection; moxifloxacin hydrochloride tablets, 0.4 g once daily, orally), cortico- steroids (such as methylprednisolone, 40 mg once daily, intravenous injection; prednisone, 30 mg once daily, orally), antiviruses (such as arbidol capsule, 0.2 g three times daily, orally) |
| **Ma QH, 2021** | 1 | 20 mL of RDN injection was diluted with 250 mL of saline, once a day | RDN: *Lonicera japonica Thunb.,*  *Gardenia jasminoides Ellis,*  *Artemisia annua L.* | NHC-China guidelines (Trial version 5.0) |
| **Xu XL, 2021** | 1 | 20 mL of RDN injection was diluted with 250 mL of saline, once a day |  | NHC-China guidelines (Trial version 5.0) |
| **Xiao Q, 2020** | 2 | SFJD: 4 capsules/time, 3 times/day | *Polygonum cuspidatum Sieb. et Zucc.*  *Forsythia suspensa （Thunb.） Vahl*  *Isatis indigotica Fort.*  *Patrinia scabiosaefolia*  *Verbena officinalis L.*  *Bupleurum chinense DC.*  *Phragmites communis Trin.*  *Glycyrrhiza uralensis Fisch.* | Arbidol tablets (0.1 g each) are taken orally, 0.2 g each time, 3 times a day. 2 weeks of medication |
| **Chen L, 2020b** | 2 | SFJD: 2.08 g,3 times/day |  | Routine treatment is bed rest, supportive treatment, and catheter oxygen inhalation when oxygen saturation is low; physical cooling for low fever, oral ibuprofen suspension for high fever (≥38.3 ℃) to assist in antipyretic; severe sputum Oral expectorant with ambroxol hydrochloride tablets (0.6 g/time, 3 times/d); patients with infection were given moxifloxacin hydrochloride tablets for oral anti-infection (0.4 g/time, 1 time/d); at the same time, viral drug hydrochloric acid was given Arbidol capsules orally (0.2 g/time, 3 times/d) |
| **Chen J, 2020** | 2 | SFJD: 4 capsules (0.52 g/capsule) 3 times/day |  | Arbidol Hydrochloride Capsules orally, 2 capsules (0.1 g/ capsule) 3 times a day for 2 weeks. |
| **Sun HM, 2020** | 1 | LHQK: 1 bag/time, 3 times/day | LHQK: *Ephedra sinica Stapf*  *Morus alba L.*  *Gypsum Fibrosum*  *Scutellaria baicalensis Georgi*  *Prunus armeniaca L.*  *Forsythia suspensa （Thunb.） Vahl*  *Fritillaria thunbergii Miq.*  *Peucedanum praeruptorum Dunn*  *Arctium lappa L.*  *Lonicera japonica Thunb*  *Rheum palmatum L.*  *Citrus reticulata Blanco* | NHC-China guidelines (Trial version 6.0/7.0) |
| **Zhang L, 2022** | 1 | LHQK: 4 tablets/time, 3 times/day |  | NHC guideline (8th) |
| **Ping XH, 2021** | 1 | 1 dose/day, 2 times a day | JWYPF: *Astragalus membranaceus（Fisch.） Bge.var.mongholicus（Bge.）Hsiao 30g Atractylodes macrocephala Koidz.* 12g  *Saposhnikovia divaricata （Turcz.）Schischk.*  10g  *Poria cocos（Schw.）Wolf* 10g  *Atractylodes lancea（Thunb.）DC.* 10g  *Pogostemon cablin（Blanco）Benth.* 10g  *Perilla frutescens（L.）Britt.* 10g  *Pinellia ternata（Thunb.） Breit.* 8g  *Amomum villosum Lour.* 6g  *Zingiber officinale Rosc.* 6 g | Lopinavir-ritonavir tablets, 400 mg/100 mg, orally, twice a day, for 10 days + alpha-interferon, twice a day |
| **Wang LQ, 2020** | 1 | GGQL: 1 bag/time, 3 times a day | GGQL: *Pueraria lobata（Willd.）Ohwi*  *Scutellaria baicalensis Georgi*  *Coptis chinensis Franch.*  *Glycyrrhiza uralensis Fisch.* | NHC-China guidelines (version 6.0 and 7) |
| **Ni L, 2021** | 1 | SHL: 20 mL/ 40 mL/ 60 mL, three times daily | NR | All patients received standard care which consisted of supportive treatments, including supplemental oxygen therapy, daily symptom and vital sign monitoring, clinical laboratory testing, correction of water, electrolyte and acid base imbalances, and administration of antiviral agents and antibiotic agents if bacterial infection was found. The antiviral agents used in the standard care included lopinavir/ritonavir, ganciclovir, arbidol hydrochloride, oseltamivir phosphate, ribavirin, entecavir, and interferon. The antibiotics used in the standard care included cephalosporin, moxifloxacin, lavoofloxacin, and azithromycin. Standard care was used according to NHC-China guidelines (version 5.0) |
| **Zhang XY, 2021** | 1 | XYP: 10 mg/kg, once/day, with a maximum daily dosage not to exceed 500 mg | NR | NHC-China guidelines (version 5.0) |
| **Sun SQ, 2021** | 1 | LS: 10 capsules/time, 3 times/day | NR | NHC-guideline (Trial Version 6) |
| **Yan YS, 2021** | 1 | YHQF: 3 capsules/time (0.15 g/capsule),3 times/day | NR | NHC-China guidelines (version 5.0) |
| **Wang JB, 2020** | 1 | KG-1: 19.4 g/time, 2 times/day | *Lonicera japonica Thunb.* 30g  *Forsythia suspensa (Thunb.) Vahl* 30g  *Morus alba L.* 15g  *Chrysanthemum morifolium Ramat. 1*0g  *Coix lacryma-jobi L. var. mayuen 3*0g  *Fritillaria thunbergia Miq.* 15g  *Prunus armeniaca L. var. ansu Maxim.* 9g. | NHC-China guidelines (version 3.0) |
| **Zeng CC, 2021** | 1 | MWD: 200ml/time, 2 times/day | *Ephedra sinica Stapf* 10g  *Prunus armeniaca L.* 10g  *Gypsum Fibrosum* 45g  *Phragmites communis Trin.* 30g  *Prunus persica（L.）Batsch* 20g  *Benincasa hispida（Thunb.）Cogn.* 20g  *Trichosanthes kirilowii Maxim.* 30g  *Citrus reticulata Blanco* 12g  *Zingiber officinale Rosc. Pinellia ternata（Thunb.） Breit.* 12g  *Bambusa tuldoides Munro* 12g  *Descurainia sophia（L.）Webb. ex Prantl.* 30g  *Acorus tatarinowii Schott* 15g  *Glycyrrhiza uralensis Fisch.* 10g  *Curcuma phaeocaulis VaL.* 5g | Staying in bed, oxygen therapy provided by a nasal cannula, broad-spectrum antibiotics and antivirals. |
| **He Q, 2021** | 1 | BZYQ: 1 dose/day, 2 times a day | *Astragalus membranaceus（Fisch.） Bge.var.mongholicus（Bge.）Hsiao* 10g  *Panax ginseng C. A. Mey.* 3g  *Glycyrrhiza uralensis Fisch.* 5g  *Atractylodes macrocephala Koidz. 3g*  *Citrus reticulata Blanco* 3g  *Angelica sinensis（Oliv.）Diels*  3g  *Cimicifuga heracleifolia Kom.* 3g  *Bupleurum chinense DC.* 3g | Arbidol 200 mg, 3 times a day for 5 consecutive days |
| **Li ZJ, 2021** | 1 | XSTXW: 70 mg/bottle, 3 bottle/time, 1 time a day | gv20、gv14、gv9、gv4、gv3、b24、b17 | Take comprehensive treatment of western medicine. ①Isolation treatment. ② symptomatic treatment. Rest, oxygen therapy, cooling, and cough and expectorant drugs should be given to those with severe cough and phlegm. ③ antiviral treatment. Alpha-interferon aerosol inhalation, 5 million U each time, 2 times a day; Lopinavir, 200 mg each time, 2 capsules each time, 2 times a day; or ritonavir, 50 mg each time, 2 capsules each time, 2 times a day. ④ Use antibacterial therapy as appropriate. A total of 20 days of treatment. |
| **Wu R, 2021** | 1 | SAYL: 125mL/time, 2 times/day | NR | Oxygen inhalation, Western medicine antiviral and symptomatic treatment were adopted, and the decoction of "Qingfei Paidu Decoction" was taken orally, 1 dose and 2 times a day.。 |
| **Jin W, 2020** | 1 | FFCH/QQJD: 15g/time, 4 times a day | FFCH:  *Lonicera japonica Thunb.*  *Bupleurum chinense DC.*  *Phragmites communis Trin.*  *Eriobotrya japonica（Thunb.）Lindl.*  *Mentha canadensis Linnaeus*  *Pogostemon cablin（Blanco）Benth.*  *Schizonepeta tenuifolia Briq.*  QQJD:  *Lonicera japonica Thunb.*  *Forsythia suspensa （Thunb.） Vahl*  *Pueraria thomsonii Benth.*  *Angelica dahurica（Fisch.ex Hoffm.）Benth.et Hook.f.*  *Artemisia annua L.*  *Bupleurum chinense DC.*  *Paris polyphylla Smith var.yunnanensis(Franch.)Hand.-Mazz.*  *Isatis indigotica Fort.*  *Iris tectorum Maxim.*  *Taraxacum mongolicum Hand. -Mazz.*  *Isatis indigotica Fort.*  *Pogostemon cablin（Blanco）Benth.*  *Perilla frutescens（L.）Britt.*  *Mentha haplocalyx Briq.* | NHC-China guidelines (version 5.0)  (Trail version 4) |
| **Wang YL, 2020** | 1 | QRKD: 20 mL, 3 times daily  LXJD: 20 mL, 3 times daily | NR | Recombinant human interferon α2b injection 5 million U plus 2 mL of sterile water for injection, aerosol inhalation, 2 times a day. Arbidol hydrochloride tablets 0.2 g, orally 3 times a day. |
| **AI XY, 2020** | 1 | 1 dose/day, 2 times/day | *Bupleurum chinense DC.*  *Scutellaria baicalensis Georgi*  *Pinellia ternata (Thunb.) Breit.*  *Codonopsis pilosula (Franch.) Nannf.*  *Trichosanthes kirilowii Maxim.*  *Areca catechu L.*  *Amomum tsao-ko CrevostetLemaire*  *Magnolia officinalis Rehd. et Wils.*  *Anemarrhena asphodeloides Bge*  *Paeonia lactiflora Pall.*  *Glycyrrhiza uralensis Fisch.*  *Citrus reticulata Blanco*  *Polygonum cuspidatum Sieb. et Zucc.* | Oxygen therapy, antiviral, anti-infective, cough and phlegm relief and other supportive therapy, no medication during the recovery period |
| **Fu XX, 2020** | 1 | TJQW: 2 times/day |  | Oral antiviral drug Arbidol tablets, 0.2 g/time, 3 times a day; Moxifloxacin tablets, 0.4 g/time, 1 time a day; Ambroxol tablets, 30 mg/time, 3 times a day Second-rate. |
| **Ai ZZ, 2020** | 2 | QFDYG: 18g, 3 times a day | *Bupleurum chinense DC.*  *Scutellaria baicalensis Georgi*  *Pinellia ternata (Thunb.) Breit.*  *Codonopsis pilosula (Franch.) Nannf.*  *Trichosanthes kirilowii Maxim.*  *Areca catechu L.*  *Amomum tsao-ko CrevostetLemaire*  *Magnolia officinalis Rehd. et Wils.*  *Anemarrhena asphodeloides Bge*  *Paeonia lactiflora Pall.*  *Glycyrrhiza uralensis Fisch.*  *Citrus reticulata Blanco*  *Polygonum cuspidatum Sieb. et Zucc.* | NHC-China Guideline (4th edition, 5th edition and 6th edition): the antivirals oral alpha interferon inhalation, oseltamivir, arbidol ,and other treatments or interventions based ondisease progression |
| **Lin FF, 2020** | 1 | XFQR: 1 dose/day, 2 times/day | *Ephedra sinica Stapf* 9g  *Prunus armeniaca L.* 12g  *Gypsum Fibrosum* 30g  *Glycyrrhiza uralensis Fisch.* 6g  *Prunus persica（L.）Batsch* 12g  *Benincasa hispida（Thunb.）Cogn.* 30g  *Phragmites communis Trin.* 30g  *Coix lacryma-jobi L.var.ma-yuen(Roman.) Stapf* 30g  *Platycodon grandiflorum （Jacq.）A.DC.* 9g  *Zingiber officinale Rosc. Pinellia ternata（Thunb.） Breit.* 12g  *Allium chinense G. Don* 12g  *Amomum tsao-ko Crevost et Lemaire* 6g  *Pogostemon cablin（Blanco）Benth.* 10g | Western medicine routine treatment: (1) Rest in bed, strengthen supportive treatment, ensure sufficient heat, pay attention to water and electrolyte balance, maintain a stable internal environment, closely monitor vital signs, oxygen saturation, etc. (2) 5 million U of α-interferon added to 2 mL of sterile water for injection, 2 times a day, aerosol inhalation; lopinavir/ritonavir tablets, 2 tablets each time, 2 times a day, orally. |
| **Ding XJ, 2020** | 1 | QFTXFZ: 1 dose/day, 2 times/day | QFTXFZ:  *Astragalus membranaceus（Fisch.） Bge.var.mongholicus（Bge.）Hsiao* 6g  *Gypsum Fibrosum* 20g  *Prunus armeniaca L.* 10g  *Lonicera japonica Thunb* 30g  *Forsythia suspensa （Thunb.） Vahl* 15g  *Phragmites communis Trin.* 30g  *Coix lacryma-jobi L.var.ma-yuen(Roman.) Stapf* 30g  *Bombyx mori Linnaeus* 10g  *Cryptotympana pustulata Fabricius* 10g  *Polygonum cuspidatum Sieb. et Zucc.* 15g  *Curcuma Longa L.* 10g  *Paeonia lactiflora Pall.* 10g  *Pseudostellaria heterophylla（Miq.）Pax ex Pax et Hoffm.* 20g  *Glycyrrhiza uralensis Fisch.* 15g | NHC-China guidelines (version 5.0) |
| **Qiu M, 2020** | 1 | MXXF: 150 mL/time, 3 times/day | *Ephedra sinica Stapf* 9g  *Prunus armeniaca L.* 12g  *Gypsum Fibrosum* 15-30g  *Fritillaria thunbergii Miq.* 12g  *Bombyx mori Linnaeus* 15g  *Cryptotympana pustulata Fabricius* 10g  *Curcuma Longa L.* 10g  *Platycodon grandiflorum （Jacq.）A.DC.* 12g  *Citrus aurantium L.* 12g  *Amomum tsao-ko Crevost et Lemaire* 9g  *Amomum kravanh Pierre ex Gagnep.* 12g | NHC-China guidelines (version 5.0) |
| **Xiong WZ, 2020** | 1 | XFBD (1 pouch of 200 ml each time, 2 times/day) | XBD: *Ephedra sinica Stapf* 8g  *Prunus armeniaca L.* 15g  *Gypsum Fibrosum* 30g  *Atractylodes lancea（Thunb.）DC.* 10g  *Coix lacryma-jobi L.var.ma-yuen(Roman.) Stapf* 30g  *Pogostemon cablin（Blanco）Benth.* 15g  *Polygonum cuspidatum Sieb. et Zucc.* 20g  *Verbena officinalis L.* 30g  *Descurainia sophia（L.）Webb. ex Prantl.* 15g  *Phragmites communis Trin.* 30g  *Artemisia annua L.* 25g  *Citrus reticulata Blanco* 20g  *Glycyrrhiza uralensis Fisch.* 10g | NHC-China Guideline: Conventional treatment. |
| **Zhang CY, 2020** | 2 | XBJ: 50ml/time,2 times a day | NR | Conventional antiviral (aerosol inhalation of alpha-interferon, oral arbidol granules) and symptomatic and supportive treatment |
| **Yang MB, 2020** | 2 | RYN: 10~ 20mL,2~4 times/day. | RYN: *Taraxacum mongolicum Hand. -Mazz.*  *Polygonum cuspidatum Sieb. et Zucc.*  *Patrinia scabiosaefolia*  *Scutellaria barbata D.Don* | Lopinavir (ritonavir) tablets 50 mg, 2 capsules/time, 2 times/d; alpha-interferon (50 μg/support), 5 million U or equivalent dose each time, add sterile water for injection 2 mL, 2 times/d. Arbidol hydrochloride tablets (0.1 g/tablet) orally, 2 tablets/time, 3 times/d; Ribavirin injection (1 mL/100 mg) intravenous infusion, 0.5 g/time, 2 times/d. |
| **Feng Y, 2021** | 2 | YDJD: 12 g or 24 g, three times/day. | *Ephedrae Herba*  *Gypsum Fibrosum*  *Mori Cortex, Scutellariae Radix*  *Lepidii Semen*  *Lonicerae Japonicae Flos*  *Scrophulariae Radix*  *Moutan Cortex,*  *Rehmanniae Radix*  *Atractylodis Macrocephalae Rhizoma*  *Cimicifugae Rhizoma* | Routine treatment generally consisted of supportive treatments, such as oxygen and symptomatic therapies, according to the Diagnosis and Treatment Protocol for Coronavirus Pneumonia (Trial version 8) at the discretion of the attending clinicians. |
| **Zhang N, 2020** | 2 | MXSG: 200mL/dose,1 dose/day, 2 times/day | *Gypsum Fibrosum* 30g  *Ephedra sinica Stapf* 10g  *Platycodon grandiflorum （Jacq.）A.DC .* 15g  *Eriobotrya japonica（Thunb.）Lindl.* 12g  *Prunus armeniaca L.* 12g  *Atractylodes macrocephala Koidz.* 10g  *Poria cocos（Schw.）Wolf* 10g  *Fritillaria cirrhosa D.Don* 9g  *Scutellaria baicalensis Georgi* 9g  *Morus alba L.* 9g  *Astragalus membranaceus（Fisch.） Bge.var.mongholicus（Bge.）Hsiao* 6g | NHC-China guidelines (Trial version 4.0) |
| **Lan J, 2020** | 2 | QSPDFZ: 250mL/time, 2 times/day | *Pogostemon cablin（Blanco）Benth.* 30g  *Magnolia officinalis Rehd.et Wils.* 12g  *Pinellia ternata（Thunb.） Breit.* 15g  *Amomum kravanh Pierre ex Gagnep.* 10g  *Poria cocos（Schw.）Wolf* 20g  *Coix lacryma-jobi L.var.ma-yuen(Roman.) Stapf*  12g  *Polyporus umbellatus（Pers.） Fries* 10g  *Alisma orientale（Sam.）Juzep.* 10g  *Glycine max (L.) Merr.* 10g  *Citrus reticulata Blanco* 12g  *Atractylodes macrocephala Koidz. 2*0g  *Codonopsis pilosula (Franch.)Nannf.* 30g  *Amomum villosum Lour.* 6g  *Glycyrrhiza uralensis Fisch.* 6g  *Atractylodes lancea（Thunb.）DC.* 15g  *Angelica dahurica（Fisch.ex Hoffm.）Benth.et Hook.* 15g  *Perilla frutescens(L.) Britt.* 12g  *Platycodon grandiflorum （Jacq.）A.DC.* 15g  *Astragalus membranaceus（Fisch.） Bge.var.mongholicus（Bge.）Hsiao* 6g  *Gypsum Fibrosum* 20-30g  *Prunus armeniaca L.* 10g  *Scutellaria baicalensis Georgi* 30g  *Saposhnikovia divaricata （Turcz.）Schischk.* 10g  *Lonicera japonica Thunb.*  30g | Administer antiviral [lopinavir/ritonavir (Kelitzia, 200 mg/50 mg per capsule), 2 capsules each time, 2 times a day, 10 days as a course of treatment; After receiving or after 1 course of treatment, change to Arbidol 200 mg, 3 times a day, 10 days as 1 course of treatment; α-interferon 5 000 kU, inhalation, 2 times a day, 15 days as 1 course a course of treatment] and anti-infection (such as quinolone antibiotics and (or) third-generation cephalosporins for combined bacterial infection); if the white blood cell count (WBC) is significantly reduced, subcutaneous injection of recombinant granulocyte-stimulating factor 3 000 U is given , Prophylactic use of antibiotics as appropriate; nutritional support and psychological intervention at the same time; corresponding supportive treatment for those with underlying diseases. Use 2 courses of treatment in a row. |
| **Chen L, 2020a** | 2 | GLXD: 100 mL/time, 3 times/day | GLXD: *TALCUM* 45g  *Artemisia capillaris Thunb.* 30 g  *Scutellaria baicalensis Georgi* 30 g  *Acorus tatarinowii Schott* 18g  *Akebia quinata （Thunb.）Decne.* 15g  *Fritillaria cirrhosa D.Don* 15g  *Pogostemon cablin（Blanco）Benth.* 12g  *Amomum kravanh Pierre ex Gagnep.* 12g  *Forsythia suspensa （Thunb.） Vahl* 12g  *Mentha canadensis Linnaeus* 12g  *Iris tectorum Maxim.* 12g  *Glycyrrhiza uralensis Fisch.* 10g  *Dioscorea opposita Thunb.* 30g | Routine treatment is bed rest and supportive treatment; when oxygen saturation is low, catheter oxygen is given; low fever is given physical cooling, high fever (≥38.3 ℃), oral ibuprofen suspension is given to assist in antipyretic (10 mL/time), 3 times/d); those with severe sputum were given ambroxol hydrochloride tablets for oral expectoration (0.6 g/time, 3 times/d); patients with infection were given oral anti-infection with moxifloxacin tablets (0.4 g/time, 3 times/d); 1 time/d); at the same time, the viral drug Arbidol hydrochloride capsule was given orally (0.2 g/time, 3 times/d). |
| **Tian JX, 2020** | 3 | HSY: The exposed group included patients who were administered HSYF for more than 2 days. If there is no adverse effect or disease progression, HSYF can be taken continuously until recovery | NR | The control group included patients who were diagnosed with COVID-19 but were not administered HSYF (including decoction, granules, etc.) at the same time. |
| **Zhang X, 2021** | 2 | TRQ：three times/day, three pills /time, from the day of admission to the day before discharge. | TRQ: *Scutellaria baicalensis Georgi*  *bear bile powde*  *goat horn*  *Lonicera japonica Thunb.*  *Forsythia suspensa （Thunb.） Vahl* | NHC-China Guideline (version 6.0): Both groups were given oxygen therapy, antiviral medications, antibiotics and other conventional treatments from Western medicine. |
| **Li HL, 2020** | 2 | self-made formula: 300ml, 2times/a day | *Bupleurum chinense DC.* 18g  *Scutellaria baicalensis Georgi* 15g  *Zingiber officinale Rosc.* 8g  *Ziziphus jujuba Mill.* 10g  *Zingiber officinale Rosc. Pinellia ternata（Thunb.） Breit.* 10g  *Glycyrrhiza uralensis Fisch.* 6g  *Aucklandia lappa Decne.* 10g  *Scrophularia ningpoensis Hemsl.* 15g  *Citrus reticulata Blanco* 10g  *Pogostemon cablin（Blanco）Benth.* 10g  *Atractylodes macrocephala Koidz.* 10g  *Coix lacryma-jobi L.var.ma-yuen(Roman.) Stapf*  30g  *Platycodon grandiflorum （Jacq.）A.DC.* 10g  *Adenophora tetraphylla (Thunb.)Fisch.* 10g | Arbidol, aerosol inhalation of alpha-interferon, Kaletra, anti-infection and related symptomatic support, oxygen therapy if necessary |
| **Zhang CT, 2020** | 1 | JWDY: One dose/ day, divided into three times | JWDY: *Astragalus membranaceus（Fisch.） Bge.var.mongholicus（Bge.）Hsiao* 10g  *Prunus armeniaca L.* 15g  *Gypsum Fibrosum* 20g  *Trichosanthes kirilowii Maxim.* 20g  *Rheum palmatum L.* 6g  *Descurainia sophia（L.）Webb. ex Prantl.* 10g  *Prunus persica（L.）Batsch* 10g  *Amomum tsao-ko Crevost et Lemaire* 6g  *Areca catechu L.* 10g  *Atractylodes lancea（Thunb.）DC.* 10g | NHC-China guidelines (Trial version 4.0) |

1: RCT; 2: historical control study; 3: control study

**Table S6 Risk of bias of included RCTs**

| **Study** | **Bias from the randomization process generated** | **Bias due to deviations from the intended intervention** | **Bias due to missing data** | **Bias due to measurement of the outcome** | **Bias in selection of the reported results** | **Other bias** |
| --- | --- | --- | --- | --- | --- | --- |
| **Wang Y,2021** | High | High | Low | Low | Probably low | Low |
| **Yu P, 2020** | Probably high | High | Low | Probably low | Probably low | Low |
| **Zhang YL, 2020** | High | High | Low | Probably low | Probably low | Low |
| **Hu F, 2020** | Probably high | High | High | Low | Probably low | Probably low |
| **Duan C, 2020** | Probably high | High | High | Probably low | Probably low | Probably low |
| **Zhao C, 2021** | Probably high | High | Low | Low | Probably low | Low |
| **Liu J, 2021** | Probably high | High | Low | Low | Low | Low |
| **Hu K, 2020** | Probably high | High | Low | Probably low | Probably low | Low |
| **An XD, 2021** | Probably high | High | Low | Probably low | Low | Low |
| **Wang LQ, 2020** | Probably high | High | Low | Probably low | Probably low | Low |
| **Ma QH, 2021** | Probably high | High | Low | Probably low | Probably low | Low |
| **Ni L, 2021** | High | High | Low | Probably low | Probably low | Low |
| **Xu XL, 2021** | Probably high | High | Low | Probably low | Probably low | Low |
| **Zhang XY, 2021** | Probably low | Low | Low | Probably low | Low | Low |
| **Sun SQ, 2021** | Low | High | Low | Low | Probably low | Low |
| **Yan YS, 2021** | Probably high | High | Low | Probably low | Probably low | Low |
| **Wang JB, 2020** | Low | Probably low | Low | Low | Probably low | Low |
| **Zeng CC, 2021** | Probably low | Probably high | Low | Probably low | Low | Low |
| **Li ZJ, 2021** | Probably high | High | Low | Probably low | Probably low | Low |
| **Ping XH, 2021** | Probably high | High | Low | Probably low | Probably low | Probably low |
| **Wu R, 2021** | Probably high | High | Low | Probably low | Probably low | Low |
| **Jin W, 2020** | Probably high | High | Low | Probably low | Probably low | Low |
| **Wang YL, 2020** | Probably high | High | Low | Low | Probably low | Low |
| **AI XY, 2020** | Probably high | High | Low | Low | Probably low | Low |
| **Lin FF, 2020** | Probably high | High | Low | Low | Probably low | Low |
| **Fu XX, 2020** | Probably high | High | Low | Low | Probably low | Low |
| **Ding XJ, 2020** | Probably high | High | Low | Low | Probably low | Probably low |
| **Qiu M, 2020** | Probably high | High | Low | Low | Probably low | Probably low |
| **Sun HM, 2020** | Probably high | High | Low | Low | Probably low | Low |
| **AI XY, 2020** | Probably high | High | Low | Low | Probably low | Probably low |
| **Xiong WZ, 2020** | Probably high | High | Low | Probably high | Low | Low |
| **Xiao MZ, 2020** | Probably high | High | Low | Probably high | Probably low | Low |
| **Zhang CT， 2020** | High | High | Low | Probably low | Probably low | Probably low |
| **Zhang L, 2022** | Probably low | High | Low | Probably low | Probably high | Low |

**Table S7 Risk of bias of included OBs**

| **Study** | **Bias due to confunding** | **Bias in selection of participants into the study** | **Bias from the exposure** | **Bias due to missing data** | **Bias due to measurement of the outcome** | **Bias in selection of the reported results** |
| --- | --- | --- | --- | --- | --- | --- |
| **Zeng XH, 2020** | High | Low | Low | Low | Low | Probably low |
| **Zhang CY, 2020** | High | Low | Low | Low | Low | Probably low |
| **Yu HY, 2020** | High | Low | High | Low | Low | Probably low |
| **Xiao Q, 2020** | High | Low | Low | Low | Probably Low | Probably low |
| **Shi NN, 2020** | High | Low | Low | Low | Low | Probably Low |
| **Chen L, 2020b** | Low | Low | Low | Low | Probably Low | Probably low |
| **Yang MB, 2020** | High | Low | Low | Low | Probably Low | Probably Low |
| **Zhang LH, 2021** | Low | Low | Low | Low | Low | Probably Low |
| **Feng Y, 2021** | Low | Low | Low | Low | Low | Probably low |
| **Chen J, 2020** | High | Low | Low | Low | Probably Low | Probably low |
| **Liu L, 2021** | High | Low | Low | Low | Low | Probably low |
| **Wang QL, 2021** | High | Low | Low | Low | Low | Probably low |
| **Liu ZL, 2020** | High | Low | Low | Low | Probably Low | Probably low |
| **Zhang N, 2020** | High | Low | Low | Low | Probably Low | Probably low |
| **Chen L, 2020a** | Low | Low | Low | Low | Probably Low | Probably low |
| **Lan J, 2020** | High | Low | Low | Low | Probably Low | Probably low |
| **Tian JX, 2020** | Low | Low | Low | Low | Low | Probably low |
| **Zhang X,2021** | High | Low | Low | Low | Low | Low |
| **Xin SY, 2020** | High | Low | Low | Low | Low | Probably low |
| **Chen RB, 2020** | High | Low | Low | Low | Probably Low | Probably low |
| **Ai ZZ, 2020** | High | Low | Low | Low | Low | Probably low |
| **Sun YN, 2021** | High | Low | Low | Low | Low | Probably low |
| **Li HL, 2020** | High | Low | Low | Low | Low | Probably low |
| **Yao KT, 2020** | High | Low | Low | Low | Probably Low | Probably low |

**Table S8** **Incidence and difference between groups of adverse reactions**

| TCM | Study Design | No. of Studies | No. of Participations (I/C) | Adverse Reactions | Intervention Group | | Control Group | | P Value |
| --- | --- | --- | --- | --- | --- | --- | --- | --- | --- |
|  |  |  |  |  | Event | % | Event | % |  |
| QFPD | RCT | 1 | 140 (70/70) | Flustered | 0 | 0.00% | 2 | 2.86% | 0.30 |
|  |  |  |  | Nausea | 1 | 1.43% | 4 | 5.71% | 0.21 |
|  |  |  |  | Tiredness | 1 | 1.43% | 3 | 4.29% | 0.34 |
|  | OB | 1 | 8939(2568/6317) | Acute hepatic injury | 0.96 [95% CI: 0.81, 1.14] # | | | | 0.658 |
|  |  |  |  | Acute kidney injury | 0.96 [95% CI: 0.81, 1.14] # | | | | 0.318 |
|  | OB | 1 | 72(42/30) | Liver dysfunction | 9 | 21.43% | 5 | 16.67% | 0.62 |
|  |  |  |  | Dyslipidemia | 7 | 16.67% | 5 | 16.67% | 1.00 |
|  |  |  |  | Diarrhea | 4 | 9.52% | 2 | 6.67% | 0.67 |
|  | OB | 1 | 45(23/22) | Nausea and diarrhea | 2 | 8.70% | 3 | 13.64% | 0.60 |
|  | OB | 1 | 295(199/96) | Total | 0 | 0.00% | 0 | 0.00% | NA |
| LHQW | RCT | 1 | 284(142/142) | Liver dysfunction | 32 | 22.54% | 32 | 22.54% | 1.00 |
|  |  |  |  | Renal dysfunction | 8 | 5.63% | 11 | 7.75% | 0.48 |
|  |  |  |  | Headache | 1 | 0.70% | 1 | 0.70% | 1.00 |
|  |  |  |  | Nausea | 6 | 4.23% | 5 | 3.52% | 0.76 |
|  |  |  |  | Vomiting | 2 | 1.41% | 3 | 2.11% | 0.65 |
|  |  |  |  | Diarrhea | 8 | 5.63% | 19 | 13.38% | 0.03 |
|  |  |  |  | Loss of appetite | 8 | 5.63% | 6 | 4.23% | 0.59 |
|  | RCT | 2 | 579(289/290) | Total | 65 | 22.49% | 77 | 26.55% | 0.16 |
|  | OB | 1 | 108(68/40) | Total | 0 | 0.00% | 0 | 0.00% | NA |
| JYH | RCT | 2 | 307(196/111) | Diarrhea | 2 | 1.02% | 0 | 0.00% | 0.65 |
| JHQG | RCT | 1 | 123(82/41) | Diarrhea | 27 | 32.93% | 0 | 0.00% | 0.02 |
|  | OB | 1 | 80(44/36) | Abnormal routine blood workup | 0 | 0.00% | 0 | 0.00% | NA |
|  |  |  |  | Abnormal liver and kidney function | 0 | 0.00% | 0 | 0.00% | NA |
| HSBD | RCT | 2 | 565(268/297) | Diarrhea | 14 | 5.22% | 9 | 3.03% | 0.22 |
|  |  | 1 | 202(100/102) | Abdominal discomfort | 2 | 2.00% | 3 | 2.94% | 0.67 |
|  |  |  |  | Loss of appetite | 3 | 3.00% | 0 | 0.00% | 0.19 |
|  |  |  |  | Anxiety | 0 | 0.00% | 2 | 1.96% | 0.30 |
|  |  |  |  | Oral ulcer | 1 | 1.00% | 1 | 0.98% | 0.99 |
|  |  |  |  | Shortness of breath | 0 | 0.00% | 2 | 1.96% | 0.30 |
|  |  |  |  | Constipation | 0 | 0.00% | 1 | 0.98% | 0.51 |
|  |  |  |  | Vomiting | 1 | 1.00% | 0 | 0.00% | 0.49 |
|  |  |  |  | Itchy skin | 0 | 0.00% | 1 | 0.98% | 0.51 |
|  |  |  |  | Lower extremity edema | 0 | 0.00% | 1 | 0.98% | 0.51 |
|  |  |  |  | Dry eye | 1 | 1.00% | 0 | 0.00% | 0.49 |
|  |  |  |  | Limb pain | 0 | 0.00% | 1 | 0.98% | 0.51 |
|  | OB | 1 | 40(20/20) | Abnormal alanine aminotransferase | 6 | 30.00% | 11 | 55.00% | 0.13 |
|  |  |  |  | Abnormal glutamic-oxalacetic transaminase | 4 | 20.00% | 9 | 45.00% | 0.11 |
|  |  |  |  | Abnormal serum creatinin | 5 | 25.00% | 7 | 35.00% | 0.49 |
|  |  |  |  | Abnormal creatine kinase | 7 | 35.00% | 9 | 45.00% | 0.52 |
| HSBD+XYP+SM+XBJ | OB | 1 | 40(20/20) | Abnormal alanine aminotransferase | 11 | 55.00% | 11 | 55.00% | 1.00 |
|  |  |  |  | Abnormal glutamic-oxalacetic transaminase | 10 | 50.00% | 9 | 45.00% | 0.75 |
|  |  |  |  | Abnormal serum creatinin | 8 | 40.00% | 7 | 35.00% | 0.74 |
|  |  |  |  | Abnormal creatine kinase | 6 | 30.00% | 9 | 45.00% | 0.34 |
| RDN | RCT | 1 | 50(27/20) | Total | 0 | 0.00% | 1 | 4.35% | 0.44 |
|  |  | 1 | 157(77/80) | Liver dysfunction | 0 | 0.00% | 2 | 2.50% | 0.31 |
|  |  |  |  | Nausea | 2 | 2.60% | 1 | 1.25% | 0.55 |
|  |  |  |  | Diarrhea | 1 | 1.30% | 0 | 0.00% | 0.48 |
|  |  |  |  | Bitter taste | 0 | 0.00% | 1 | 1.25% | 0.51 |
|  |  |  |  | Stomach discomfort | 1 | 1.30% | 0 | 0.00% | 0.48 |
|  |  |  |  | Loss of appetite | 0 | 0.00% | 1 | 1.25% | 0.51 |
| SFJD | OB | 2 | 400(200/200) | Chest tightness | 0 | 0.00% | 0 | 0.00% | NA |
|  |  |  |  | Allergic reaction | 2 | 1.00% | 4 | 2.00% | 0.42 |
|  |  | 1 | 200(100/100) | Abdominal pain and diarrhea | 2 | 2.00% | 1 | 1.00% | 0.57 |
|  |  | 1 | 68(34/34) | Abdominal distension | 0 | 0.00% | 1 | 2.94% | 0.50 |
|  |  |  |  | Acid reflux | 0 | 0.00% | 1 | 2.94% | 0.50 |
|  |  |  |  | Elevated abnormal liver enzymes | 0 | 0.00% | 1 | 2.94% | 0.50 |
|  |  | 1 | 200(100/100) | Nausea | 2 | 2.00% | 1 | 1.00% | 0.57 |
|  |  |  |  | Abdominal pain | 2 | 2.00% | 2 | 2.00% | 1.00 |
|  |  |  |  | Diarrhea | 1 | 1.00% | 1 | 1.00% | 1.00 |
| JWYPF | RCT | 1 | 54(30/24) | Nausea, vomiting and loss of appetite | 3 | 10.00% | 8 | 33.33% | 0.05 |
|  |  |  |  | Diarrhea | 1 | 3.33% | 1 | 4.17% | 0.87 |
|  |  |  |  | Chest tightness | 2 | 6.67% | 2 | 8.33% | 0.82 |
|  |  |  |  | Itchy skin | 0 | 0.00% | 1 | 4.17% | 0.03 |
|  |  |  |  | Total | 6 | 20.00% | 12 | 50.00% | 0.57 |
| SHL | RCT | 1 | 235(176/59) | Rash | 3 | 1.70% | 0 | 0.00% | 0.57 |
|  |  |  |  | Skin allergies | 1 | 0.57% | 0 | 0.00% | 0.99 |
|  |  |  |  | Gastrointestinal discomfort | 5 | 2.84% | 0 | 0.00% | 0.37 |
|  |  |  |  | Nausea | 2 | 1.14% | 0 | 0.00% | 0.73 |
|  |  |  |  | Vomiting | 2 | 1.14% | 0 | 0.00% | 0.73 |
|  |  |  |  | Diarrhea | 2 | 1.14% | 0 | 0.00% | 0.73 |
|  |  |  |  | Abdominal distension and pain | 1 | 0.57% | 0 | 0.00% | 0.99 |
|  |  |  |  | Loss of appetite | 1 | 0.57% | 0 | 0.00% | 0.99 |
|  |  |  |  | Hypokalemia | 1 | 0.57% | 0 | 0.00% | 0.99 |
|  |  |  |  | Constipation | 0 | 0.00% | 1 | 1.69% | 0.18 |
|  |  |  |  | Urinary tract infection | 1 | 0.57% | 0 | 0.00% | 0.99 |
|  |  |  |  | Increased aspartate aminotransferase | 3 | 1.70% | 0 | 0.00% | 0.57 |
|  |  |  |  | Increased alanine aminotransferase | 4 | 2.27% | 1 | 1.69% | 0.79 |
|  |  |  |  | Increased g-glutamyl transpeptidase | 4 | 2.27% | 0 | 0.00% | 0.45 |
|  |  |  |  | Hyperlipidemia | 5 | 2.84% | 0 | 0.00% | 0.37 |
| XYP | RCT | 1 | 130(65/65) | Chest pain | 15 | 23.08% | 11 | 16.92% | 0.38 |
|  |  |  |  | Diarrhea | 10 | 15.38% | 15 | 23.08% | 0.27 |
|  |  |  |  | Nausea | 11 | 16.92% | 8 | 12.31% | 0.46 |
|  |  |  |  | Tiredness | 6 | 9.23% | 4 | 6.15% | 0.51 |
|  |  |  |  | Abdominal discomfort | 3 | 4.62% | 5 | 7.69% | 0.47 |
|  |  |  |  | Shortness of breath | 4 | 6.15% | 4 | 6.15% | 1.00 |
|  |  |  |  | Dizziness | 4 | 6.15% | 3 | 4.62% | 0.70 |
| LS | RCT | 1 | 80(40/40) | Total | 0 | 0.00% | 0 | 0.00% | NA |
| YHQF | RCT | 1 | 40(20/20) | Nausea | 2 | 10.00% | 2 | 10.00% | 1.00 |
|  |  |  |  | Liver dysfunction | 3 | 15.00% | 2 | 10.00% | 0.54 |
|  |  |  |  | Mild diarrhea | 4 | 20.00% | 4 | 20.00% | 1.00 |
| Keguan-1 | RCT | 1 | 29(15/14) | Diarrhea | 9 | 60.00% | 8 | 57.14% | 0.88 |
|  |  |  |  | Anorexia | 4 | 26.67% | 5 | 35.71% | 0.60 |
|  |  |  |  | Nausea | 2 | 13.33% | 3 | 21.43% | 0.57 |
|  |  |  |  | Stomach pain | 2 | 13.33% | 3 | 21.43% | 0.57 |
|  |  |  |  | Allergic reaction | 0 | 0.00% | 1 | 7.14% | 0.47 |
|  |  |  |  | Sepsis | 0 | 0.00% | 1 | 7.14% | 0.47 |
| TJQW | RCT | 2 | 132(65/67) | Total | 0 | 0.00% | 0 | 0.00% | NA |
| QFTXFZ | RCT | 1 | 100(51/49) | Decreased total blood count | 3 | 5.88% | 2 | 4.08% | 0.68 |
|  |  |  |  | Liver dysfunction | 2 | 3.92% | 3 | 6.12% | 0.62 |
|  |  |  |  | Renal dysfunction | 0 | 0.00% | 0 | 0.00% | NA |
| LHQK | RCT | 1 | 148(72/72) | Total | 7 | 9.72% | 11 | 15.28% | 0.32 |
|  |  |  |  | Heart dysfunction | 0 | 0.00% | 1 | 1.39% | 0.68 |
|  |  |  |  | Gastrointestinal dysfunction | 1 | 1.39% | 6 | 8.33% | 0.09 |
|  |  |  |  | Hepatobiliary dysfunction | 1 | 1.39% | 1 | 1.39% | 1.00 |
|  |  |  |  | Infections | 1 | 1.39% | 1 | 1.39% | 1.00 |
|  |  |  |  | Metabolic disorders | 3 | 4.17% | 2 | 2.78% | 0.65 |
|  |  |  |  | Neurological disorders | 1 | 1.39% | 0 | 0.00% | 0.50 |
| XBJ | OB | 1 | 44(22/22) | Abdominal pain and diarrhea | 1 | 4.55% | 0 | 0.00% | 0.49 |
|  |  |  |  | Dry cough | 1 | 4.55% | 0 | 0.00% | 0.49 |
|  |  |  |  | Diarrhea | 0 | 0.00% | 1 | 4.55% | 0.49 |
|  |  |  |  | Total | 2 | 9.09% | 1 | 4.55% | 0.56 |
| RYN | OB | 1 | 49(26/23) | Total | 0 | 0.00% | 0 | 0.00% | NA |
| QSPDFZ | OB | 1 | 85(43/42) | Liver dysfunction | 12 | 27.91% | 10 | 23.81% | 0.67 |
|  |  |  |  | Diarrhea | 5 | 11.63% | 6 | 14.29% | 0.72 |
|  |  |  |  | Loss of appetite | 3 | 6.98% | 4 | 9.52% | 0.67 |
|  |  |  |  | Total | 20 | 46.51% | 20 | 47.62% | 0.92 |
| GLXD | OB | 1 | 230(115/115) | Elevated abnormal liver enzymes | 0 | 0.00% | 2 | 1.74% | 0.30 |
|  |  |  |  | Acid reflux | 0 | 0.00% | 2 | 1.74% | 0.30 |
|  |  |  |  | Abdominal distension | 0 | 0.00% | 1 | 0.87% | 0.50 |
|  |  |  |  | Renal dysfunction | 0 | 0.00% | 1 | 0.87% | 0.50 |
| QFDYG | OB | 1 | 84(49/35) | Total | 23 | 46.94% | 19 | 54.29% | 0.50 |
|  |  |  |  | Nausea | 11 | 22.45% | 8 | 22.86% | 0.96 |
|  |  |  |  | Liver dysfunction | 4 | 8.16% | 5 | 14.29% | 0.38 |
|  |  |  |  | Vomiting | 1 | 2.04% | 1 | 2.86% | 0.81 |
|  |  |  |  | Headache | 4 | 8.16% | 3 | 8.57% | 0.95 |
|  |  |  |  | Renal dysfunction | 3 | 6.12% | 2 | 5.71% | 0.94 |
| XFQR | RCT | 1 | 82(41/41) | Total | 0 | 0.00% | 0 | 0.00% | NA |
| XFBD | RCT | 1 | 42(22/20) | Total | 0 | 0.00% | 0 | 0.00% | NA |
| TRQ | OB | 1 | 92(25/57) | Diarrhea | 4 | 16.00% | 3 | 5.26% | 0.13 |
| JWDY | RCT | 1 | 45(22/23) | Total | 0 | 0.00% | 0 | 0.00% | NA |

# Adjusted OR with 95% Confidence Interval

**FigureS1.1 Forest plot of rate of conversion to severe cases (RCTs)**


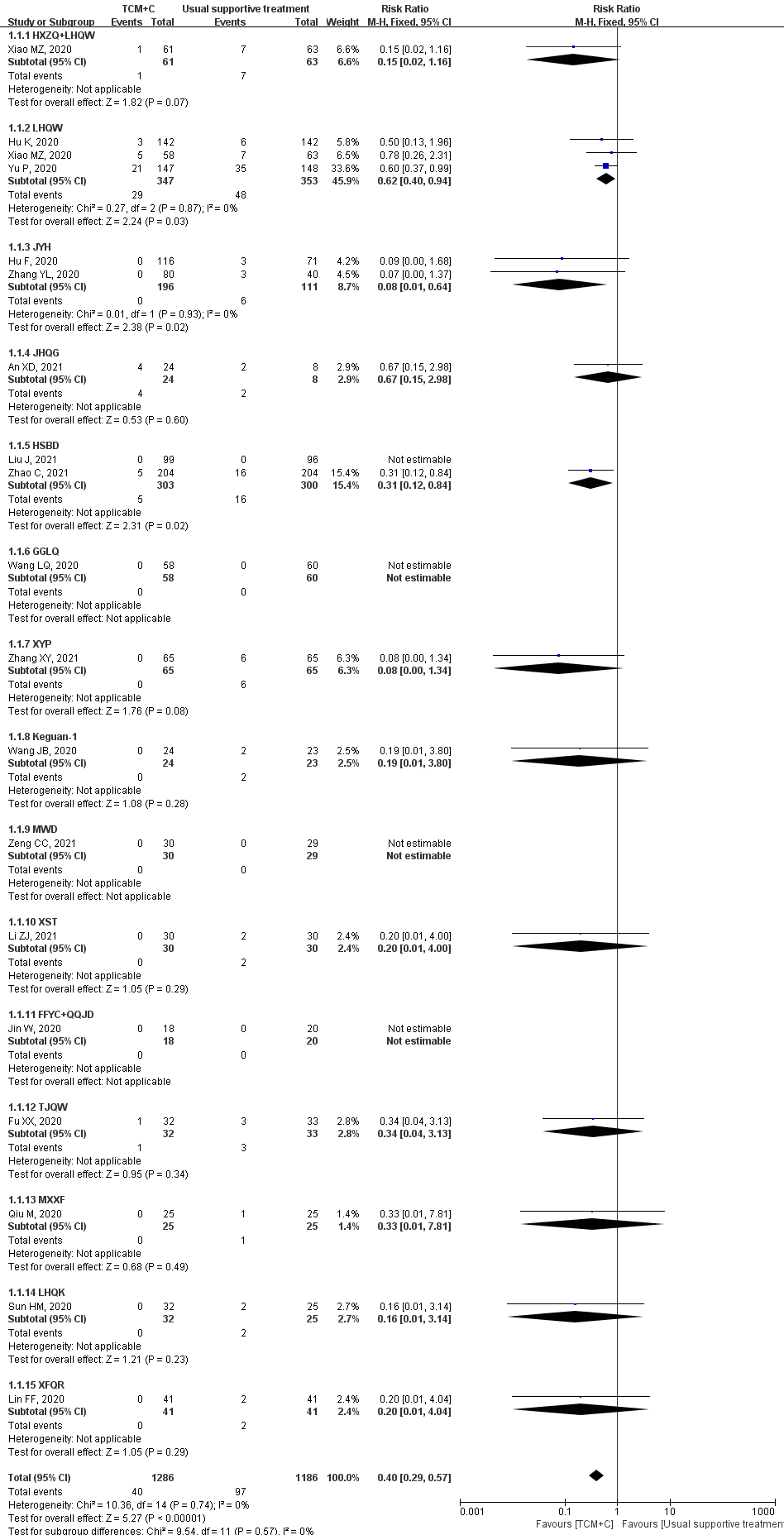


**FigureS1.2 Forest plot of rate of conversion to severe cases (OBs)**


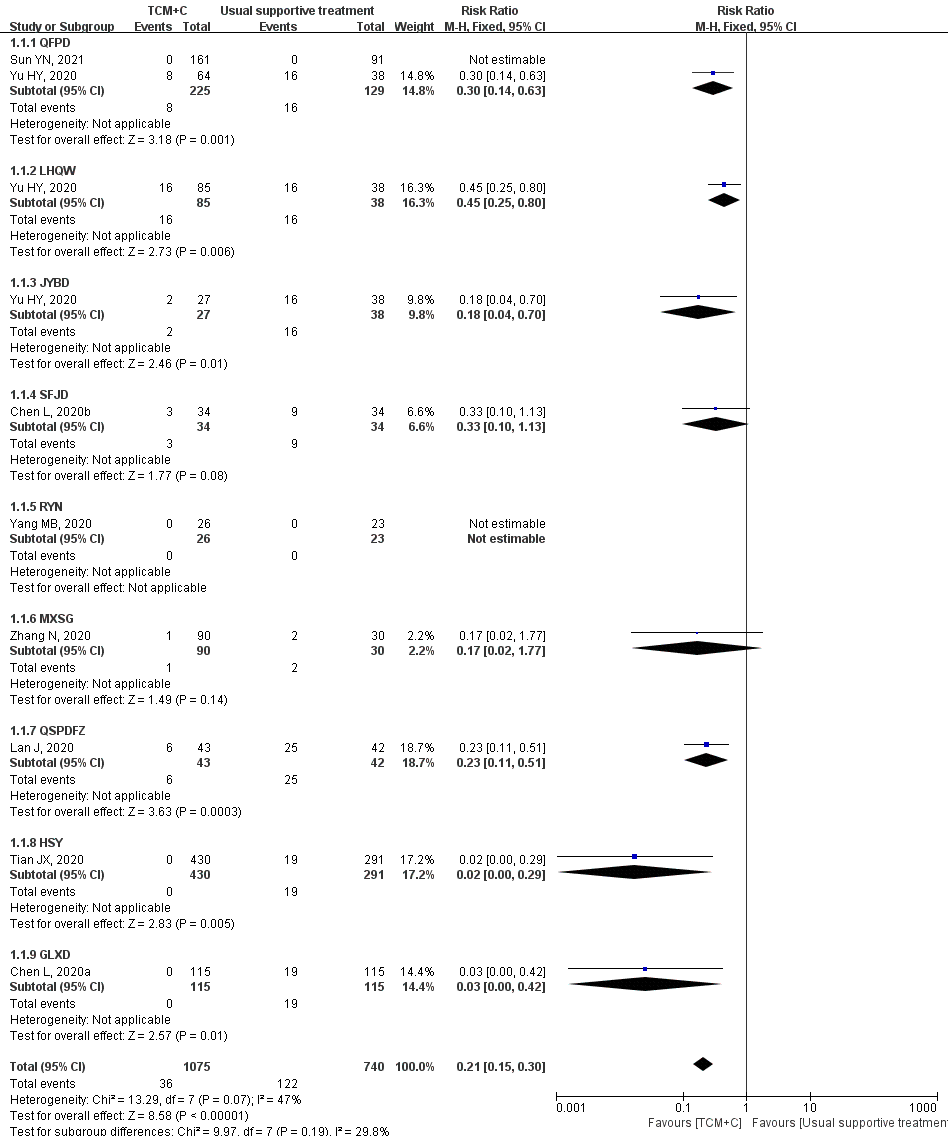


**FigureS2.1 Forest plot of time to fever resolution (RCTs)**


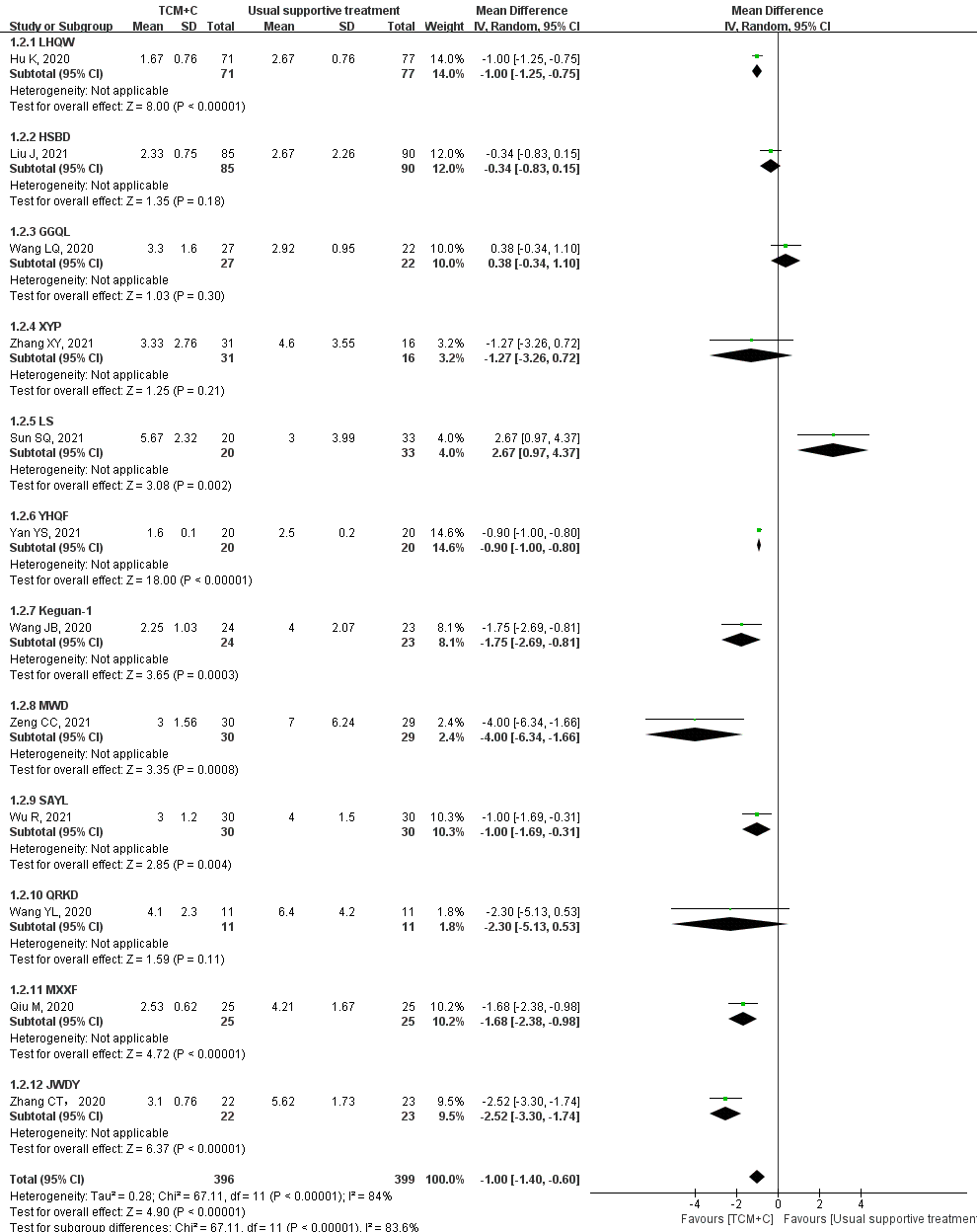


**FigureS2.2 Forest plot of time to cough resolution (RCTs)**


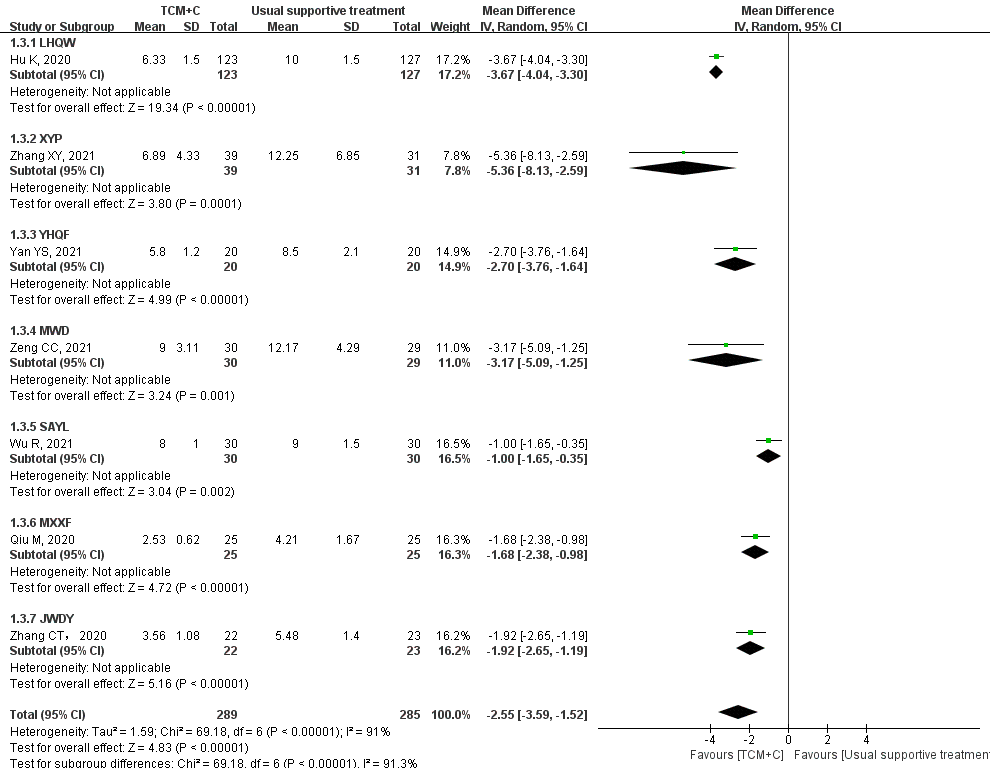


**FigureS2.3 Forest plot of time to tiredness resolution (RCTs)**


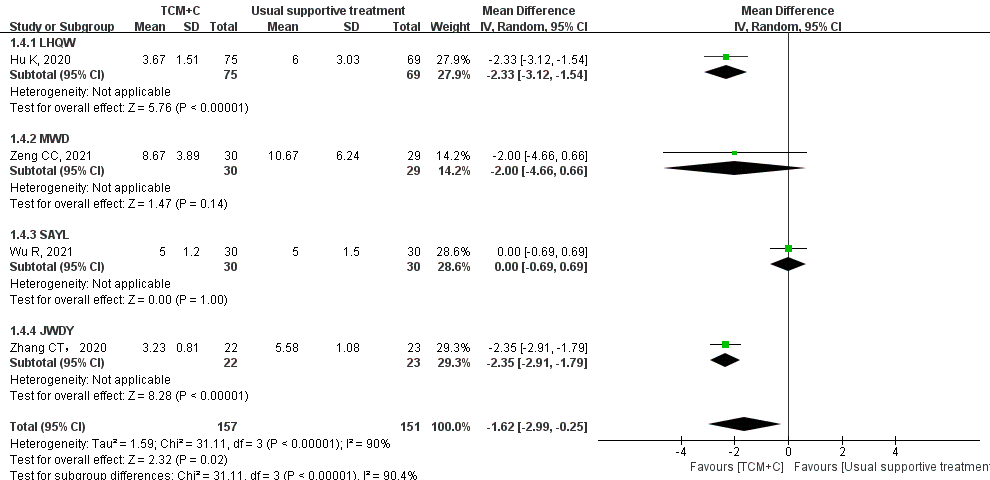


**FigureS2.4 Forest plot of time to shortness of breath resolution (RCTs)**


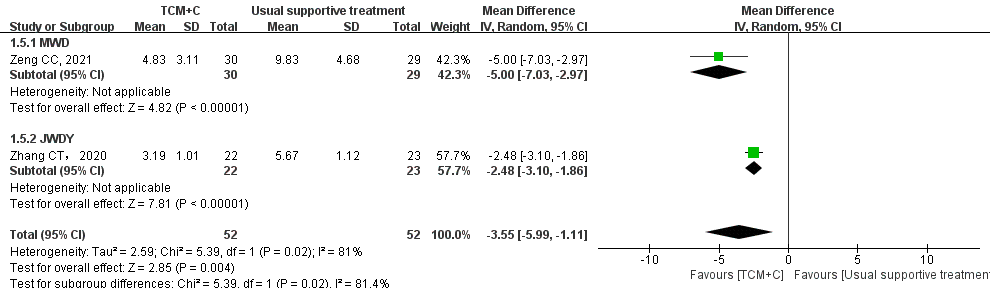


**FigureS2.5 Forest plot of time to total symptoms resolution (RCTs)**


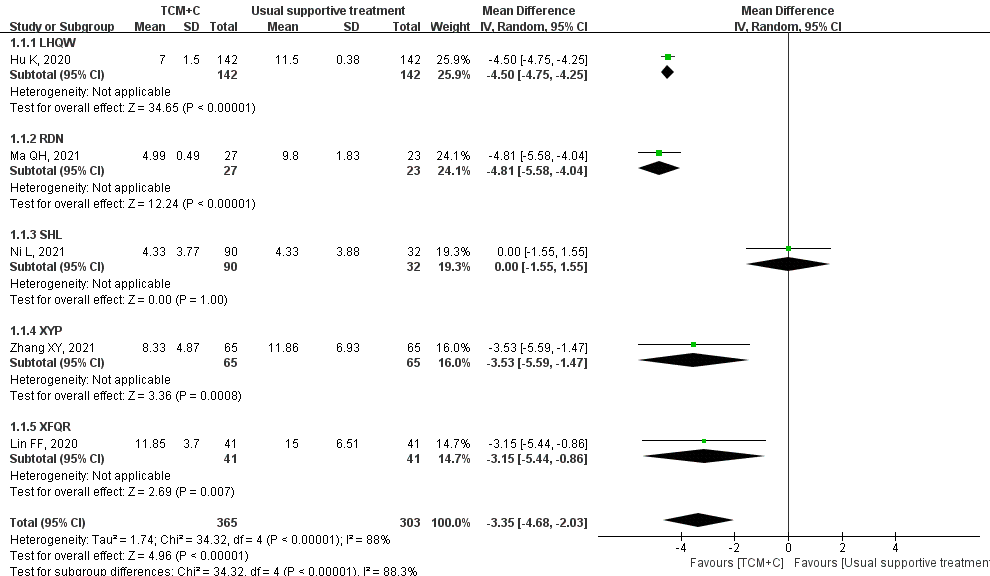


**FigureS3.1 Forest plot of time to fever resolution (OBs)**


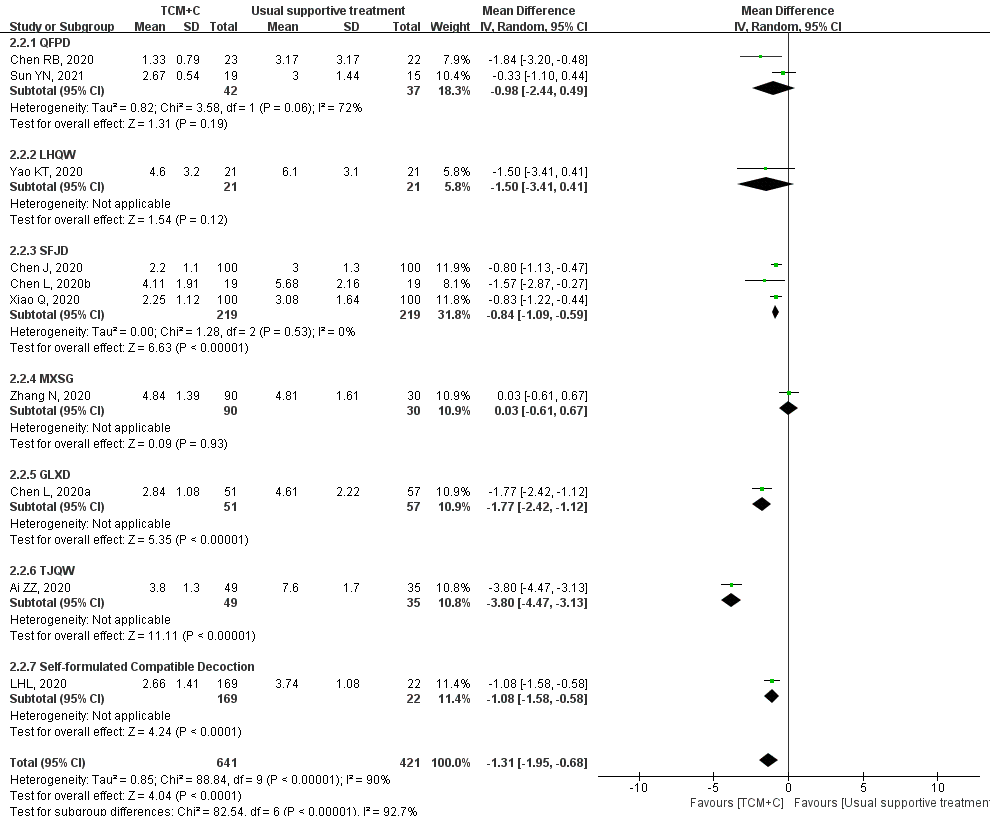


**FigureS3.2 Forest plot of time to cough resolution (OBs)**


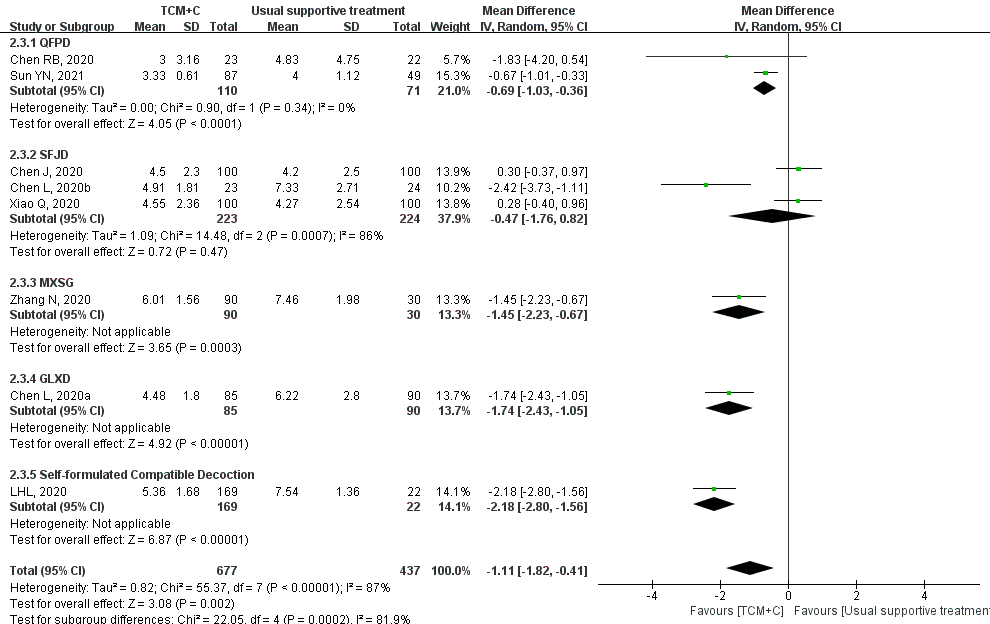


**FigureS3.3 Forest plot of time to tiredness resolution (OBs)**


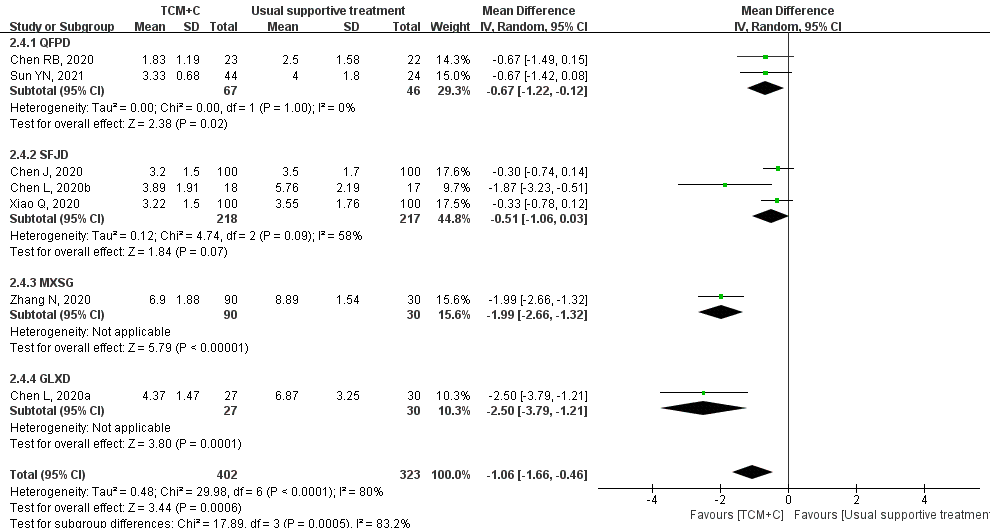


**FigureS3.4 Forest plot of time to expectoration resolution (OBs)**


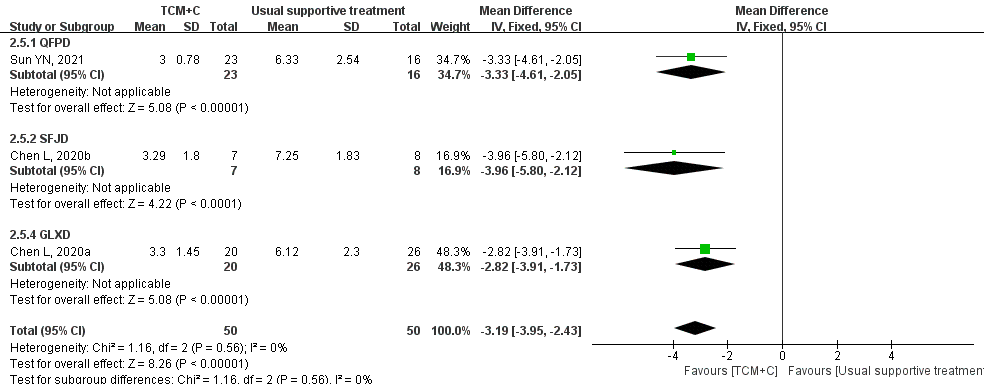


**FigureS3.5 Forest plot of time to sore throat resolution (OBs)**


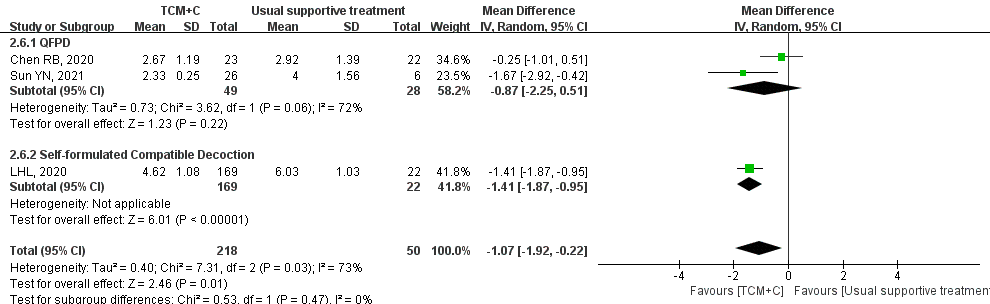


**FigureS3.6 Forest plot of time to total symptoms resolution (OBs)**


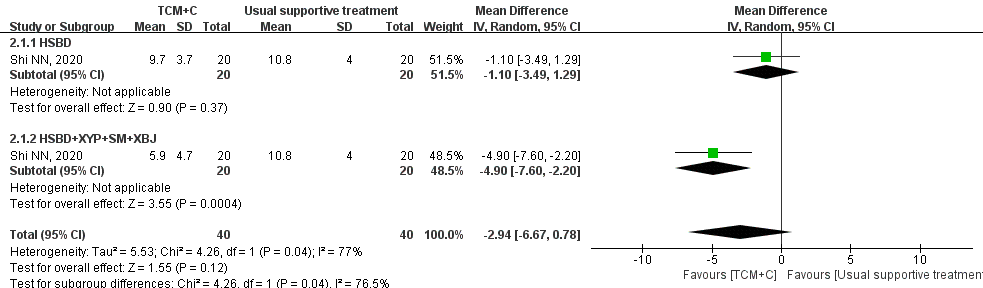


**FigureS4.1 Forest plot of length of hospital stay (RCTs)**


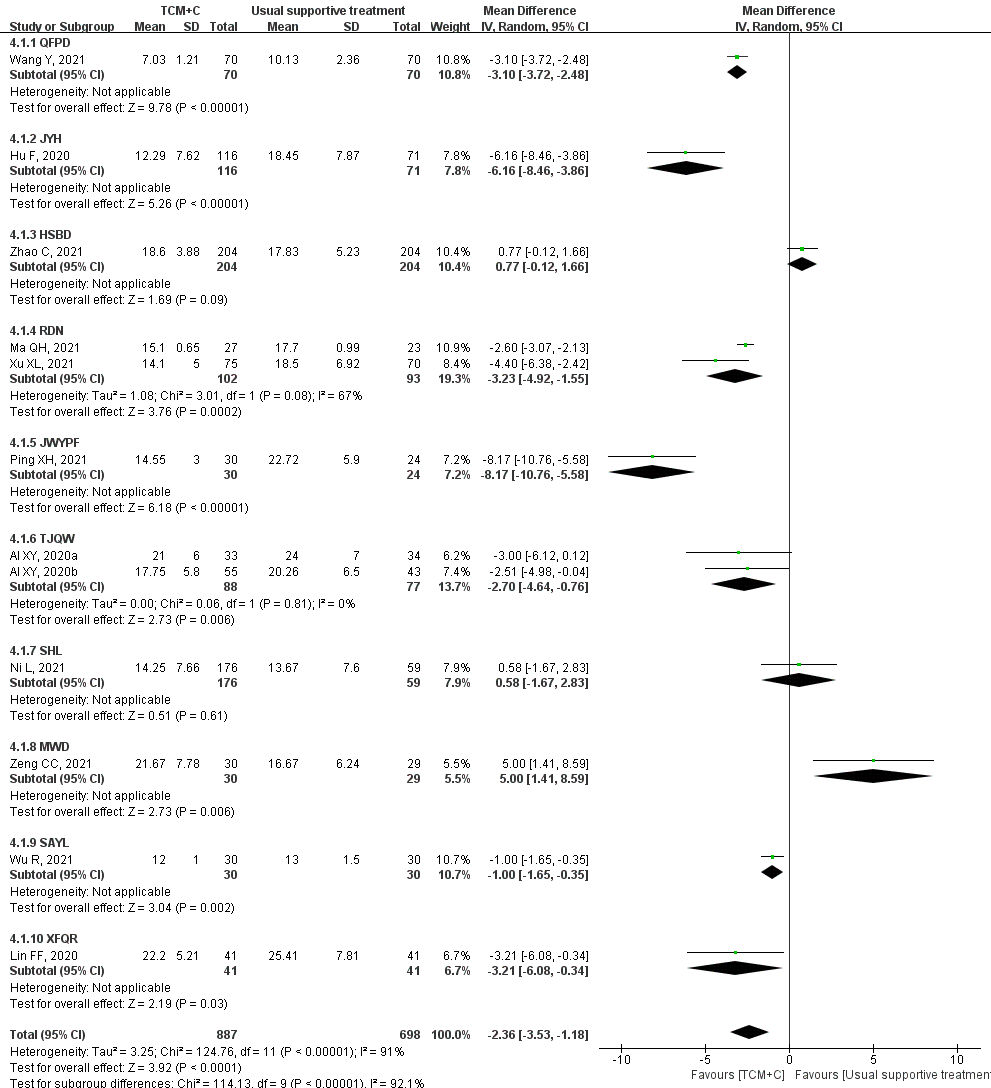


**FigureS4.2 Forest plot of length of hospital stay (OBs)**


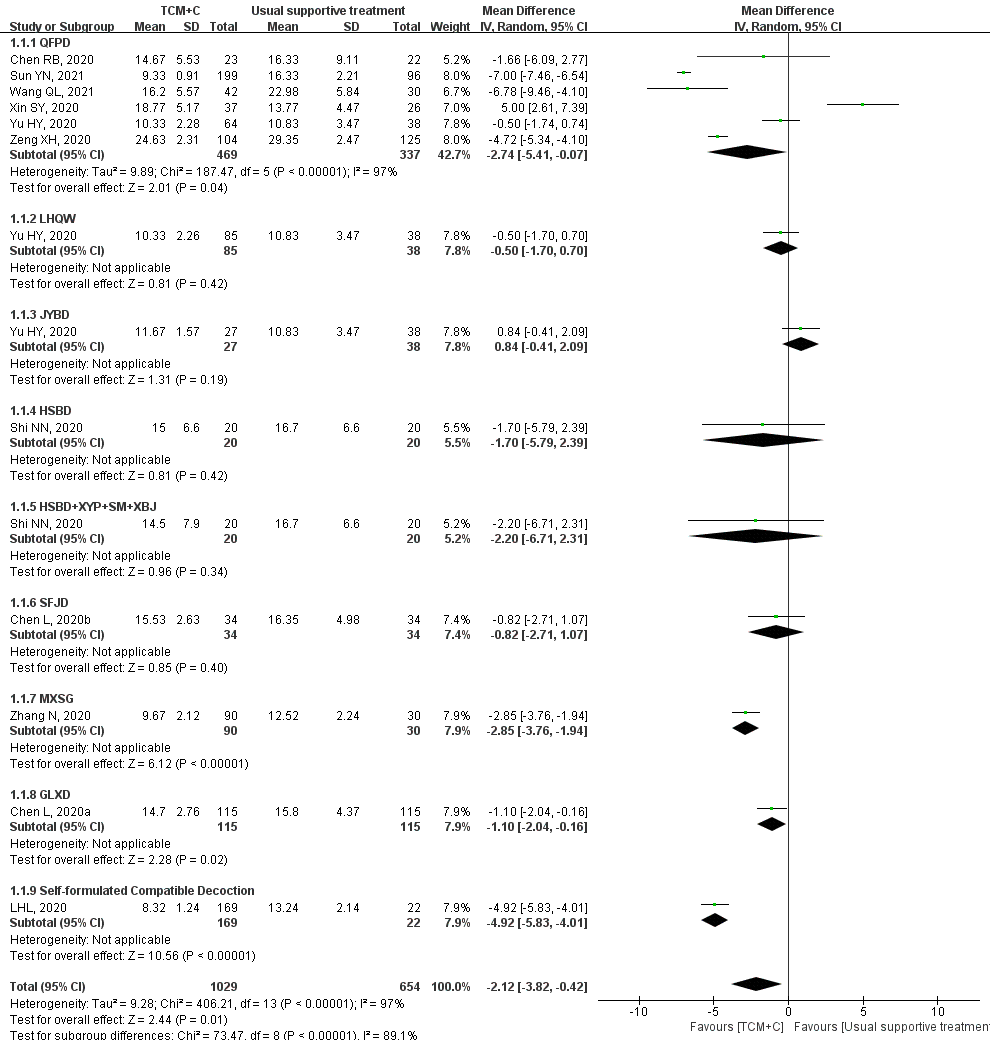


**FigureS5.1 Forest plot of time to viral clearance (RCTs)**


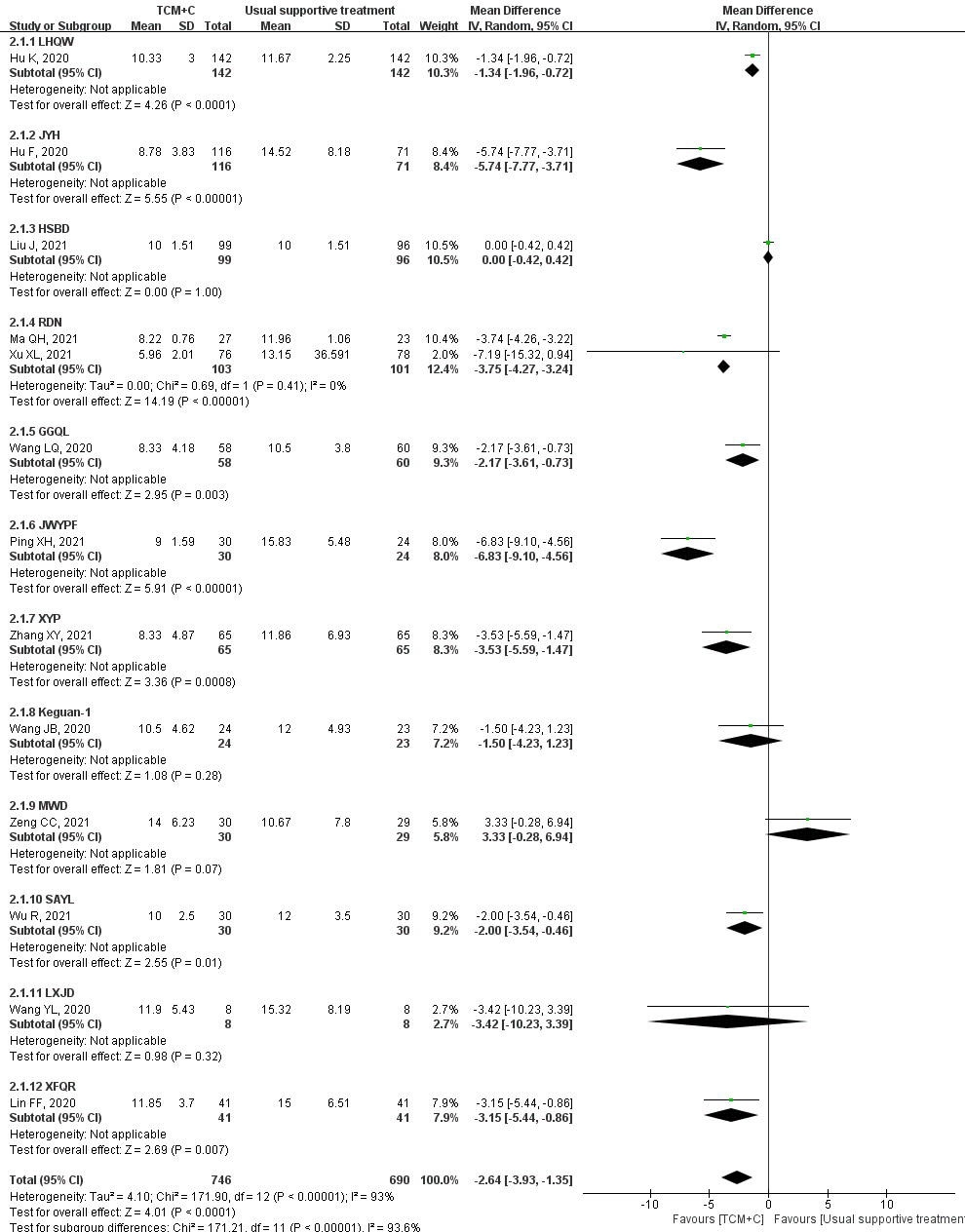


**FigureS5.2 Forest plot of** **time to viral clearance (OBs)**


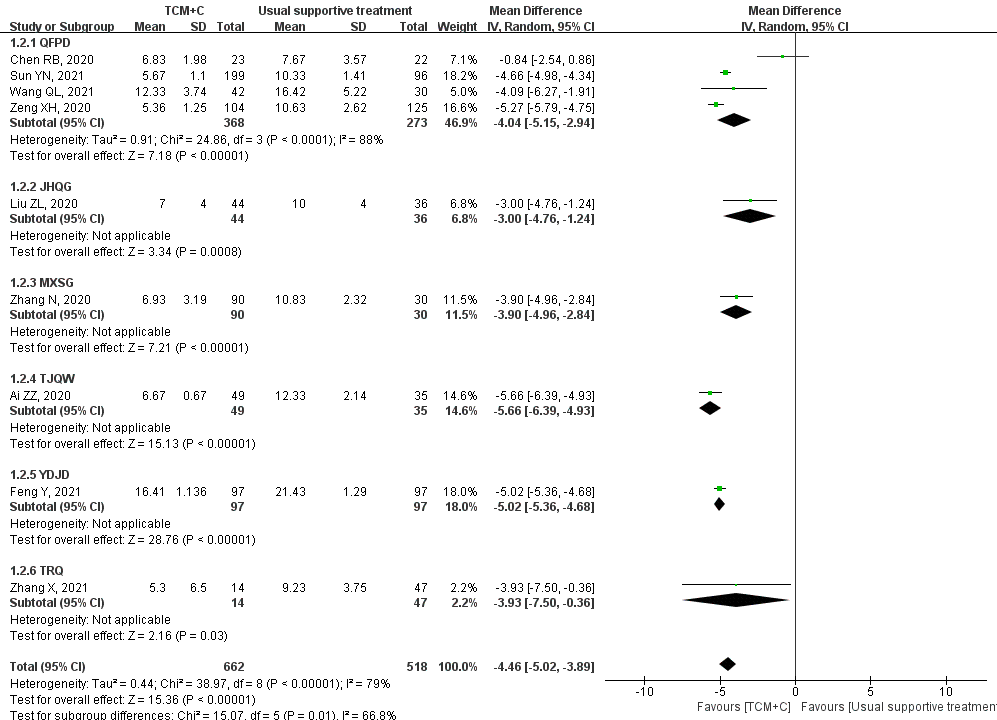


**FigureS6.1 Forest plot of rate of nucleic acid conversion (OBs)**


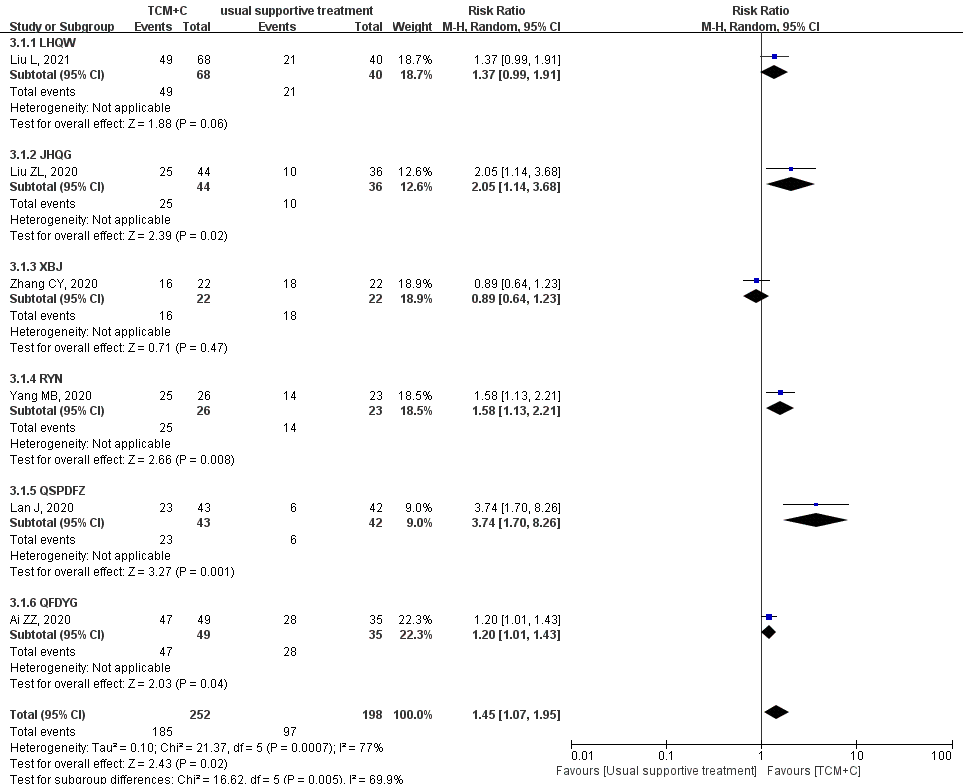


**FigureS6.2 Forest plot of rate of nucleic acid conversion (RCTs)**


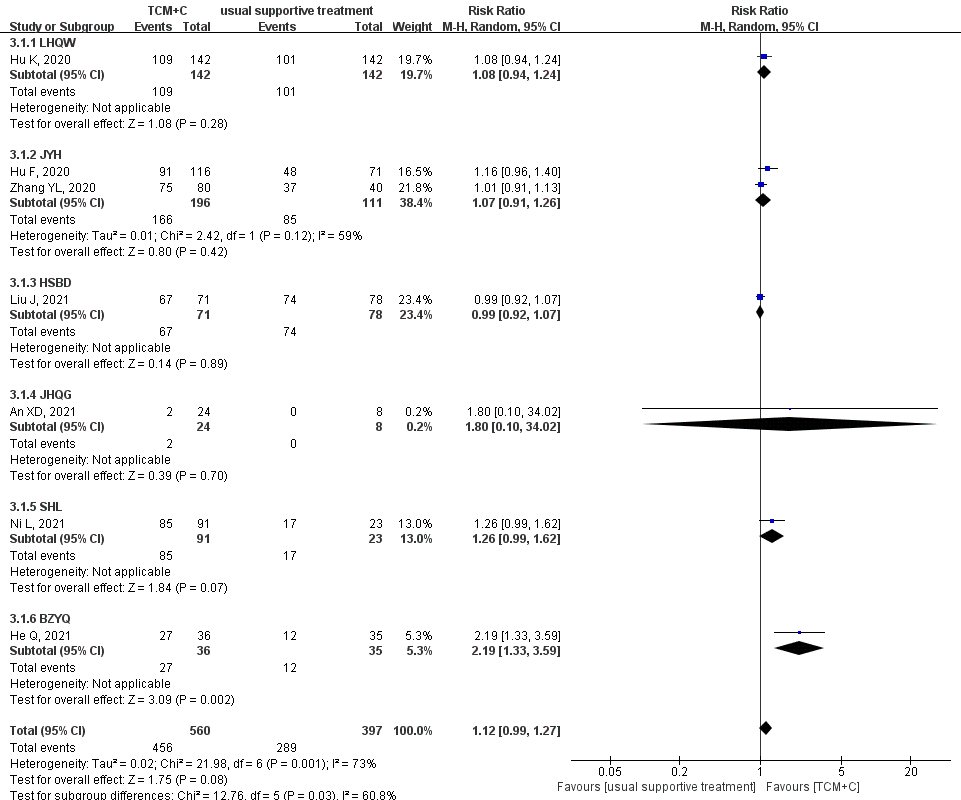


**FigureS7.1 Forest plot of rate of mortality (OBs)**


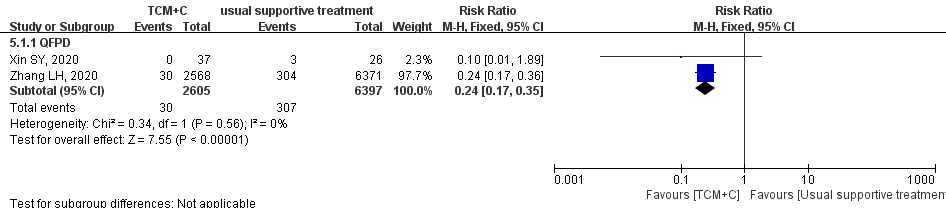


**FigureS7.2 Forest plot of rate of mortality (RCTs)**


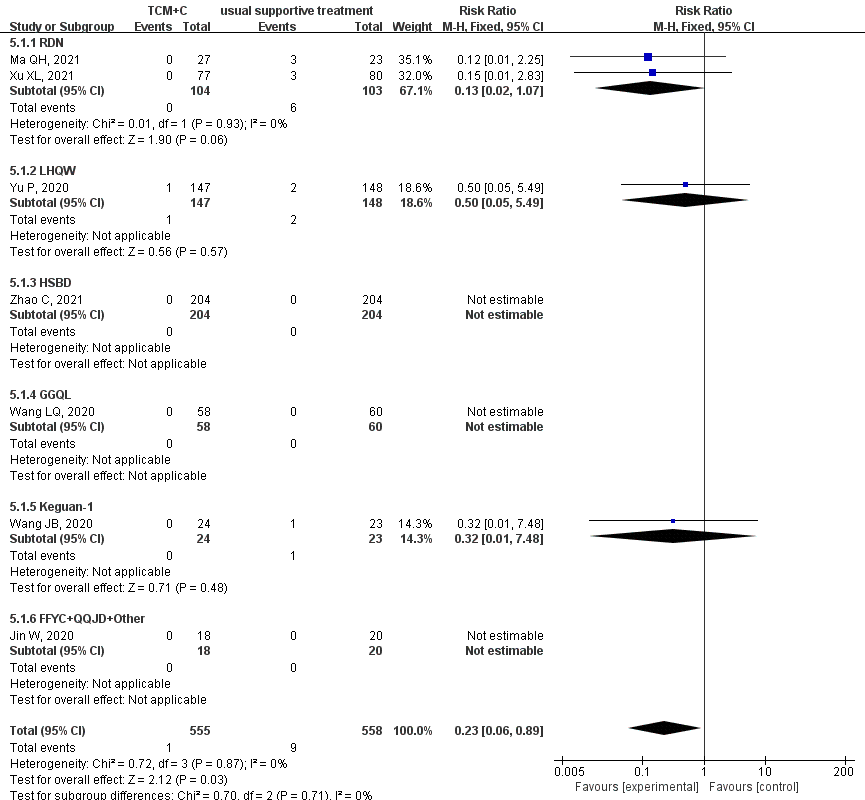


**FigureS8.1 Forest plot of rate of fever resolution (RCTs)**


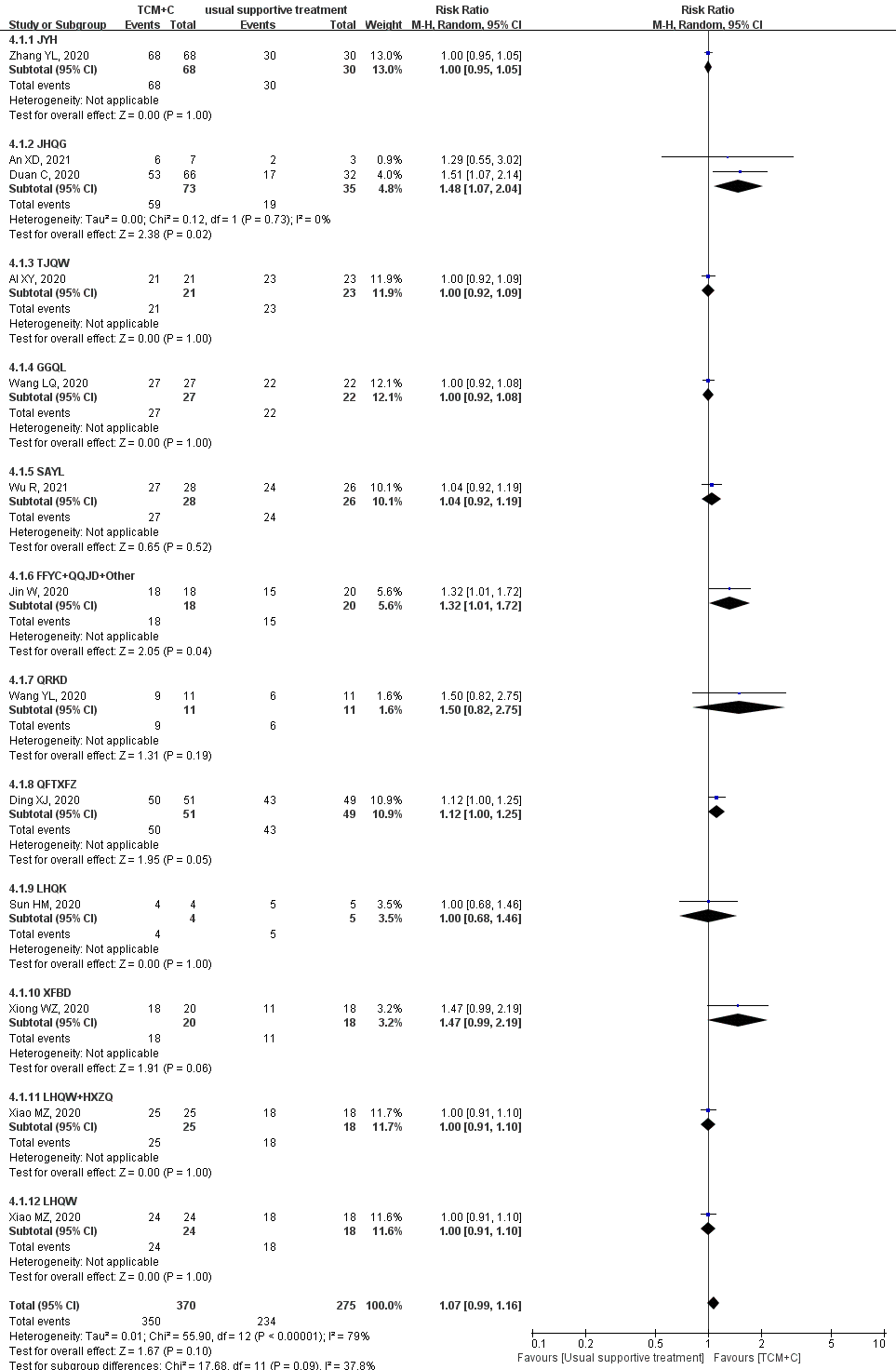


**FigureS8.2 Forest plot of rate of cough resolution (RCTs)**


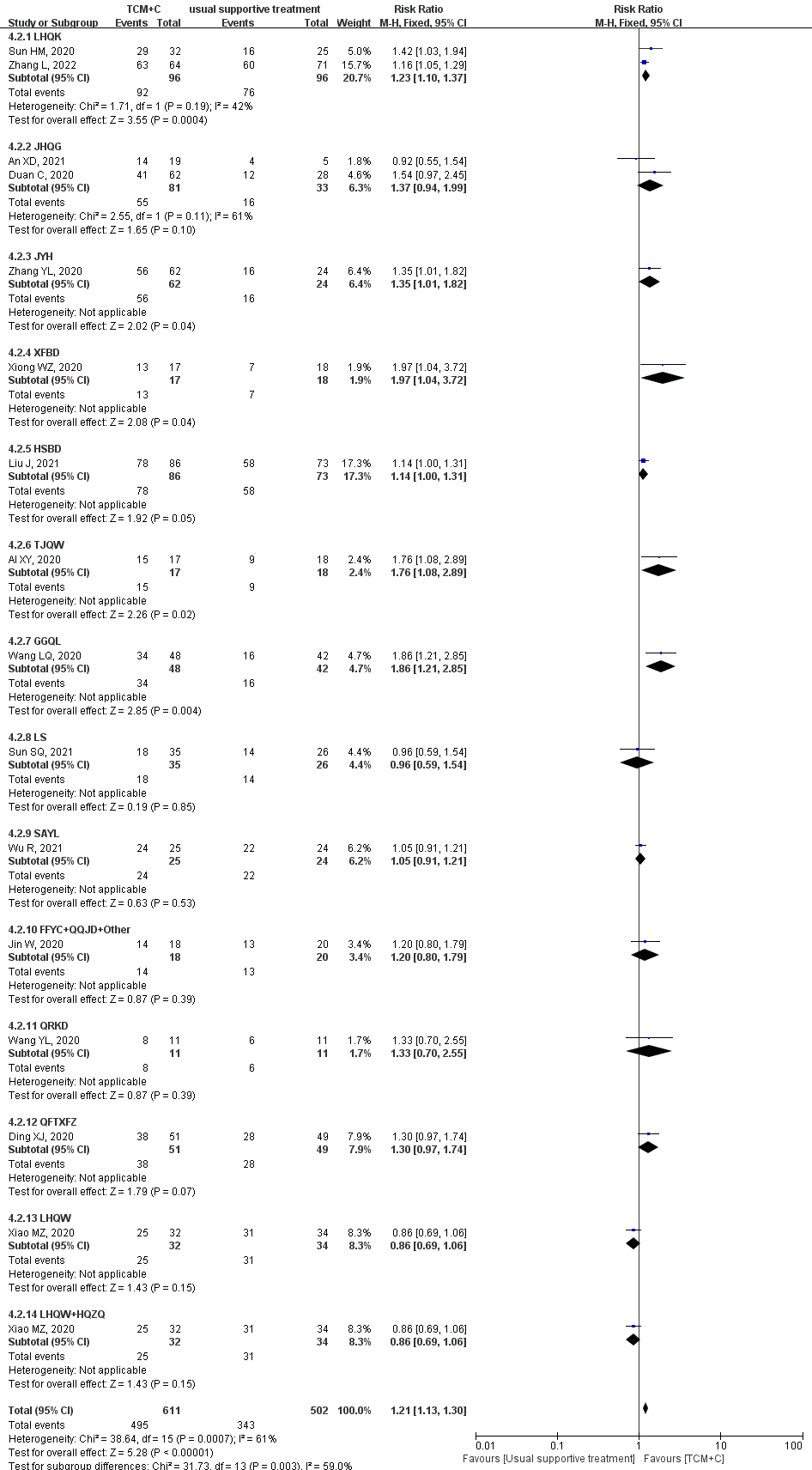


**FigureS8.3 Forest plot of rate of tiredness resolution (RCTs)**


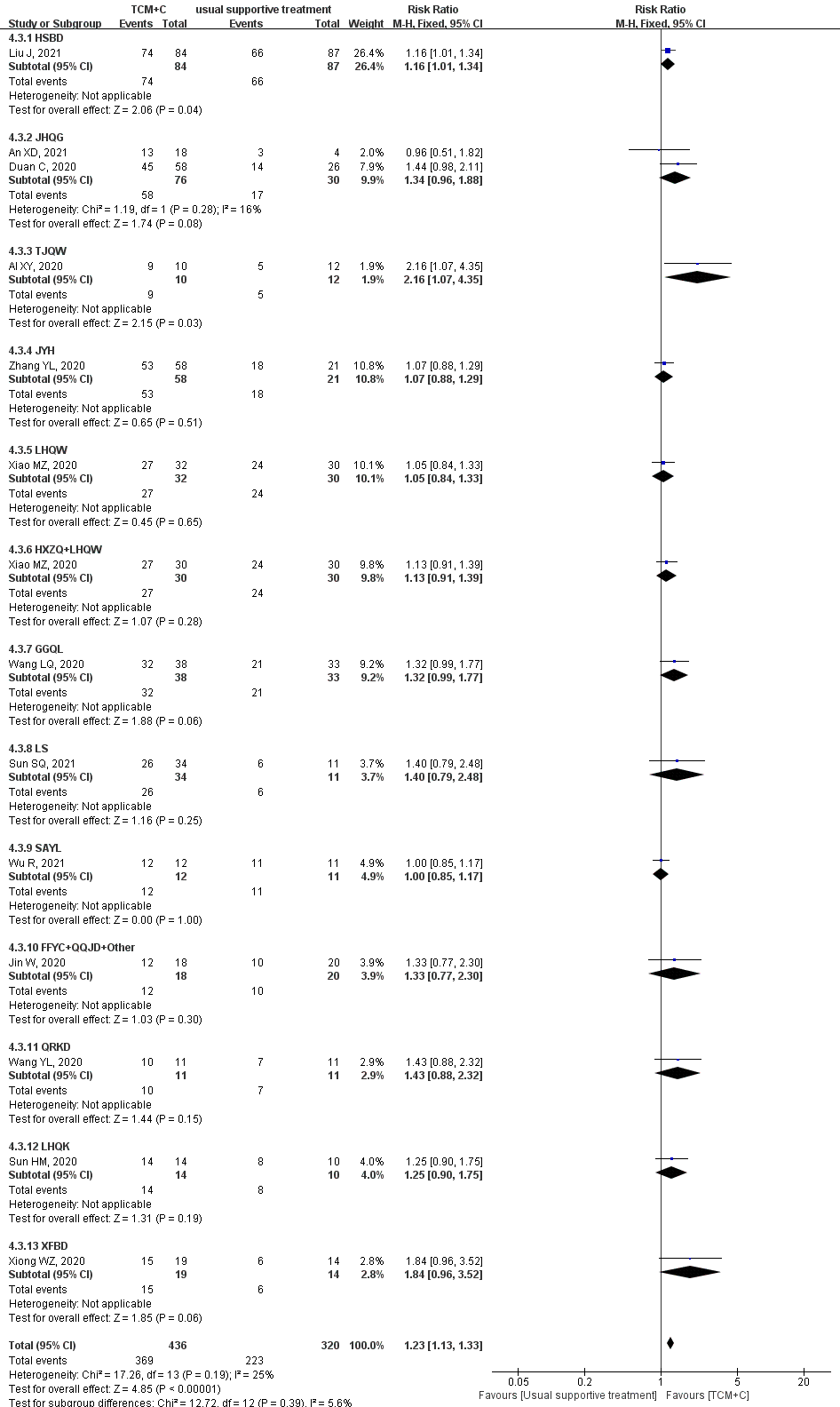


**FigureS8.4 Forest plot of rate of expectoration resolution (RCTs)**


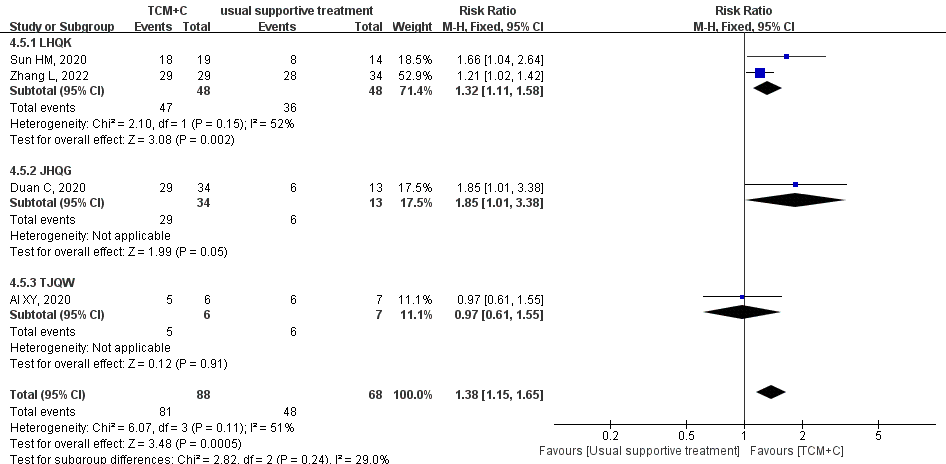


**FigureS8.5 Forest plot of rate of loss of appetite resolution (RCTs)**


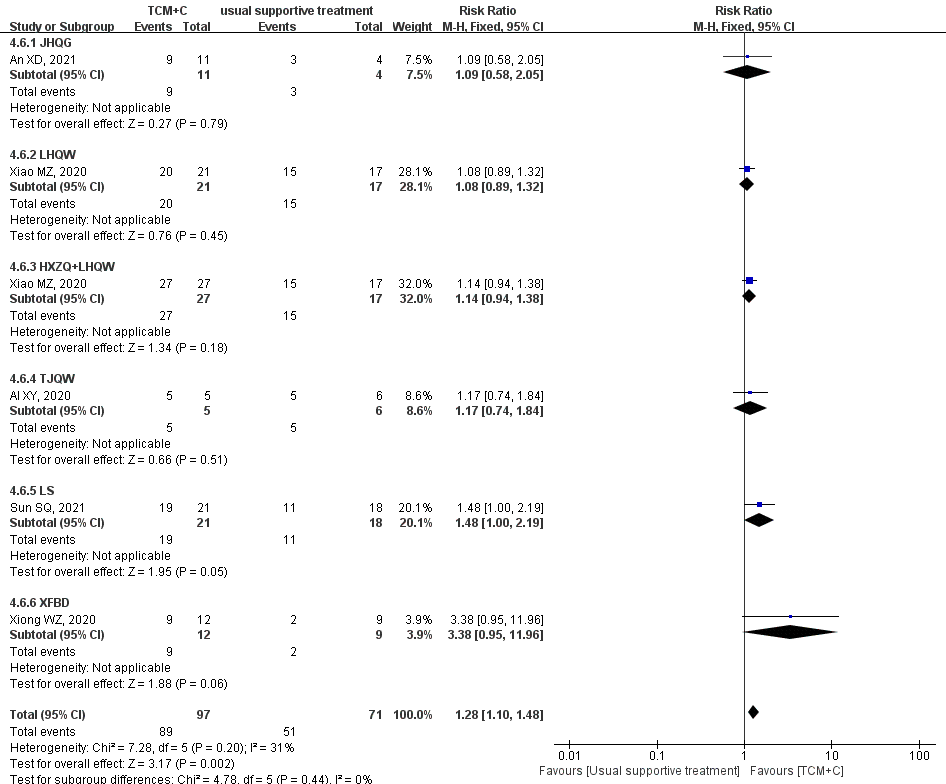


**FigureS8.6 Forest plot of rate of shortness of breath resolution (RCTs)**


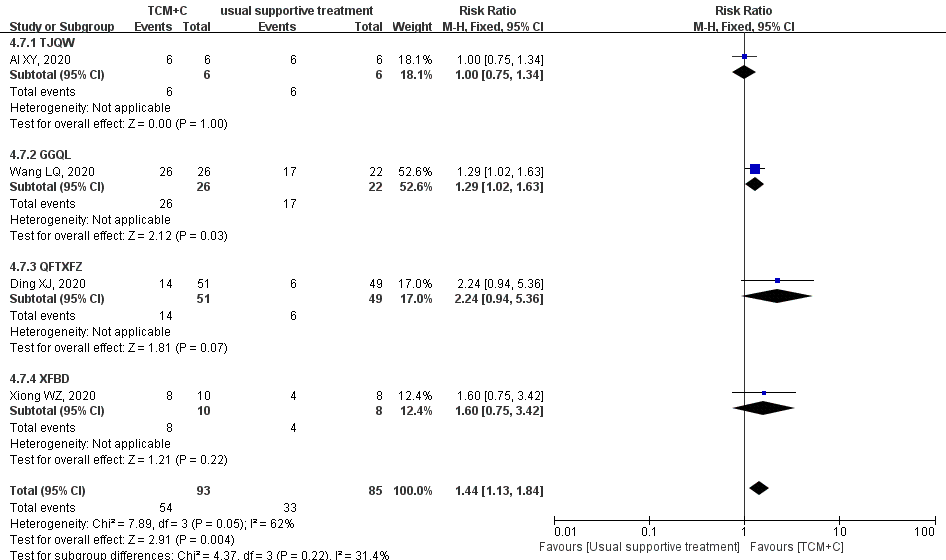


**FigureS8.7 Forest plot of rate of chest tightness resolution (RCTs)**


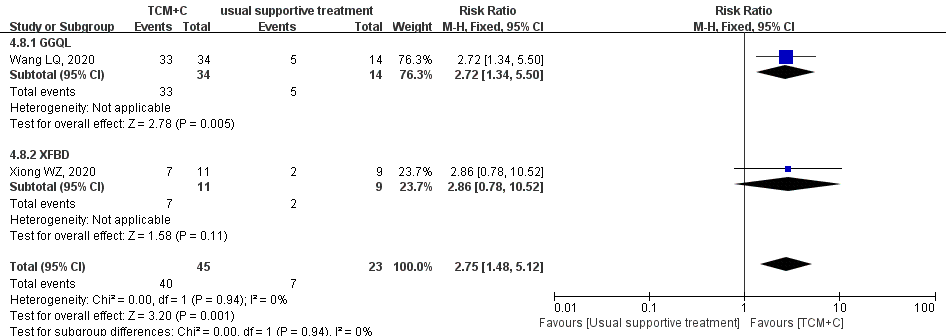


**FigureS8.8 Forest plot of rate of chest tightness and shortness of breath resolution (RCTs)**


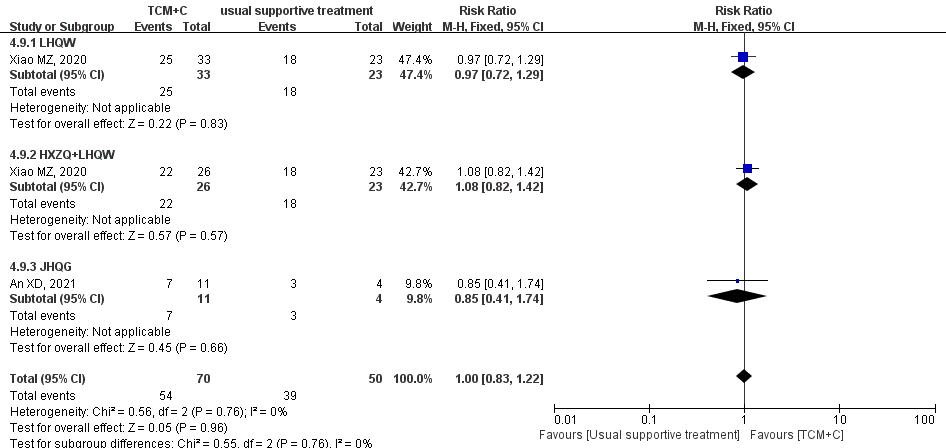


**FigureS8.9 Forest plot of rate of diarrhea resolution (RCTs)**


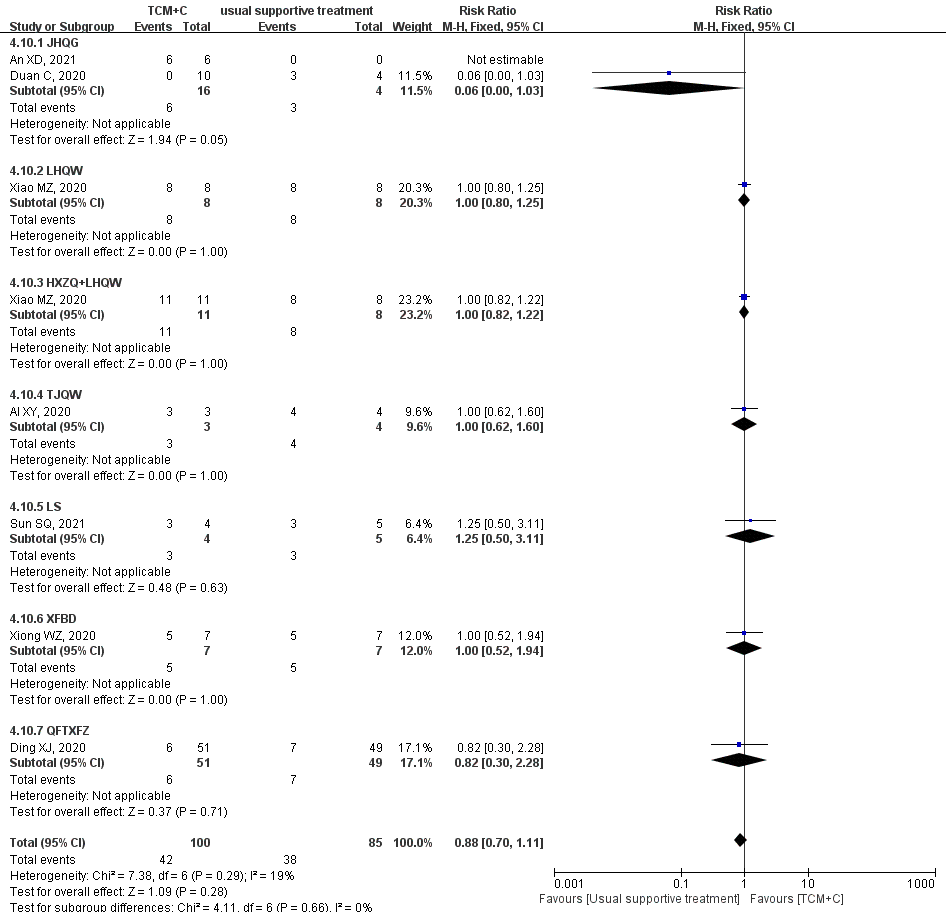


**FigureS8.10 Forest plot of rate of CT improvement resolution (RCTs)**


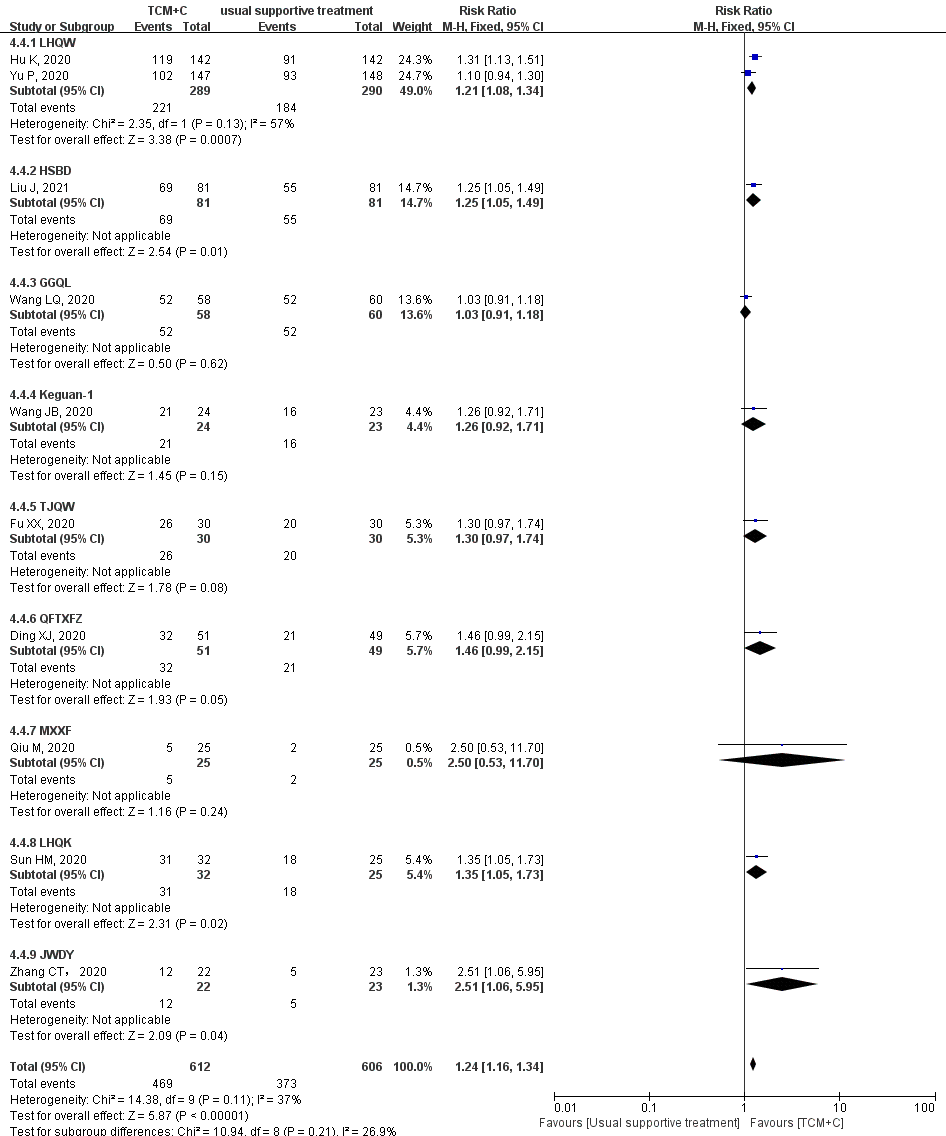


**FigureS9.1 Forest plot of rate of fever resolution (OBs)**


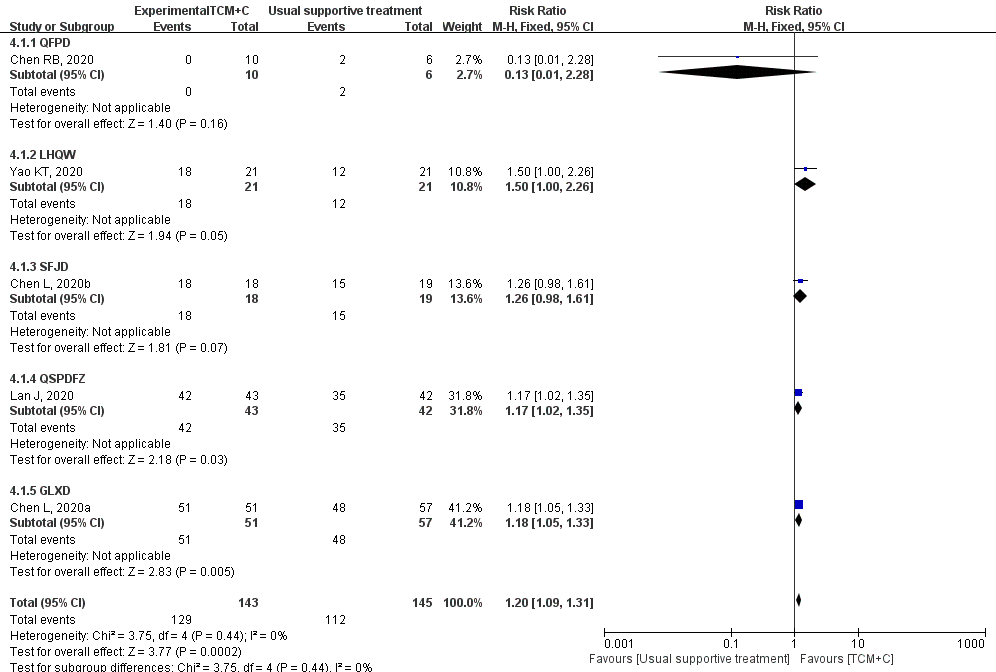


**FigureS9.2 Forest plot of rate of cough resolution (OBs)**


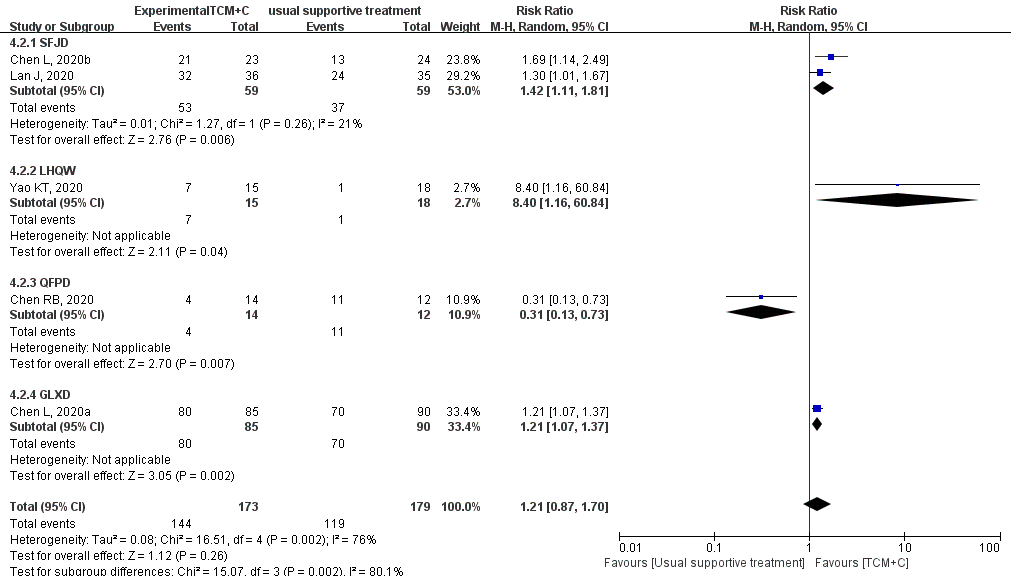


**FigureS9.3 Forest plot of rate of tiredness resolution (OBs)**


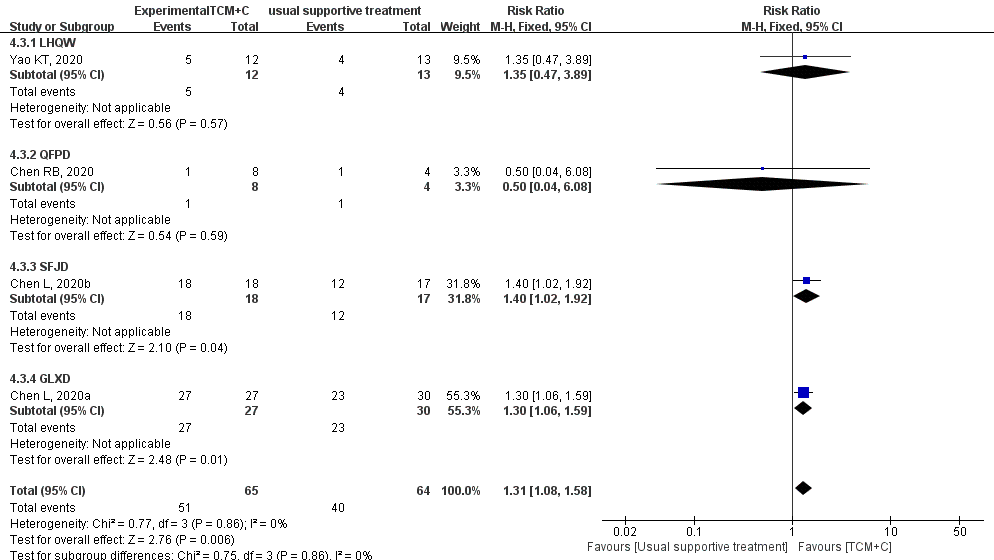


**FigureS9.4 Forest plot of rate of expectoration resolution (OBs)**


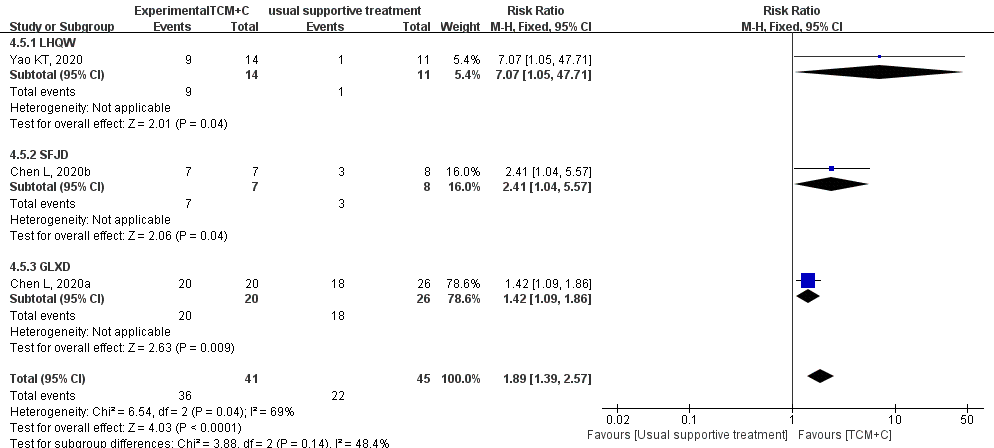


**FigureS9.5 Forest plot of rate of loss of appetite resolution (OBs)**


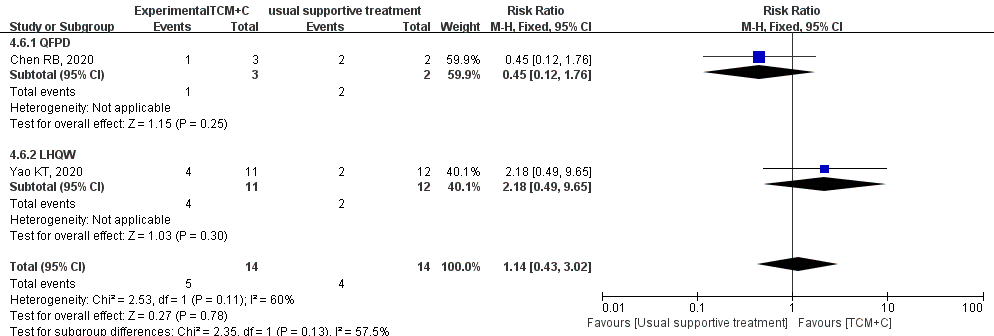


**FigureS9.6 Forest plot of rate of shortness of breath resolution (OBs)**


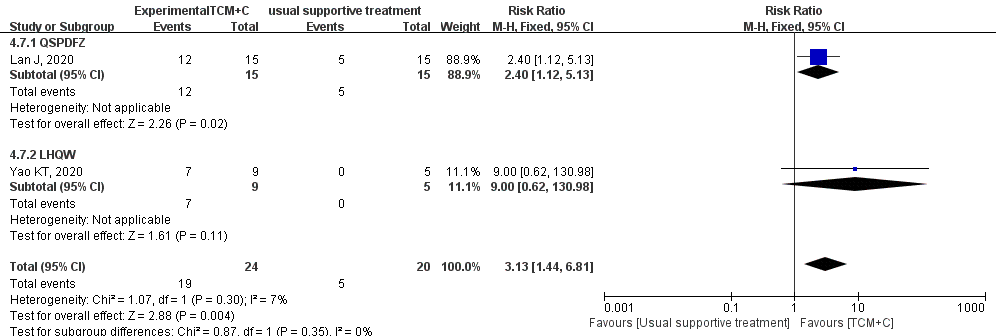


**FigureS9.7 Forest plot of rate of chest tightness resolution (OBs)**


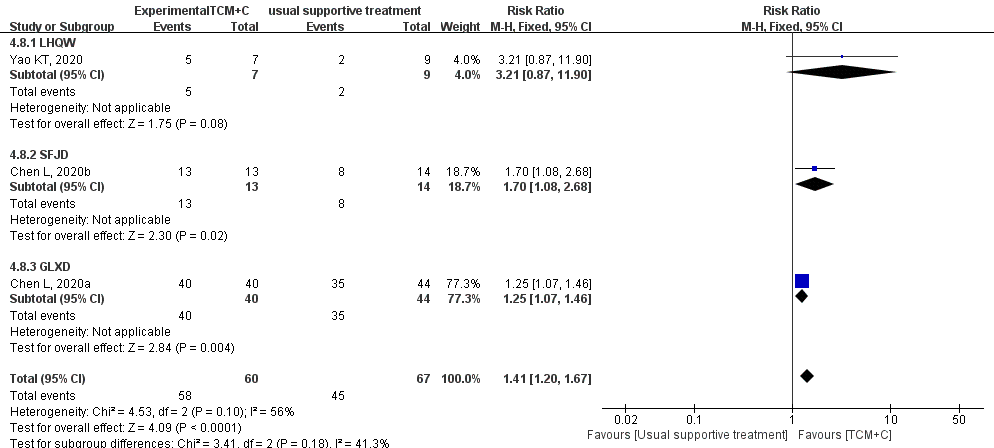


**FigureS9.8 Forest plot of rate of diarrhea resolution (OBs)**


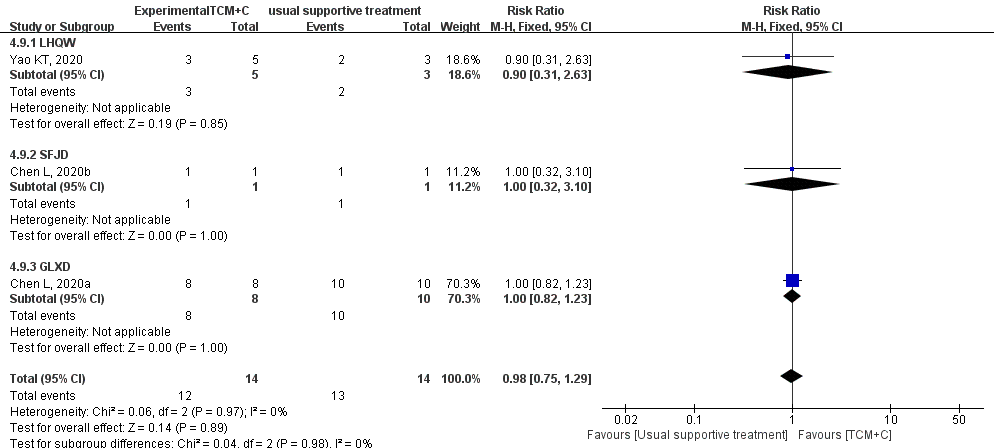


**FigureS9.9 Forest plot of rate of CT improvement (OBs)**


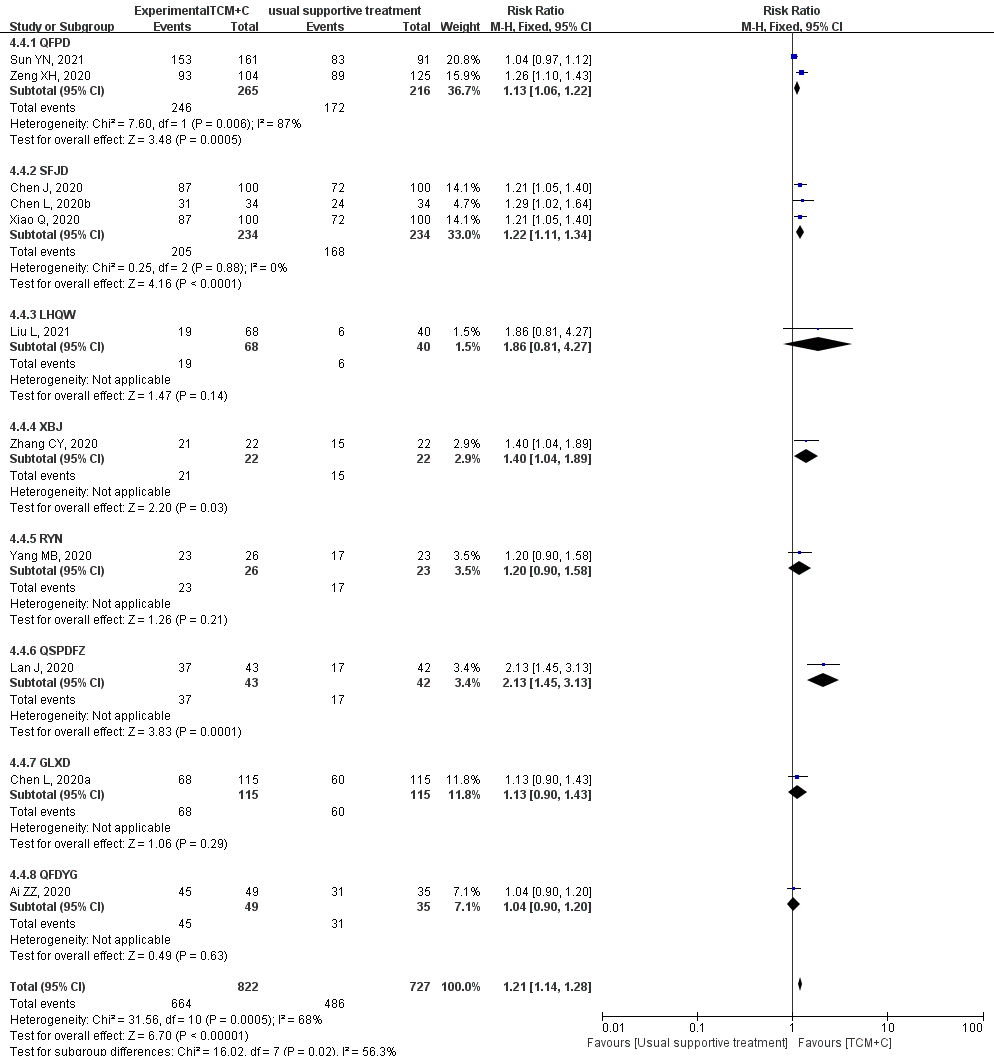


**FigureS10 Publication bias**

**10.1 Rate of conversion to severe cases-RCTs**


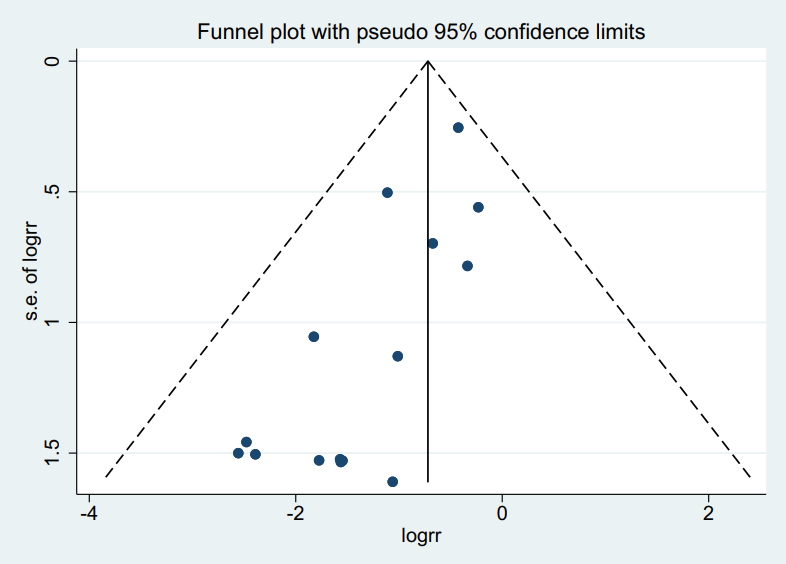


(a)


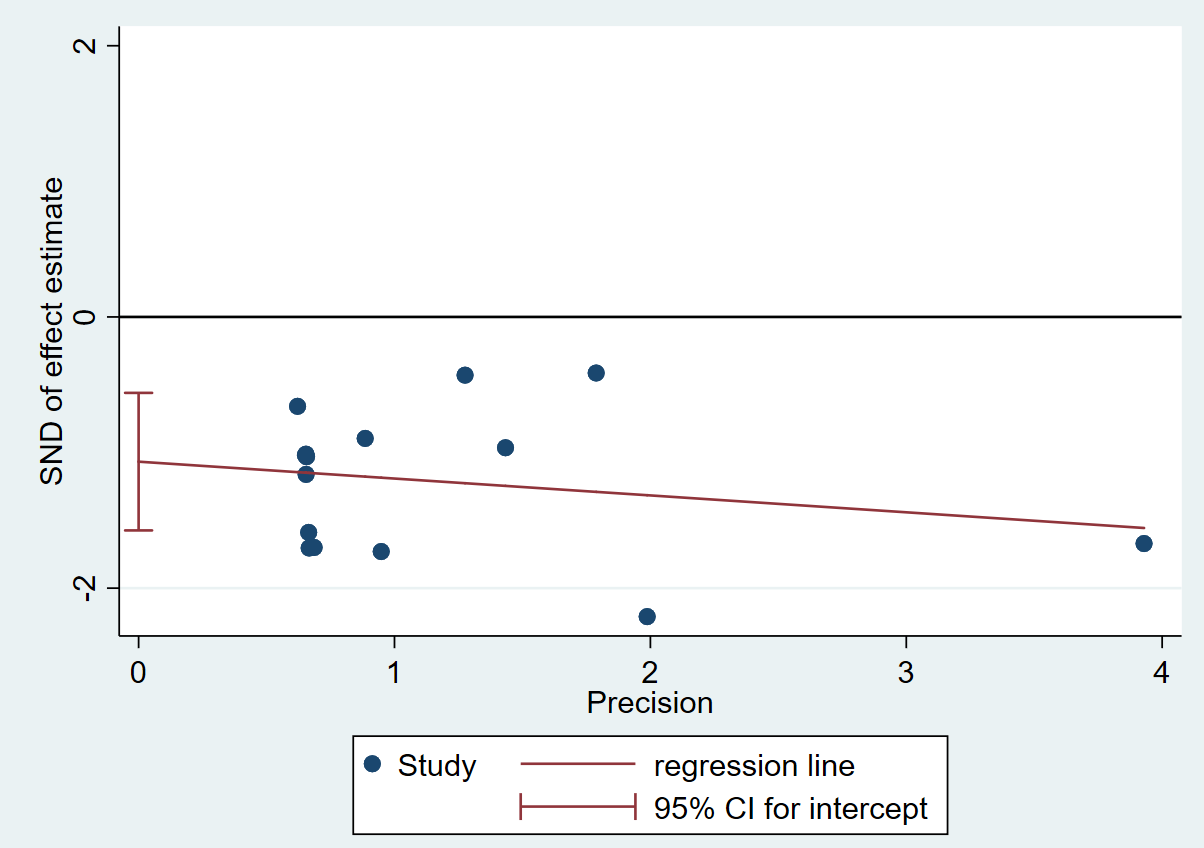


(b)


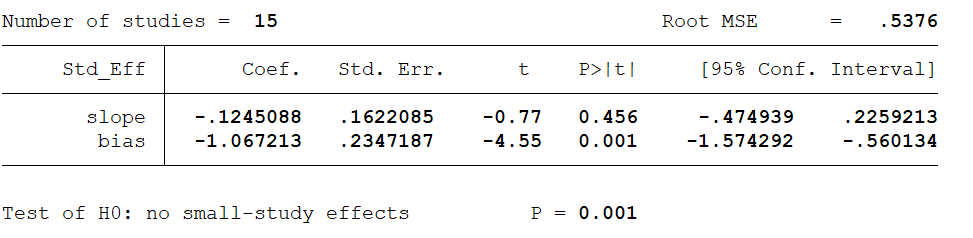


(c)

**10.2 Rate of conversion to severe cases-OBs**


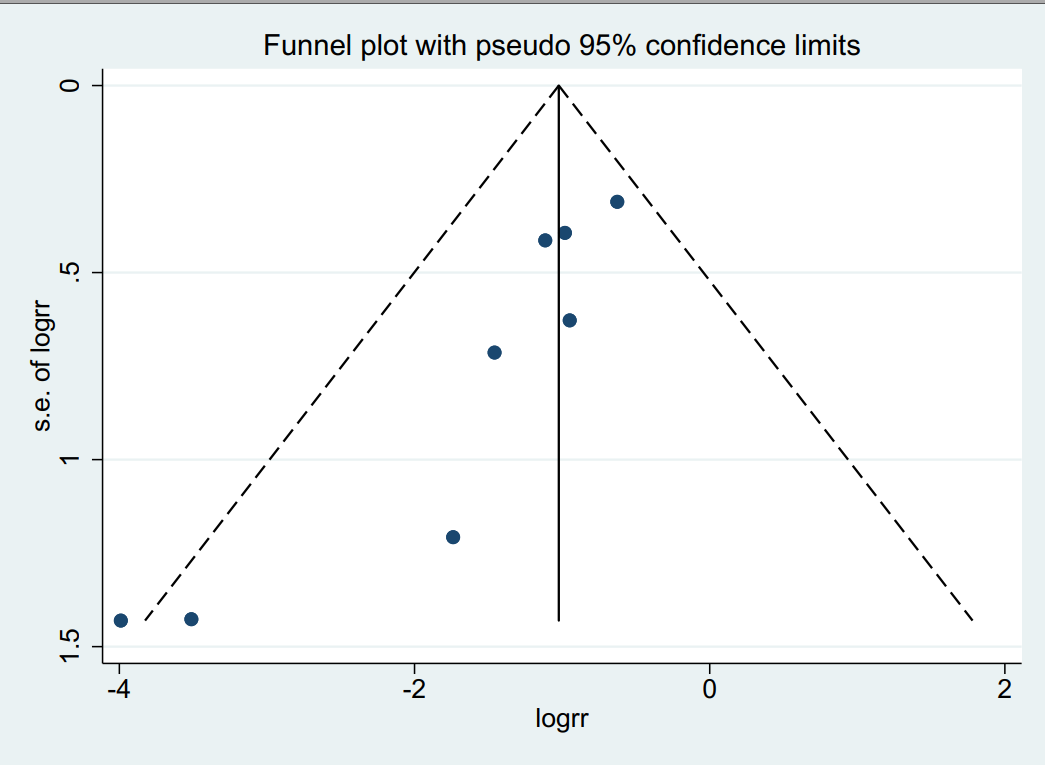


(a)


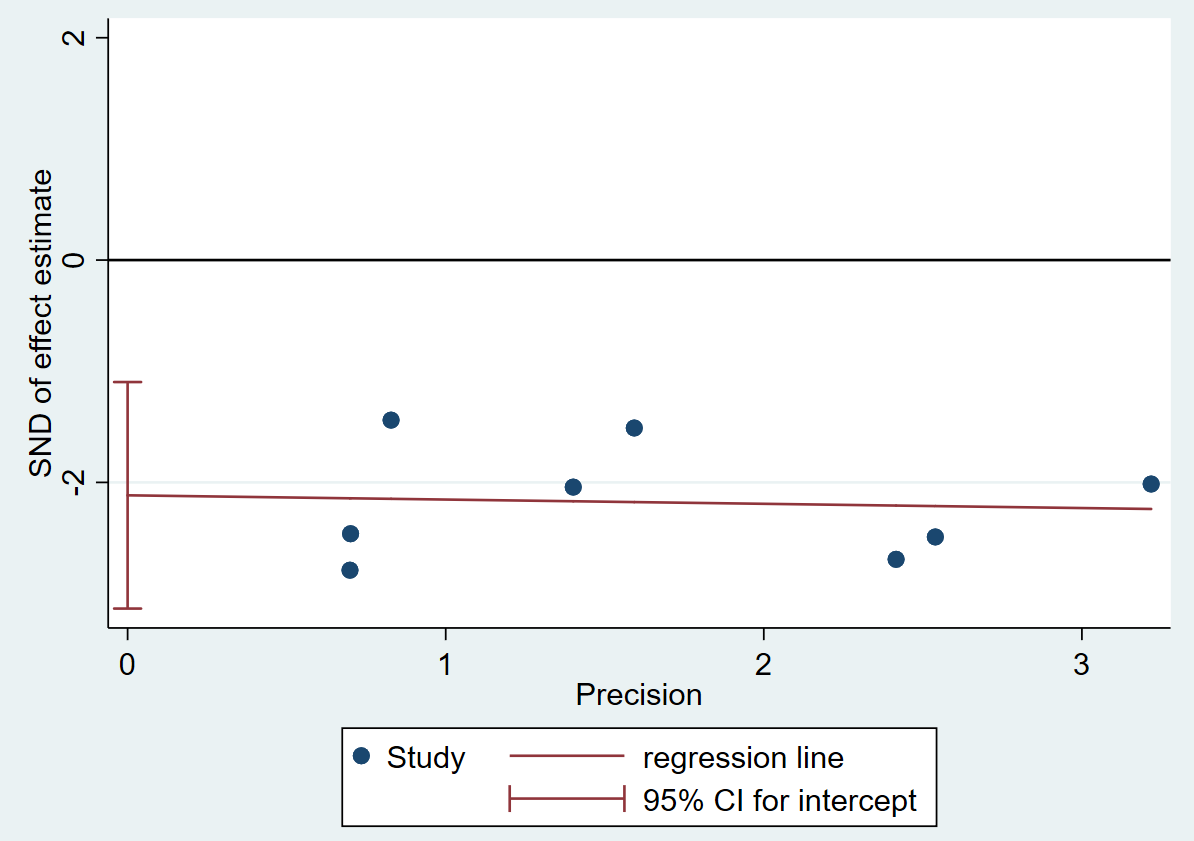


(b)


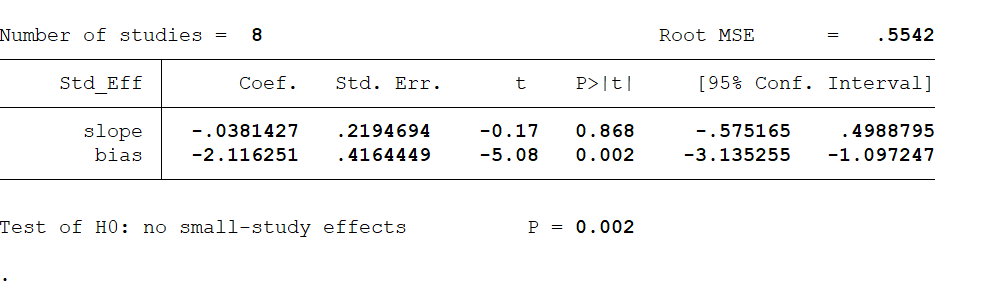


(c)

**10.3 Length of hospital stay-RCTs**


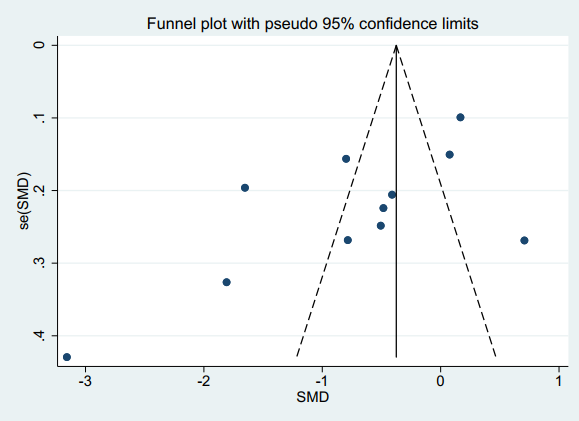


(a)


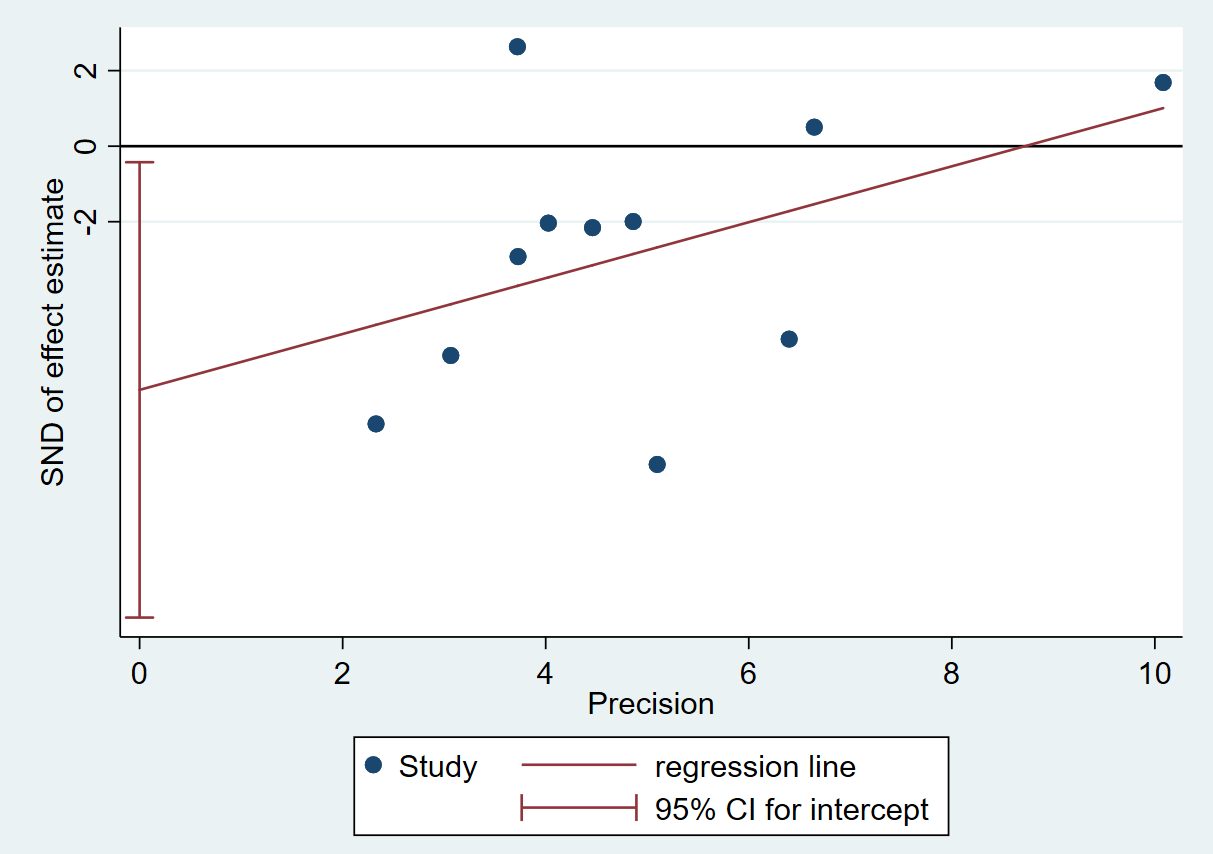


(b)


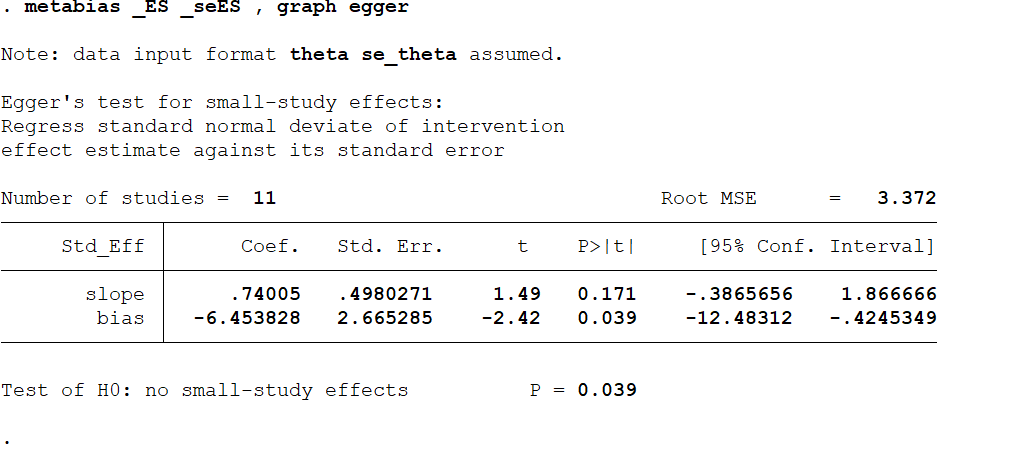


(c)

**10.4** **Length of hospital stay-OBs**


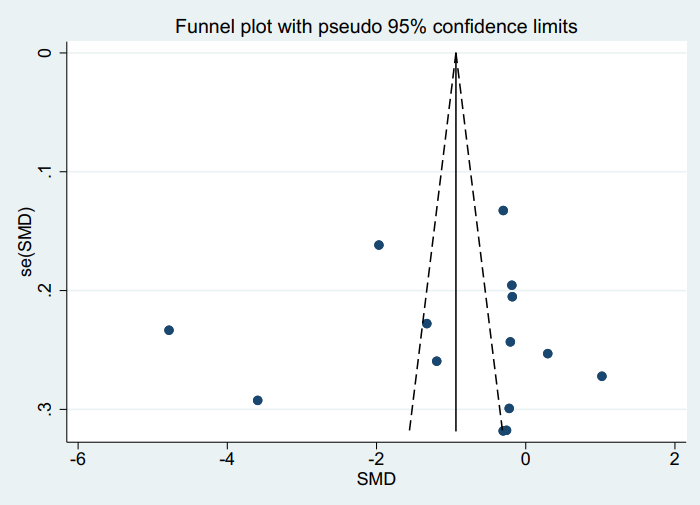


(a)


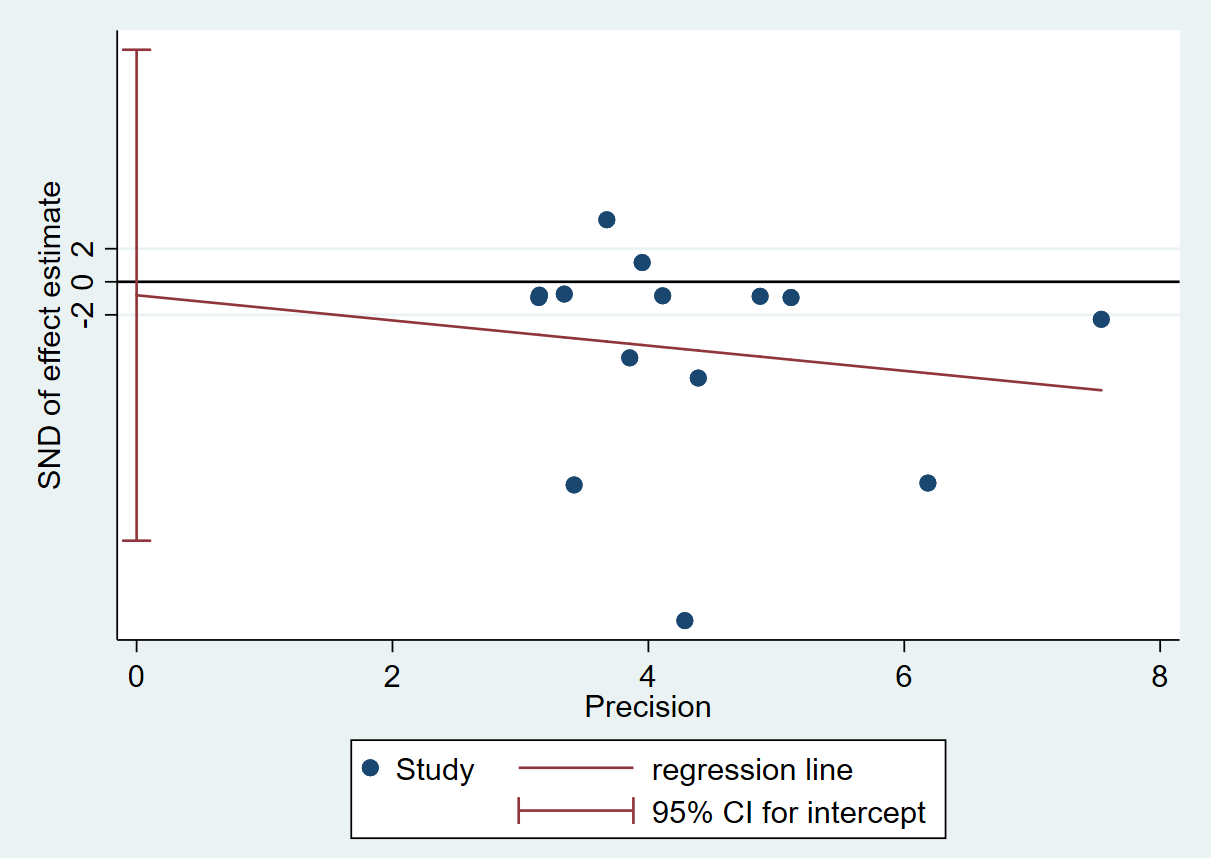


(b)


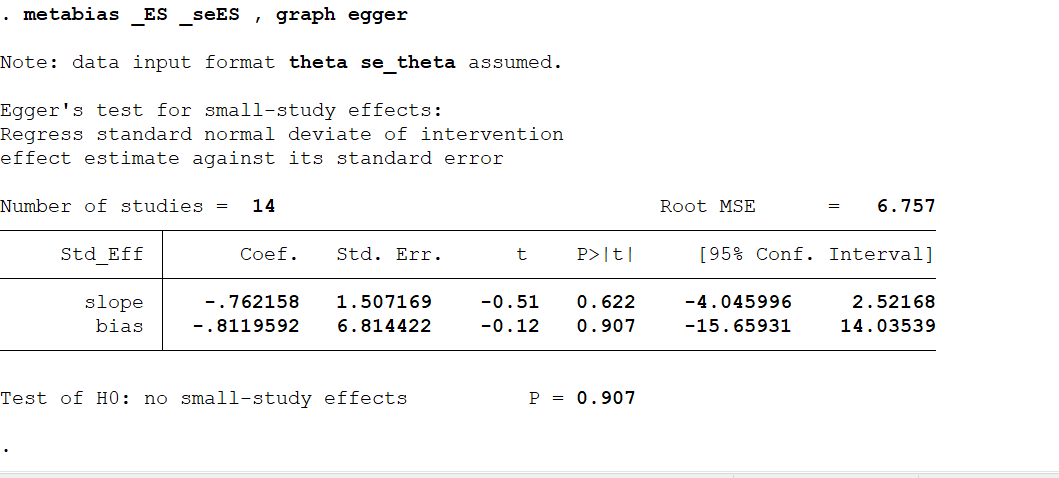


(c)

**10.5 Time to viral clearance-RCTs**


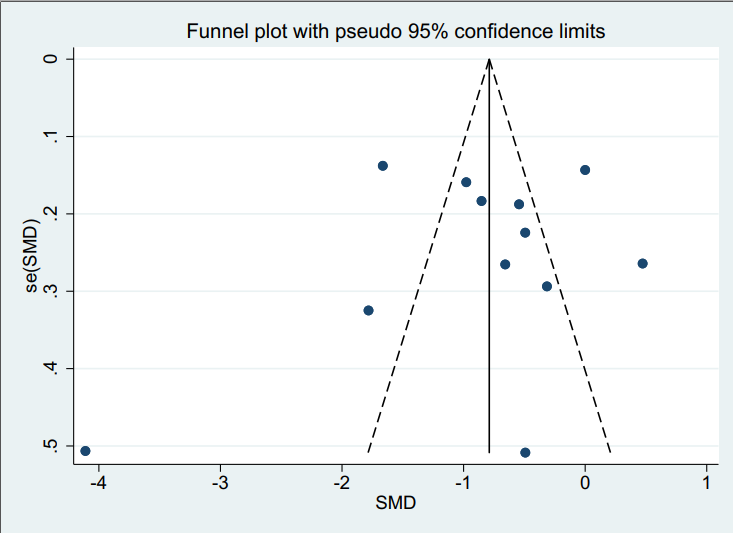


(a)


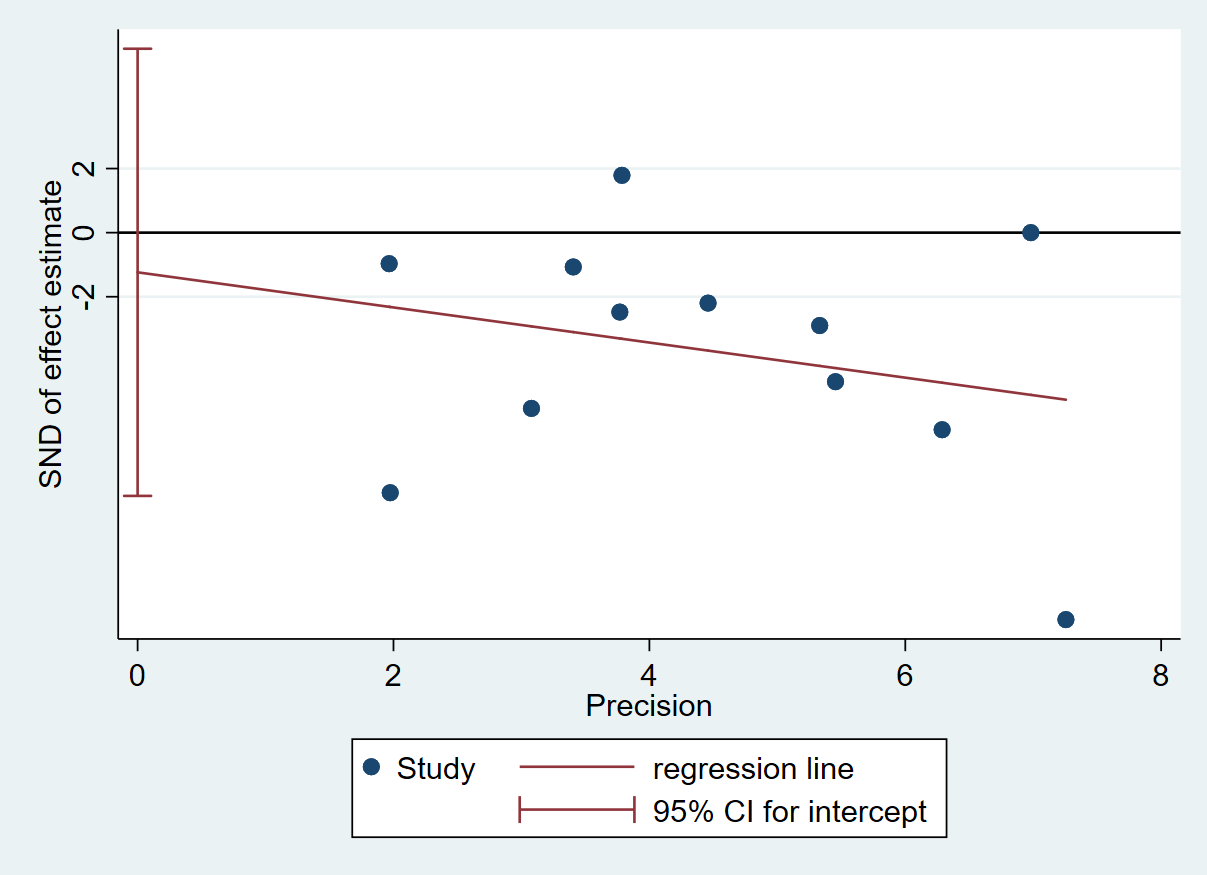


(b)


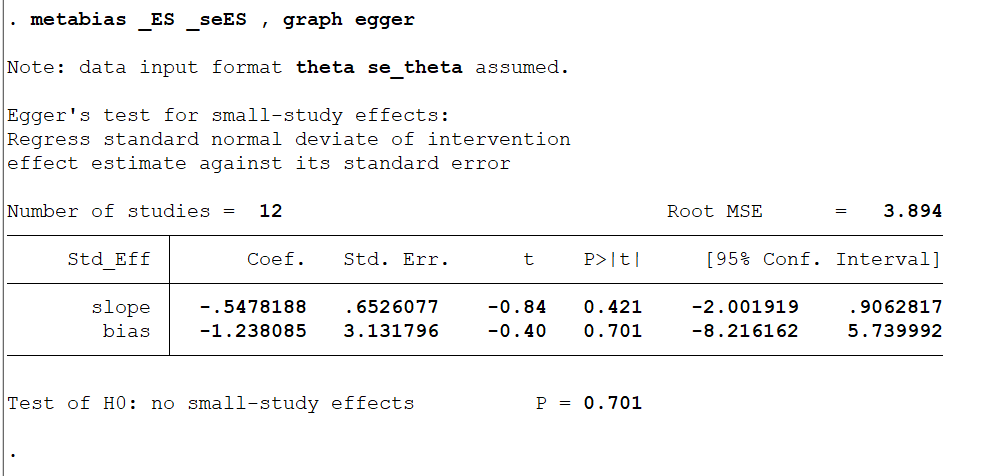


(c)

**Figure S11** **Meta-regression analysis**

**11.1 Length of hospital stay-RCTs**


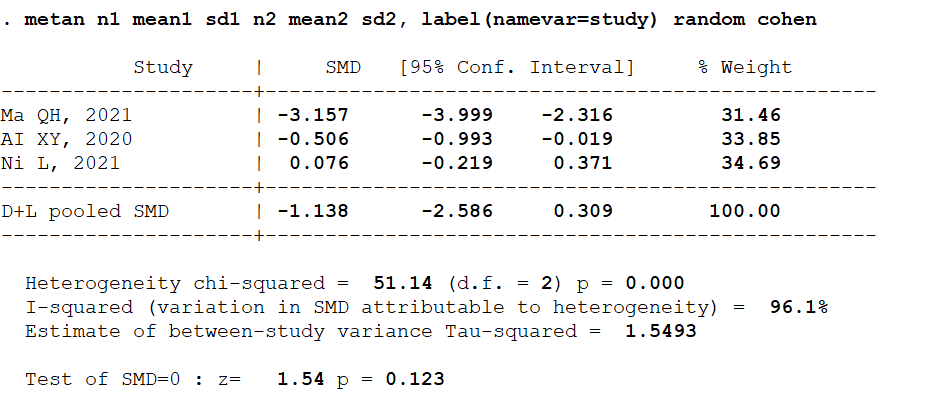


(a)


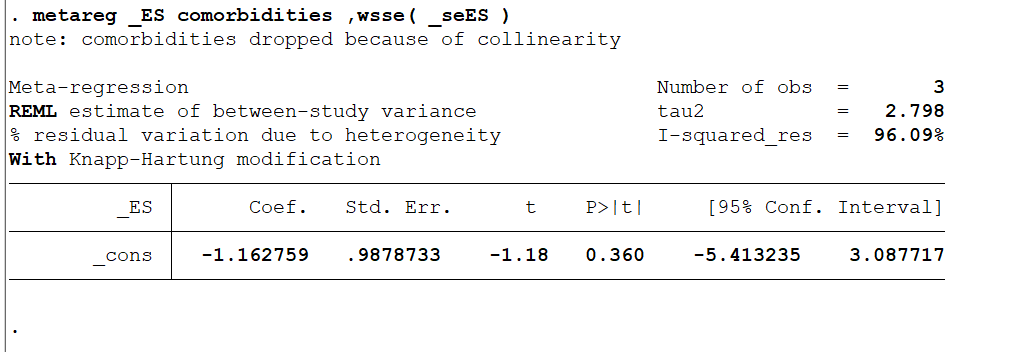


(b)


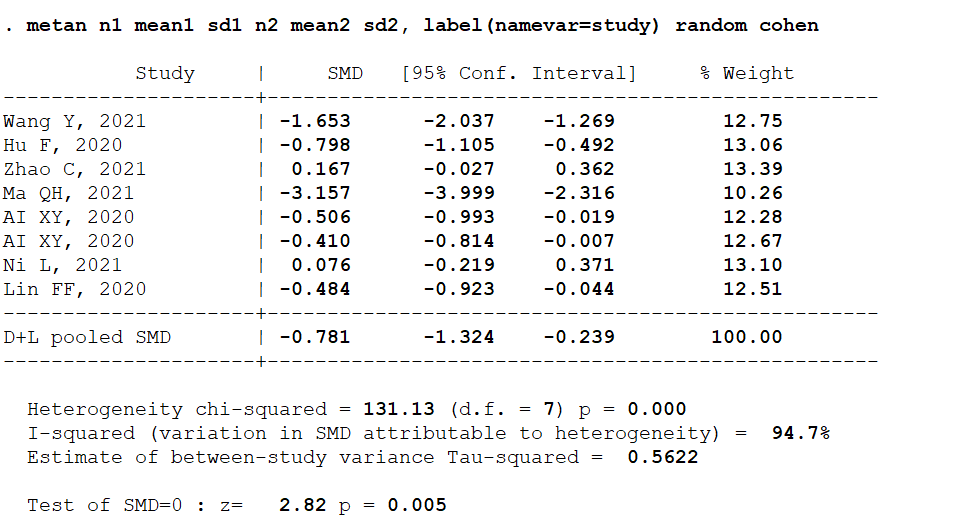


(c)


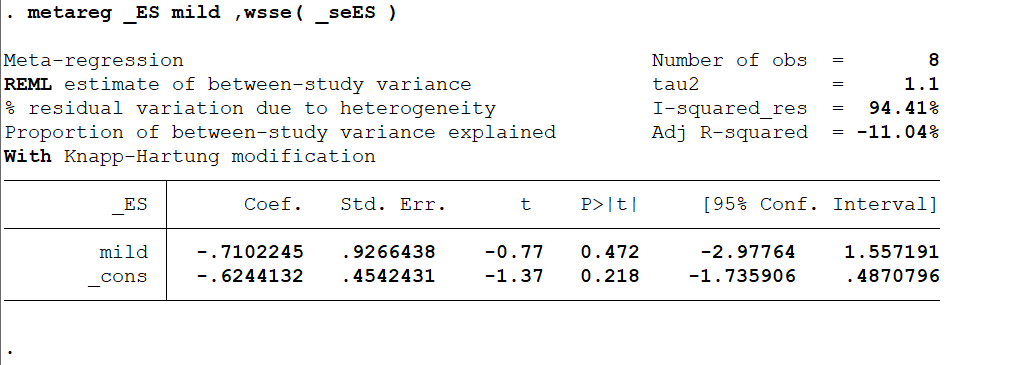


(d)


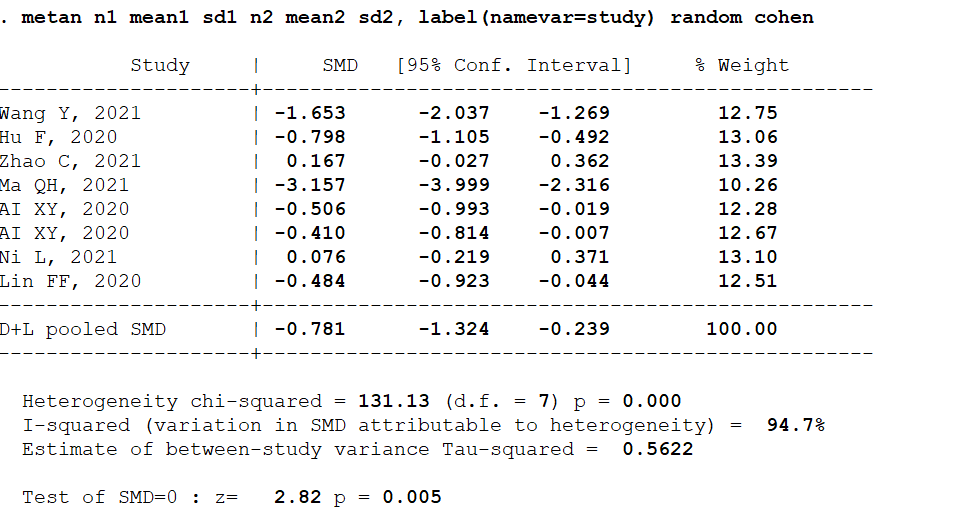


(e)


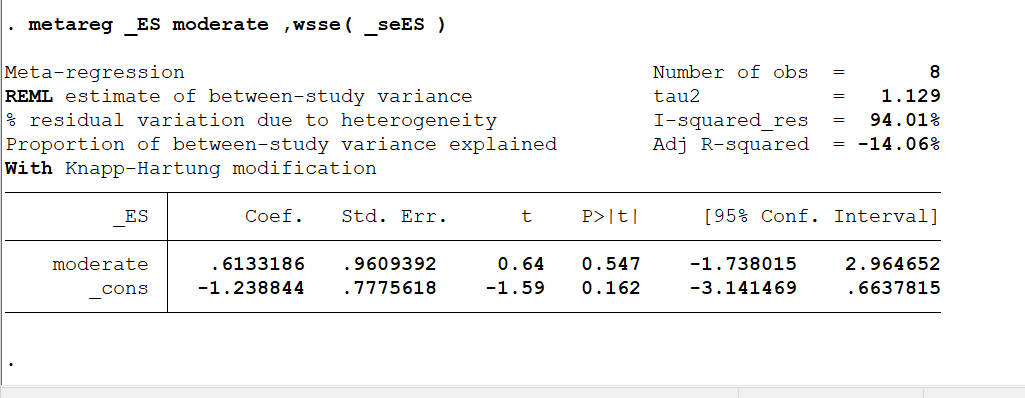


(f)

**11.2 Length of hospital stay-OBs**


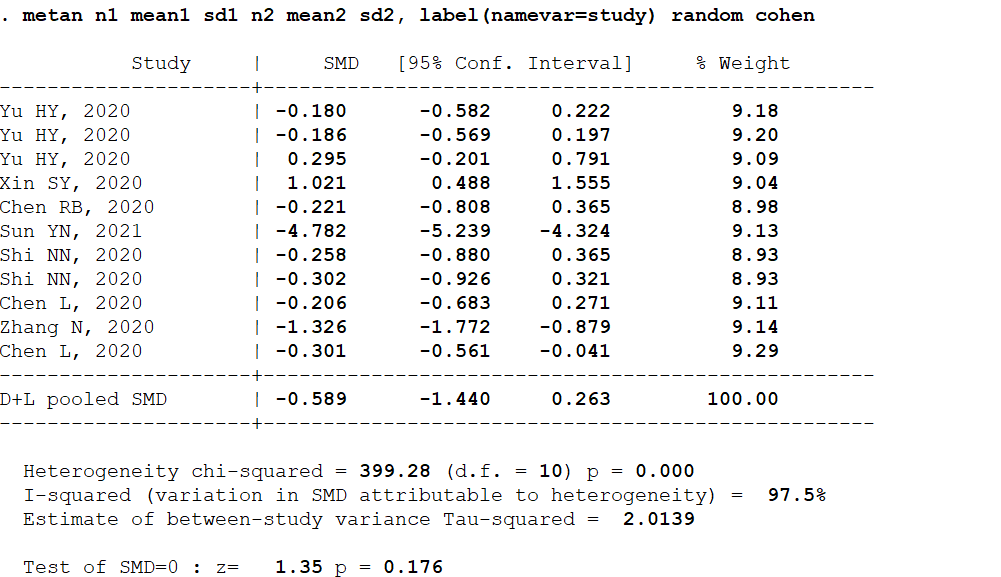


(a)


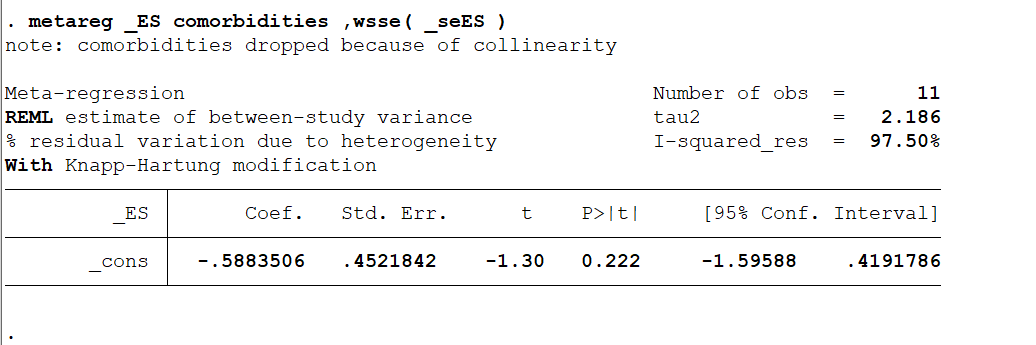


(b)


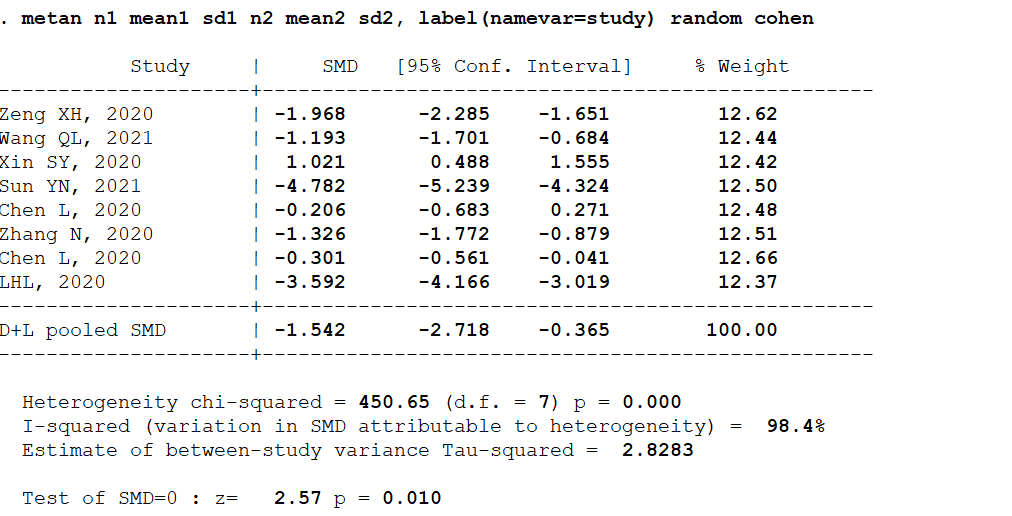


(c)


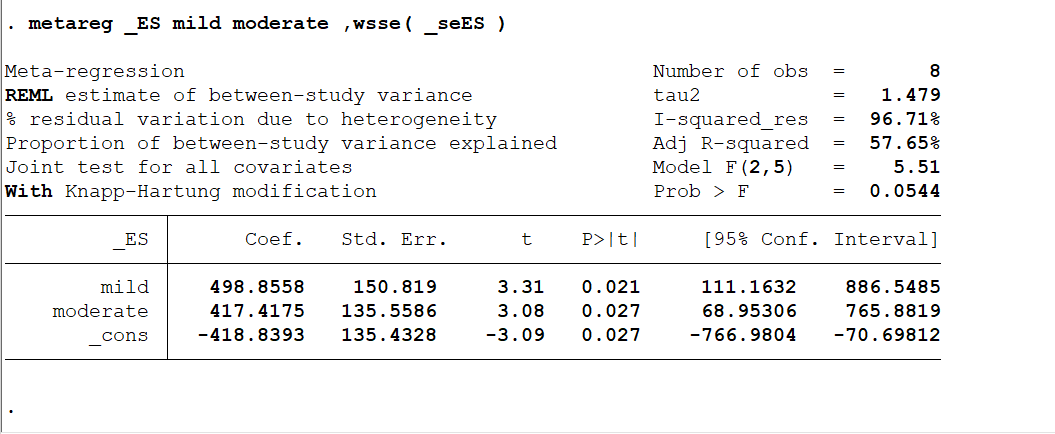


(d)

**11.3 Time to viral clearance-RCTs**


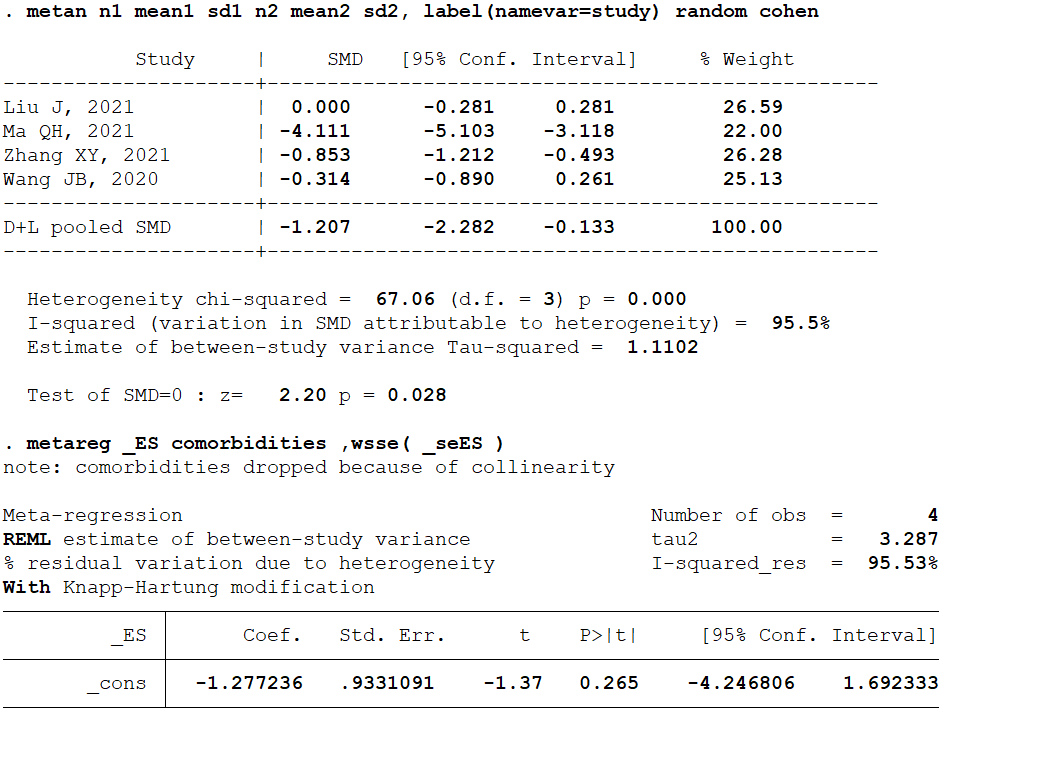


(a)


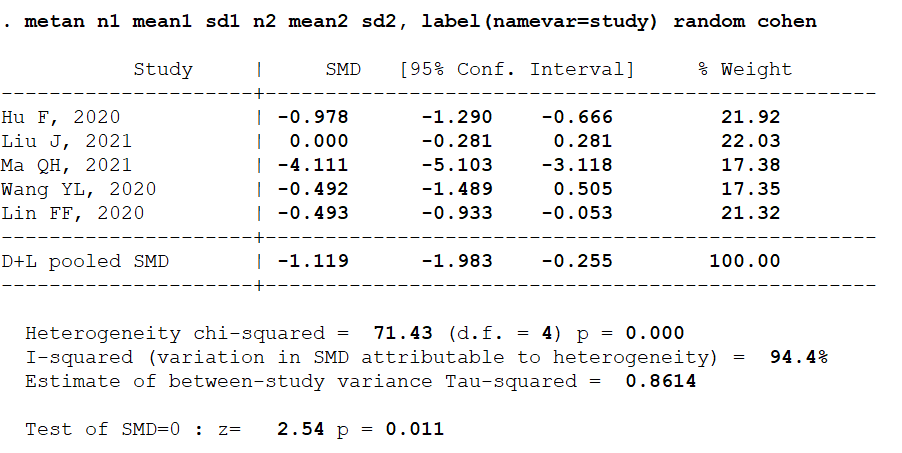


(b)


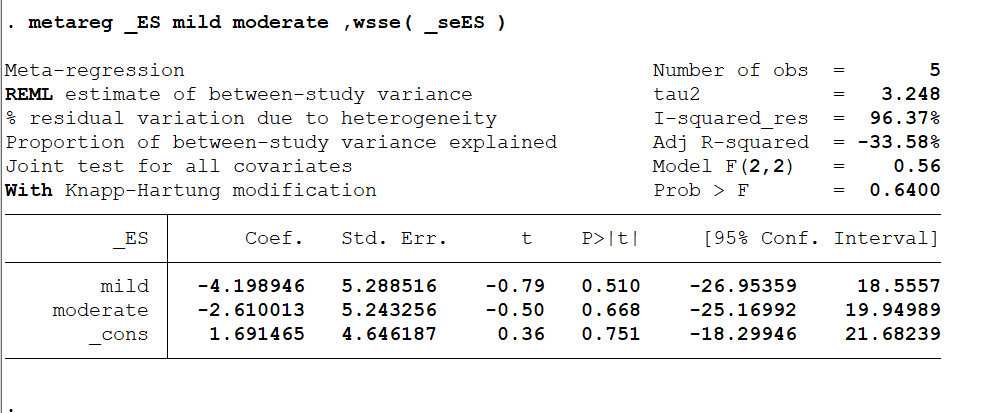


(c)

**FigureS12 Sensitivity analysis**

**12.1 Length of hospital stay-RCTs**


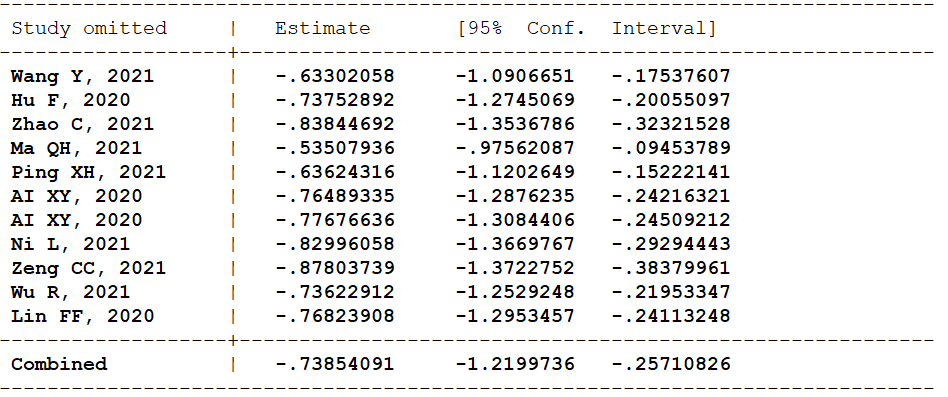


(a)


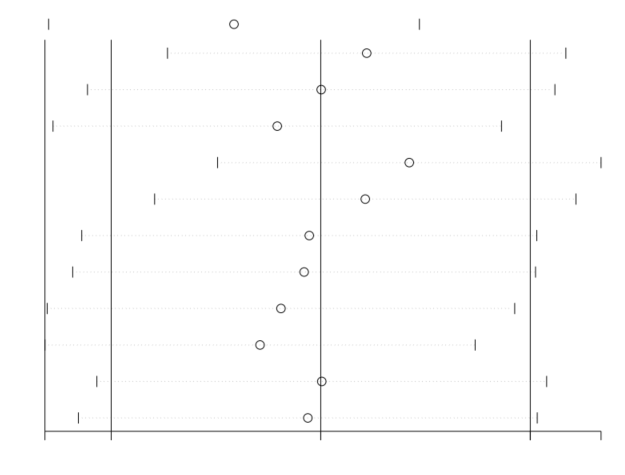


(b)

**12.2 Length of hospital stay-OBs**


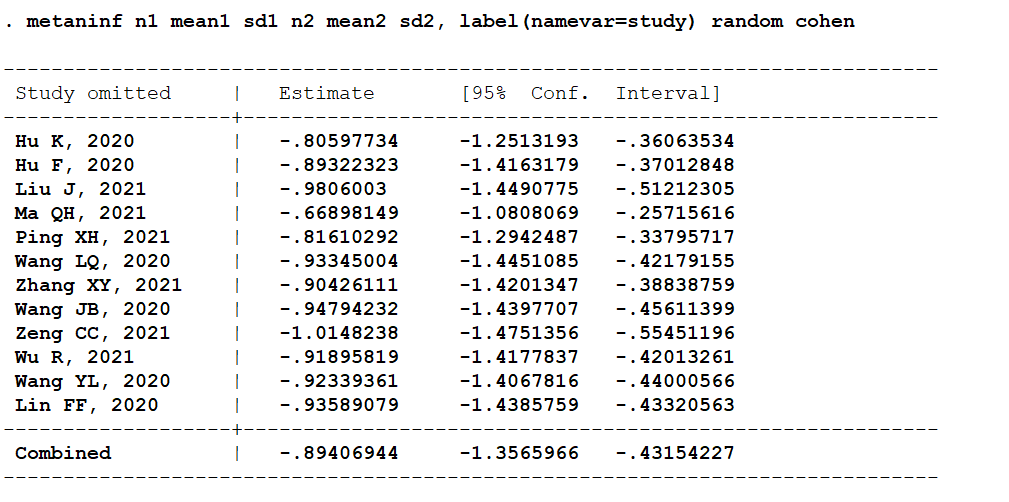


(a)


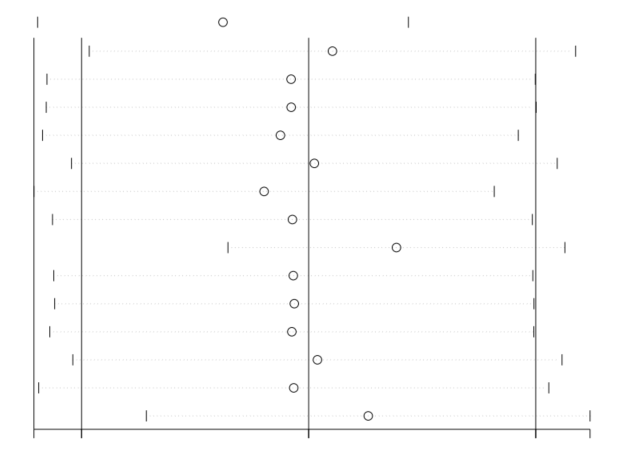


(b)

**12.3 Time to viral clearance -RCTs**


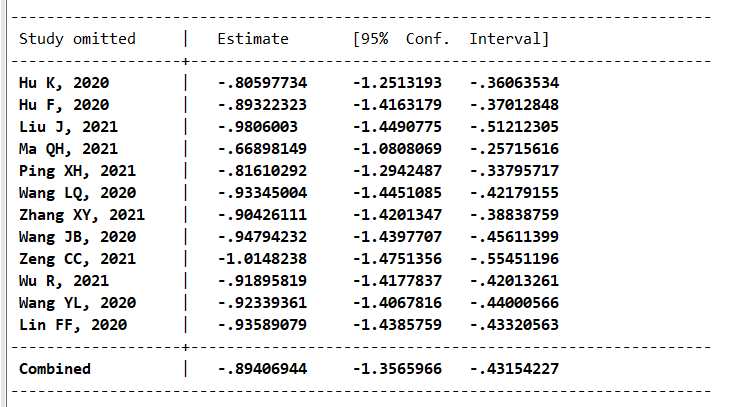


(a)


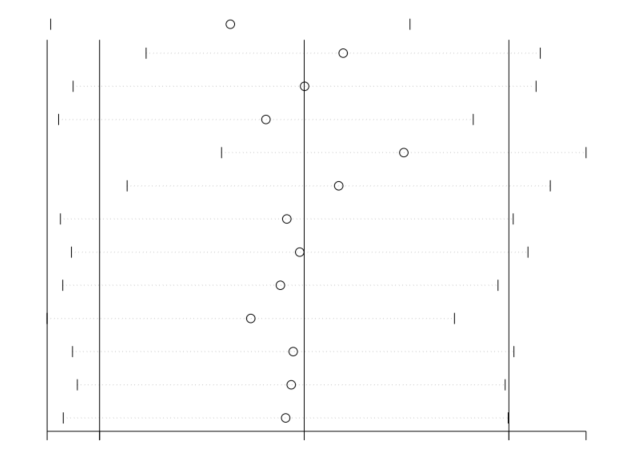


(b)
